# Development of guidelines for family and non-professional helpers on assisting an older person who is developing cognitive impairment or has dementia: a Delphi expert consensus study

**DOI:** 10.1186/s12877-016-0305-3

**Published:** 2016-07-07

**Authors:** K. S. Bond, A. F. Jorm, B. A. Kitchener, C. M. Kelly, K. J. Chalmers

**Affiliations:** Mental Health First Aid Australia, Level 6, 369 Royal Parade, Parkville, VIC 3052 Australia; Centre for Mental Health, Melbourne School of Population and Global Health, The University of Melbourne, Level 4, 207 Bouverie Street, Parkville, VIC 3010 Australia; School of Psychology, Deakin University, 1 Gheringhap Street, Geelong, VIC 3220 Australia

**Keywords:** Confusion, Dementia, Delirium, Dementia carer, Dementia guidelines, Delphi method

## Abstract

**Background:**

Assisting a person with dementia can lead to significant carer burden and possible negative outcomes for the person. Using the Delphi method, this study developed expert consensus guidelines for how family and non-professional carers should assist a person who is developing cognitive impairment, or has dementia or delirium.

**Methods:**

A systematic search of websites, books and journal articles was conducted to develop a questionnaire containing items about the knowledge, skills and actions needed for assisting a person who is developing cognitive impairment, or has dementia or delirium. These items were rated over three rounds by two international expert panels comprising professionals specialising in research or treatment of dementia, and dementia carer advocates.

**Results:**

A total of 65 participants (43 in the professional panel and 22 in the carer advocate panel) completed all three survey rounds. Of the 656 survey items that were rated, a total of 389 items were endorsed by at least 80 % of each panel. The endorsed items formed the basis of a guidelines document that explains what family and non-professional carers need to know and do when assisting a person who is developing cognitive impairment, or has dementia or delirium.

**Conclusions:**

The two groups of experts were able to reach substantial consensus on how to assist a person who is developing cognitive impairment, or has dementia or delirium.

**Electronic supplementary material:**

The online version of this article (doi:10.1186/s12877-016-0305-3) contains supplementary material, which is available to authorized users.

## Background

Given the large number of people affected worldwide [1], there is a growing need for in-home and residential care for people with dementia. Giving such care often involves consistent re-evaluation of decisions about safety, driving restrictions, healthcare, level of care and end-of-life issues [2–5], causing significant strain on carers [6, 7]. Research indicates that dementia carers are more likely than non-dementia carers to have mood and anxiety disorders, insomnia, substance use problems and physical health problems [8–12]. Carer burden not only negatively affects the carer, it may also negatively affect the person with dementia. Carer stress and decreased coping skills are associated with increased risk of institutionalisation, an increase in challenging dementia behaviours and relationship stress, and decreased survival rates [13–15]. On the other hand, positive carer coping skills have been shown to be associated with slower dementia progression [16].

Two possible ways of ameliorating the negative affects of carer burden are earlier detection and diagnosis of dementia, and carer training. For a person developing dementia, the initial period between the first manifestation of the illness and a formal diagnosis can be lengthy, with one estimate giving an average of 21.5 months [17]. Early diagnosis and intervention may be helpful in not only slowing the progression of dementia, but also in reducing the negative personal impact of dementia on the carer and the person with dementia [18]. For example, in a longitudinal study, carers who were able to recognise the early symptoms of dementia and seek a diagnosis, as opposed to those who recognised symptoms after or at the same time as diagnosis, were less likely to show signs of poor adaptation to the stressors of caring and also less likely to later place their loved one in institutionalised care [19]. Furthermore, carer training designed to alleviate carer distress and improve carer coping skills has been found to lead to longer survival time of the person with dementia and delay of admission to institutional care at 5 and 8 year follow-up [20]. Furthermore, carers who receive training that focuses on increasing carer competence and coping skills report fewer behavioural problems and slower decline of independence in the person with dementia [21, 22]. These studies indicate that training may improve the quality of life for the carer and also the person with dementia.

While training of family carers is now well established, there is a need for greater skills across the whole community in how to support a person affected by dementia. There has been a worldwide movement to increase the skills of members of the public in providing assistance to people with mental health problems through Mental Health First Aid (MHFA) training [23]. MHFA training increases knowledge about mental health problems, decreases participants’ stigmatising attitudes, and increases the likelihood that they will provide appropriate first aid actions [24]. However, to date this form of training has not been extended to cover helping an older person who is developing cognitive impairment or has dementia, or delirium.

The content of MHFA training has been based on expert consensus guidelines developed using the Delphi method [25–36]. In order to extend the content of MHFA training to cover how to assist an older person who is developing or has dementia, or delirium, we have carried out the current study. The aim was to develop guidelines that would be useful for persons in family or paid caring roles, as well for members of the public who may find themselves in a situation where they can assist.

For the purposes of this study, *older person* is defined as a person who is 65 years of age or older. The term *helper* is used, rather than carer, to include those who may not be in a formal caring role (i.e. family or paid carer), but who still provide care (e.g. family members, neighbours, friends who may provide respite or occasional care).

## Methods

The Delphi process [37] is an expert consensus method that can be used to develop best practice guidelines using practice-based evidence. Development of the current guidelines involved four steps: (1) formation of the expert panels, (2) literature search and survey development, (3) data collection and analysis, and (4) guidelines development.

### Step 1: Panel formation

As described by Hasson et al. [38], the Delphi method often involves the use of one expert panel, usually professionals working in the area of study. However, more recent work in the mental health field has included multiple expert panels, including consumers and carers, allowing for lived experience expertise to influence guidelines development. This current study utilised two expert panels: (1) health professionals specialising in research or treatment of dementia, and (2) carers of people with dementia who are in an advocacy role. Requiring carer panel members to be in an advocacy role helps to ensure that they have a breadth of experience to draw from rather than just their own personal experience. The aim was to recruit a minimum of 30 people to each panel, with panels of 20 or more members generally required to get stable results [37]. The inclusion criteria for this study were:

- Be 18 years old or older, AND
- Currently or in the past care(d) for a person who experienced dementia or delirium, AND are/been a member of a carers’ group or an advocacy organisation that relates to your carer experience, OR
- Have a minimum of 2 years’ experience specialising in research or treatment of dementia.

Participants were recruited by sending advertising flyers to Australian and international dementia organisations, support groups and research centres.

### Step 2: Literature search and survey development

In order to inform the content of the initial survey, a systematic search of the ‘grey’ and academic literature was conducted in February to March, and October to December 2013 to gather statements about how to help an older person who is developing cognitive impairment or has dementia, or delirium. The search was conducted using Google Australia, Google UK, Google USA, Google Books and Google Scholar. The key search terms were (elderly OR aged AND dementia AND help OR communicate OR care* OR respond OR support OR tips), (elderly OR aged AND delirium AND help OR communicate OR care* OR respond OR support OR tips), (elderly OR aged AND confusion OR disoriented OR attention OR memory OR mood OR alertness OR hallucination AND help OR communicate OR care* OR respond OR support OR tips), and (older person OR elderly OR aged AND Alzheimer*AND help OR communicate OR care* OR respond OR support OR tips), (seeing a doctor AND confused OR dementia OR Alzheimer AND when doctor), and (confused OR dementia OR Alzheimer AND discussing OR talking about AND diagnosis OR loss OR dying).

**Fig. 1:**
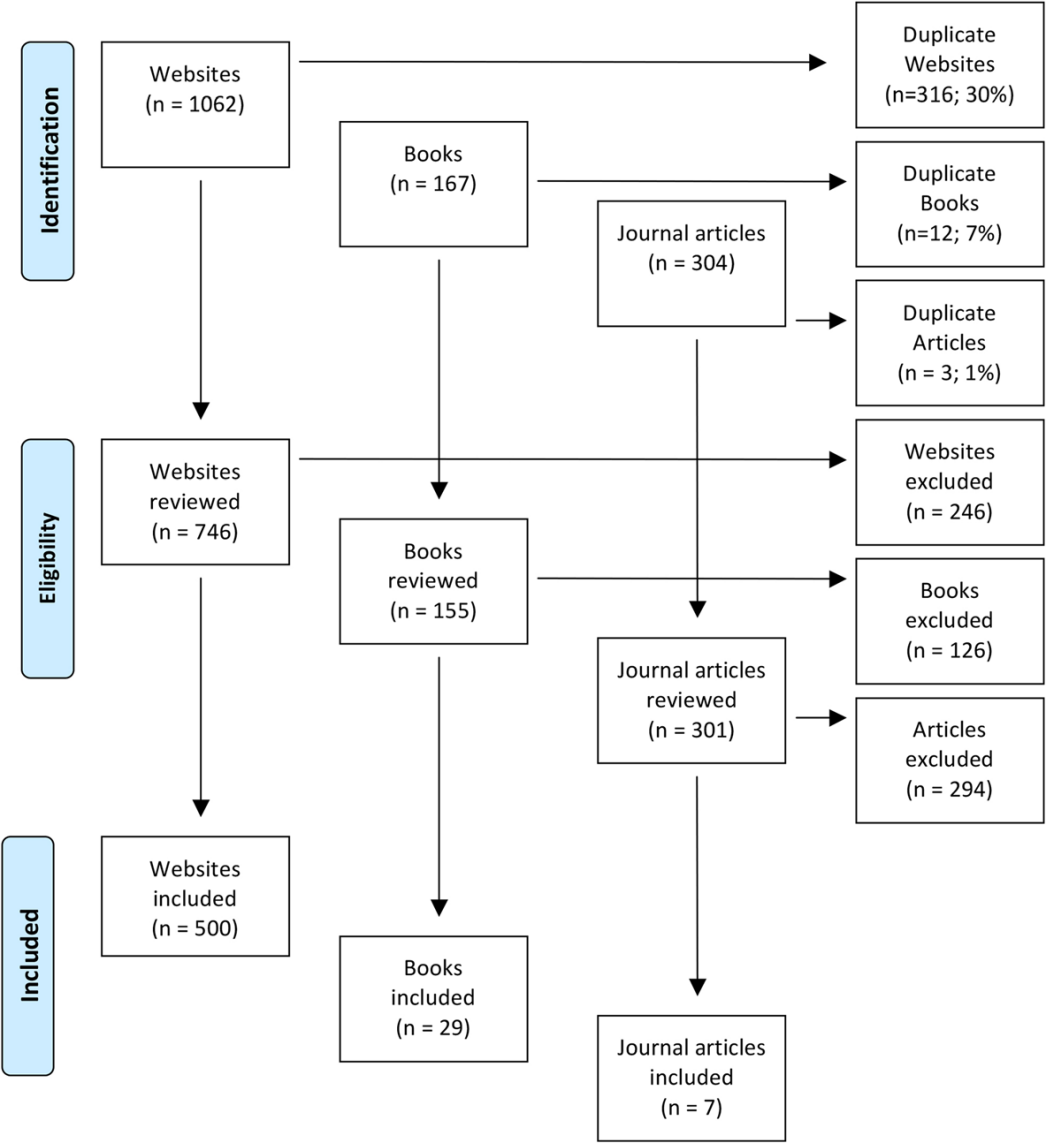
Results of the literature search

As per other similar Delphi studies (e.g. [27, 33, 36]), the first 50 websites, books and peer-reviewed journal articles for each search term were retrieved and reviewed for relevant information, after duplicates were excluded. The decision to only examine the first 50 websites, books and journal articles for each search term is based on previous Delphi studies that found that the quality of the resources declined rapidly after the first 50 [39]. Any links appearing on the websites were also reviewed. Websites, articles and books were excluded if they did not contain information about how a non-professional can support an older person who is developing cognitive impairment, or has dementia or delirium. The content of a total of 500 websites, 29 books and 7 journal articles was analysed to develop the Round 1 survey. Figure 1 summarises the results of the literature search.

A working group, consisting of staff from Mental Health First Aid Australia and the University of Melbourne, translated the relevant information from the literature search into helping statements that were clear, actionable, and contained only one idea. The statements were used to form the Round 1 survey that was administered to the expert panels via SurveyMonkey. The panel members were asked to rate each of the helping statements, using a 5-point scale (‘essential’, ‘important’, ‘don’t know/depends’, ‘unimportant’ or ‘should not be included’), according to whether or not they thought the statement should be included in the guidelines. See Additional file 1 for a copy of the surveys.

### Step 3: Data collection and analysis

Data were collected in three survey rounds administered between October 2013 and July 2015. In Round 1, panel members completed the survey developed using the literature search, and also had the opportunity to provide qualitative data in the form of comments or suggestions for new helping statements. The qualitative data was analysed using qualitative analysis software (TAMS Analyzer).

After panel members completed a survey round, the statements were categorised as follows:

1. Endorsed. The item received an ‘essential’ or ‘important’ rating from 80–100 % of members of both panels.
2. Re-rate. The item received an ‘essential’ or ‘important’ rating from 70–79 % of members from both panels, or an ‘essential’ or ‘important’ rating from 70–79 % of members from at least one panel and above 80 % from the other panel.
3. Rejected. The item did not fall into either the endorsed or re-rate categories.

The participants’ comments were thematically analysed and the working group created new items to cover helping ideas that were not included in the first survey. This new content was translated into clear and actionable statements for the Round 2 survey.

Panel members were given a summary report of Round 1 that included a list of the items that were endorsed and rejected, as well as the items that were to be re-rated in the next round. The report included the panel percentages of each rating, as well as the panel member’s individual scores for each item to be re-rated. This allowed the participants to compare their ratings with each expert panel’s consensus rating and consider whether to maintain or change their answer when re-rating an item.

The procedures for Rounds 2 and 3 were the same as described above with several exceptions. Round 2 included new items from the Round 1 comments, as well as items to be re-rated, whereas Round 3 only included the re-rated items from Round 2. There was no opportunity for comments in Round 2 or Round 3, and if a re-rated item did not receive an ‘essential’ or ‘important’ rating by 80 % or more of each panel, it was rejected. Round 3 consisted of any new items in Round 2 that needed to be re-rated, according to the above criteria.

Although not originally planned, Round 3 also included one new item that was derived from a newly published book about challenging sexual behaviours. The original literature search did not reveal many items about this topic and the working group thought that, if endorsed by the panels, it would make for a more comprehensive set of guidelines. This item received endorsement in Round 3, and therefore did not require re-rating in another survey round.

### Step 4: Guidelines development

All of the endorsed statements were written, by the first author, into the guidelines document. This was done by grouping similar items and re-writing them into continuous prose for ease of reading. Where possible, statements were combined and repetition was deleted. Original wording of the items was retained as much as possible. Some items were given examples and explanatory notes to clarify the advice. The working group reviewed this draft to ensure that the structure and the language were appropriate for the target audience of the guidelines. The draft guidelines were then given to panel members for final comment, feedback and endorsement.

## Results

### Participants

A total number of 80 people were recruited, with 65 completing all three rounds (see Table 1 for the breakdown of the retention rate for each of the panels). Participants who completed all three rounds were 15.4 % male and 84.6 % female, and had an average age of 52.5 years (12.99 SD, range 25–80). Participants were from Australia (58.5 %), New Zealand (32.3 %), the United States (4.6 %), Ireland (3.1 %) and England (1.5 %). The professional panel included 22 nurses, 16 doctors, 3 allied health professionals, 1 educator and 1 researcher, all with specialist geriatric or dementia training. The carer advocate panel included people who were members of their national Alzheimer’s organisation (54 %) or a carer organisation (32 %) and 14 % held a leadership position within an advocacy organisation (e.g. board member, educator).

**Table 1:** Retention rate

Panel	R1	R2	R3	Retention rate
Professional	56	43	43	76.8 %
Carer advocates	24	22	22	91.6 %
Total	80	65	65	81.2 %

R1 = Round 1 survey. R2 = Round 2 survey. R3 = Round 3 survey

### Item ratings

**Fig. 2:**
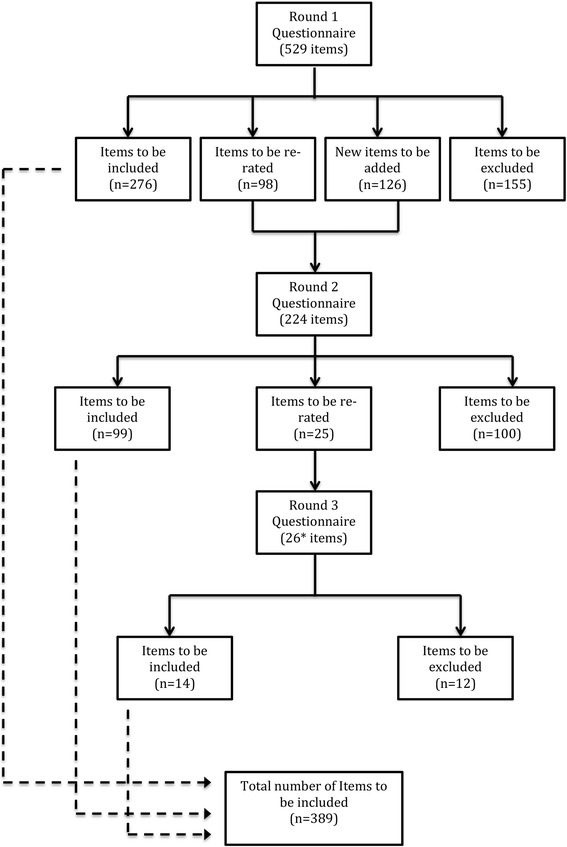
Information about the rated items. *Includes 1 item that was developed by the team as the result of a newly published resource. This item was endorsed in Round 3

A total of 656 items were rated over the three rounds to yield a total of 389 endorsed items and 267 rejected items (see Additional file 2 for a list of the endorsed and rejected items). Figure 2 presents the information about the total number of items rated, endorsed and rejected over the three rounds. The endorsed items formed the basis for the guidelines.

### Differences between expert panels

There was a strong positive correlation between the two panels in the percentage endorsement for whether items should be included in the guidelines (*r* = 0.81). However, there were also some areas of disagreement. Items that were rejected by one panel but endorsed by the other, and that received notably higher or lower rating (±10 %), are noted below. A 10 % difference was chosen, as this was used in previous studies [36, 40, 41].

### Items rejected by the professional panel by ±10 %

Forty-nine items that were endorsed by the carer advocate panel received a lower rating from the professional panel. A quarter of these items were related to driving and another quarter related to challenging behaviours and sensitive topics. After thematic analysis of the professionals’ comments, the reasons for the rejection of the items were identified as: (1) actions being perceived as requiring the helper to act in the role of a professional and (2) actions that may be appropriate for some helpers (e.g. family members), but not others (e.g. paid carers). See Table 2 for some of the professional panel comments that support these findings.

**Table 2:** Examples of professional panel members’ comments about areas of difference

Finding	Supporting comments
Action being perceived as requiring helper to act in the role of a professional	“I think it’s important to add in that fitness to drive is a medical decision, not a personal one. Family can be vital in facilitating how smoothly the transition is made, but they don’t decide.”
	“The helper’s role is supportive, not responsible for all these aspects.”
	“Will NOT always be appropriate for the helper to discuss these topics. Think it is important for helper to be aware of these issues but not to automatically assume responsibility…The helper knowledge of these issues may be very limited.”
	“This information would be likely to come from a registered health professional rather than a helper in the home environment.”
Action that may be appropriate for some helpers, but not others	“Depends who helper is. If it’s family member they should not be having to make judgements re driving safety and stopping driving.”
	“I think the role of the helper in making decisions about driving very much depends on their relationship with the person.”
	“The role a particular helper has in making the person aware (or not) of his/her diagnosis depends very much on the relationship between them and the legal standing of the helper—next of kin…etc).”
	“This also depends on the relationship of the helper, contractual obligations and the individual person with dementia.”

### Items rejected by the carer advocate panel by ±10 %

Nine items that were endorsed by the professional panel received a lower rating from the carer advocate panel. The majority of these items (56 %) were about including the person in decisions and discussions about diagnosis, care and living arrangements. The remaining items related to accessing support groups to help lessen the impact of giving up driving, challenging behaviours, and hallucinations and delusions. After thematic analysis of the carers’ comments, using qualitative analysis software, the reasons for the rejection of the items were identified as: (1) not falling within the role of the helper and (2) strategies that may not be helpful as the disease progresses. See Table 3 for some of the carers’ comments that support these findings.

**Table 3:** Identified reasons for rejection by carer advocate panel

Theme	Supporting comments
Role of helper	The helper should not unilaterally discuss diagnosis without clear guidance and understanding of how to engage in the discussion.
	I firmly believe that a medical professional needs to inform the person of the diagnosis, because some people may not accept it coming from their helper.
Disease progression	This would be OK if the person is at a stage of understanding these matters.
	This is fine at early stages, but at later stages it may just add whole lot of anxiety.

### Guidelines development

The endorsed items were used as the basis for the guidelines for helping an older person who is developing cognitive impairment or has dementia, or delirium. They are available from: mhfa.com.au/resources/mental-health-first-aid-guidelines [42]. Table 4 presents the main themes and subthemes of the guidelines.

**Table 4:** Themes and sub-themes of the guidelines

Themes	Sub-themes
What is confusion and dementia?	
What to do if you are concerned that a person may be developing dementia	• Talking to the person • Seeking professional help • If the person is reluctant to get help
Supporting the person with dementia	• Seeing the person behind the dementia • Helping the person with their memory problems • Helping the person to complete tasks • Helping the person who is disoriented • Helping the person who has regressed into the past
Communicating with the person	• Gaining and keeping the person’s attention • Being understood during a conversation • Communicating in a group situation • Asking the person questions • Offering the person options • Non-verbal communication • Challenges experienced during communication
Discussing sensitive issues	• Discussing the diagnosis • Making decisions and planning for the future • Discussions and decisions about driving • Discussions and decisions about care
Behaviours that you may find challenging	• Resistance • Arguments • Agitation • Anger and aggression • Disinhibited and inappropriate sexual behaviour • Delusions and hallucinations
Assisting someone who is wandering	• How to tell if a person is wandering • What to do if you encounter someone who is wandering • Ways of identifying people who wander • Contacting emergency services
Delirium	• Delirium is a medical emergency • If you are caring for a person with delirium

## Discussion

This research aimed to develop a set of guidelines on how family and non-professional helpers can assist an older person who is developing cognitive impairment, or has dementia or delirium. Overall, 389 items were endorsed by both expert panels as important or essential to be included in the guidelines. The endorsed items were written into a guidelines document that is available to the public from the Mental Health First Aid Australia website (mhfa.com.au). A strength of the guidelines is that they address a wide variety of topics or situations that a person may encounter when assisting an older person with dementia. In particular, it addresses a number of issues that have been identified as particularly challenging for carers of people who have dementia [17, 43].

### Approaching and talking to the person

These guidelines include advice on how to approach and talk to a person about a decline in cognitive functioning, including what to do if the person is reluctant to admit to cognitive problems or seek professional help. Often it will be family, friends or neighbours who first notice a person’s decline in cognitive function [17]. Mental Health First aid training based on guidelines developed in a similar way has been shown to increase course participants’ ability to recognise mental health problems in a vignette and to reduce their stigmatising attitudes [24, 41]. It is hoped that training based on these guidelines will help course participants more readily recognise the signs of, and reduce the stigma associated with dementia, promoting earlier help seeking.

### Challenging behaviours

This research developed a list of strategies that a helper can use when they encounter challenging behaviours in the person with dementia. They include strategies for managing resistance, arguments, agitation, anger, aggression, inappropriate sexual behaviour, delusions and hallucinations. Challenging behaviours have a pervasive negative effect on carers, as compared to other challenges experienced, e.g. assisting with daily needs [43]. Research suggests that interventions that target carer knowledge and skills, such as these guidelines, are one important way to reduce carer burden and the resultant negative health effects on both the carer and the person with dementia [14, 44].

### Decision making

Making decisions on behalf of a loved one has been identified as an area of significant distress for carers of people with dementia [4] and these guidelines include advice on discussing and making decisions about sensitive topics, such as moving to a higher level of care, planning for the future and driving cessation. The decision to change to a higher level of care can be emotionally difficult for carers [45], and decision aids, similar to these guidelines, may be helpful. Although the research into the usefulness of decision aids is limited, one study reported that carers found them helpful and that they reduced subjective carer burden [46].

The results of this study indicate that while helpers are an important supportive factor in driving safety and cessation, it is the medical professional’s responsibility to make the decision for when the person should stop driving. One important way that a helper may be involved in decisions around driving is to report unsafe driving to the health professional. This means that it is important that helpers have some guidance on discussing and making decisions about driving. Furthermore, this research recommends and gives guidance on how helpers can take a proactive role in planning for driving cessation, which may help make the transition to not driving easier for the person with dementia [47].

### Crisis situations

Two crisis situations that are covered by these guidelines are wandering and delirium, both of which can lead to significant harm and even death if not addressed appropriately [48]. The majority of the literature on wandering is focused on assessing and managing wandering behaviour from the perspective of a care facility or a family carer (e.g. [49]). Our guidelines diverge from the literature in that they provide ‘first aid’ advice on what family and non-professional helpers or a member of the public can do if they encounter someone who is wandering. The advice includes how to recognise and approach a person who is wandering, ways to ascertain the person’s identity, and how and when to get emergency services involved.

Delirium commonly occurs in people with dementia, with a prevalence rate of between 22 % and 89 % in community and hospital populations [50]. The long-term outcomes of delirium in a person with dementia include increased cognitive and functional decline, nursing home placement and death [51, 52]. Given these poor outcomes, it is important that delirium is recognised and treated quickly. It is hoped that these guidelines will improve the ability of helpers to recognise, seek treatment for and assist in managing delirium, reducing the negative long-term consequences.

### Limitations

A limitation of this study was the size of the carer advocate panel. A panel size of 23 has been found to yield stable results in a simulation study (Akins et al. 2005). While there were 24 experts in the carer advocate panel in Round 1, this reduced to 22 experts in subsequent rounds.

People with dementia were not invited to participate in this research—a notable limitation, but it was impossible to screen cognitive ability of participants to determine if they were able to meaningfully complete the surveys. Another limitation is the possibility that some panel members were asked to advise on statements that were beyond their expertise, possibly leading to a lack of inclusion of useful items. Furthermore, while participants were able to provide comments in Round 1 of the survey, they were not able to discuss their comments and opinions with others. Panel members may have held biases or made incorrect assumptions that were unchallenged because there was no opportunity for discussion. It is possible that key actions were omitted from the guidelines because of this.

Another possible limitation is the exclusion of items that would be helpful at certain stages of dementia, but were rejected because they received a ‘depends’ rating from the majority of participants. Furthermore, the majority of the panel members were female and mostly from Australia and New Zealand, which may have introduced bias. Finally, these guidelines were developed for English-speaking Western countries and further research is needed to adapt them for other cultures.

### Implementation

Guidelines in themselves may not ensure change in supportive behaviours [37]. Therefore, these guidelines will be used to inform the contents of the Mental Health First Aid for the Older Person course. This course, due to be released later in 2016, will teach participants how to assist an older person who is developing cognitive impairment or has dementia, or delirium, as well as other mental health problems and crises. Although the course will initially be released for Australia, the training materials will also be made available to the organizations in over 20 countries that have adopted and adapted the Mental Health First Aid program from Australia.

## Conclusion

Dementia is an illness that can cause significant helper burden and stress, potentially leading to a decline in quality of life for the person with dementia and their loved ones. Expert consensus (carer advocates and professionals) was reached on 389 items that were used as the basis for a guidelines document related to assisting an older person who is developing cognitive impairment or has dementia, or delirium. These guidelines will provide guidance to helpers on a number of commonly encountered challenges; they will also inform future training for the public on how to assist an older person who is developing cognitive impairment, or has dementia or delirium. It is hoped that this training will lead to a decrease in the burden experienced by people with dementia and their carers.

## Abbreviations

MHFA, mental health first aid

## Additional files

Additional file 1: Survey. Copies of the Round 1, 2 and 3 surveys. (PDF 2342 kb) Additional file 2: Endorsed and rejected items. Endorsed and rejected items including the survey round that they were endorsed or rejected. (XLSX 91 kb)
