# Supplementary material for: Development of guidelines for family and non-professional helpers on assisting an older person who is developing cognitive impairment or has dementia: a Delphi expert consensus study
Source: BMC Geriatr. 2016 Jul 7;16:129. doi: 10.1186/s12877-016-0305-3 (PMC4936264; doi:10.1186/s12877-016-0305-3)
Supplement: Additional file 1: — Survey. Copies of the Round 1, 2 and 3 surveys. (PDF 2342 kb) [file 12877_2016_305_MOESM1_ESM.pdf]

Helping the confused older person

INTRODUCTION

Purpose of this research

There is a need for research that focuses specifically on giving assistance to the older person experiencing confusion or cognitive changes associated with dementia, delirium or another condition. The purpose of this project is to develop a set of guidelines that advise how someone (a family member, friend, neighbour, concerned community member or a paid carer without specialist qualifications) can help a confused person.

Why have I been selected?

You have been selected as a panel member for this study because you have expertise with dementia and delirium (either as a carer or professional).

What do I need to do?

Your task is to rate the statements presented in this questionnaire according to how important you believe they are to assisting a confused older person. When rating the statements, please keep in mind that the guidelines will be used by the general public and as such, the statements need to be rated according to how important each one is for someone, who does not necessarily have a counselling or clinical background, to help an older person with confusion. For example you may be asked to rate the following statement:

1. The helper should not hurry the person because this can cause them anxiety.

Essential	Important	Don't know / Depends	Unimportant	Should not be included
◯	◯	◯	◯	◯

You will need to decide how important, from **'essential'** to **'should not be included'**, you think it is that this statement be included in the guidelines.

For an example of previously developed guidelines go to: First aid guidelines for assisting the person who is suicidal.

Introduction (continued)

What if something important is missing or if I have something to add?
There is a place for you to add comments at the end of each page. This is so you can add any missing items or topics you think should be in guidelines. These items will then go into a second round of this survey to be rated by all of the expert panel members.

Where do the statements in the questionnaire come from?
The statements in the following questionnaire were derived from information found online, in books and in academic journal articles. Some of the statements may seem contradictory or controversial; however, we have included them because they reflect the wide range of people's beliefs about policy, services and care. It is important to note that we do not necessarily agree with these statements; we have included them because we do not believe that we should decide what the best practice is in this area. Rather, we want an expert panel to decide this.

What is the focus of the questionnaire?
The focus of this research is to produce guidelines that will advise someone how to help an older person with confusion. The person providing help may be a family carer, friend, neighbour, concerned community member or a paid carer without specialist qualifications.

The questionnaire will focus on how to help someone developing dementia or delirium. It will also look at how to help a person who is experiencing a worsening of existing dementia symptoms or is in a crisis due to their confusion. These guidelines will not cover topics related to the medical care provided by health professionals, aspects of physical care or care provided in the later stages of dementia.

How long will it take to complete the questionnaire?
This questionnaire should take approximately 120 minutes to complete. However, some people may finish it more quickly, while others may take longer, especially if they wish to lodge comments at the end of each section.

Can I save my answers and come back to the questionnaire?
If necessary, you can complete the survey in two or more sittings. You can save your answers at any time by completing a page and clicking 'Next' at the bottom. This marks your page as complete and you can begin again at a later date on the next page. Please make sure that you always log back in using the same computer, and that cookies are enabled, otherwise the software will fail to recognise your code and previously saved responses.

Consent to Participate in this Research
It is important for you to know that participation in this study is completely voluntary. You are not under any obligation to participate and you can withdraw at any time. Submitting your questionnaire is an indication of your understanding of this and your consent to participate in the study.
We would like to thank you for your time and effort and encourage you to provide us with feedback on this process.

Best Wishes,
The Centre for Mental Health, University of Melbourne and Mental Health First Aid Australia Research Team

INFORMATION ABOUT YOU

***2.**
Area of expertise

- ◯ Carer advocate
- ◯ Professional

CARERS: INFORMATION ABOUT YOU

***3.**

Participant code

***4. You must be able to answer 'Yes' to the following questions in order to participate in this research.**

Do you currently or have you in the past care(d) for a person who experienced dementia or delirium?

☐ Yes

If you do not meet this criteria you are ineligible to participate. Please exit the survey now. Thank you for your interest.

***5. Are you a member of a carers group or an advocacy organisation that relates to your carer experience?**

☐ Yes

If you do not meet this criteria you are ineligible to participate. Please exit the survey now. Thank you for your interest.

PROFESSIONALS: INFORMATION ABOUT YOU

***6.**

Participant code

***7.**

Area of expertise

***8. You must be able to answer 'Yes' to the following question in order to participate in this research.**

Do you have at least 2-3 years clinical experience treating or caring for people with dementia or delirium?

☐ Yes

If you do not meet this criteria you are ineligible to participate. Please exit the survey now. Thank you for your interest.

INFORMATION ABOUT YOU (continued)

***9. You must be able to answer 'Yes' to this question in order to participate in this research.**

(If you do not meet this criteria you are ineligible to participate. Please exit the survey now. Thank you for your interest.)

Do you live and, if applicable, work in one of the following countries: Australia, Canada, Ireland, New Zealand, the UK or the USA?

◯ Yes

***10. You must be able to answer 'Yes' to this question in order to participate in this research.**

(If you do not meet this criteria you are ineligible to participate. Please exit the survey now. Thank you for your interest.)

Are you at least 18 years old?

◯ Yes

***11. What is your gender?**

◯ Female

◯ Male

◯ Gender queer

◯ Other

***12. What is your age?**

***13. If applicable, what is your occupation and title?**

***14. Please name all the relevant organisations you are affiliated with and your role within these organisations, e.g. professional organisations, consumer advocacy groups.**

***15. Please provide details of your primary place of work, study or residence.**

City/Town

State/Province

Country

***16. Consent**

I understand that by submitting this questionnaire I am giving my consent to participate in this study.

◯ Yes

Overview of Questionnaire

The questionnaire is divided into the following sections:

1. General awareness

2. Memory and other cognitive problems

3. Seeking help

4. Encountering someone wandering

5. Communication

6. Challenges you may encounter during communication

7. Discussing sensitive issues and making decisions with the person

8. Challenging behaviours

9. Delirium

Definitions

These terms may have different meanings for participants. The definitions below are how these terms are used in this survey. When completing the survey, please ensure you use these definitions only.

Helping the confused older person

Person in this survey refers to an **older person who is experiencing confusion** which may be due to dementia, delirium or other conditions. Older person in this survey refers to those aged 65 or older, however it is expected that the resulting guidelines may also be relevant to assisting adults with confusion who are younger.

Mental health first aid for the confused older person is the help offered to a person who may be developing dementia or delirium, is experiencing a worsening of existing dementia symptoms or is in a crisis due to their confusion.

Helper in this survey refers to the individual who provides mental health first aid as defined above. **The helper may be a family member, friend, neighbour, concerned community member or a paid carer without specialist qualifications.**

Confusion in this survey is a broad term that refers to a decline in normal cognitive ability, which may vary from mild to severe. The cognitive changes may be associated with dementia or delirium. It may include a number of the following signs and symptoms: lack of alertness, poor attention span, disorientation to time and place, trouble following a conversation, unclear or illogical speech, impaired short-term memory, difficulty in planning and carrying out tasks, inappropriate behaviour, disconnection from reality or delusional beliefs.

Dementia is a condition involving progressive decline of cognitive abilities such as short-term memory, language and the ability to plan and carry out tasks. Dementia is an umbrella term for a large group of illnesses that cause this progressive decline. The symptoms appear over months and years. The initial signs of confusion may be mild, or may be only apparent at certain times or in certain situations, but the condition tends to get worse over time.

Delirium is a condition where a sudden and obvious worsening of a person's usual level of functioning appears over hours or days. Delirium can involve problems with attention, awareness, orientation to environment and other areas of cognitive functioning. Delirium is caused by an underlying disease or environmental factors, such as medication.

General awareness

This section asks you about what general information the helper should know about confusion, dementia and delirium. Please rate how important (from 'essential' to 'should not be included') you think it is that each statement be included in the guidelines.

When providing first aid to the confused person, the helper will need to use their judgement because a person's decline in cognitive ability may be very mild or more severe. Please keep this in mind when rating the following items.

Please also keep the **definitions** in mind when rating the statements.

Confusion, dementia and delirium

* 17. The helper should be aware of the signs and symptoms of confusion.

Essential	Important	Don't know/Depends	Unimportant	Should not be included
○	○	○	○	○

* 18. The helper should be aware of the signs and symptoms of dementia.

Essential	Important	Don't know/Depends	Unimportant	Should not be included
○	○	○	○	○

***19. The helper should be aware of the signs and symptoms of delirium.**

Essential	Important	Don't know/Depends	Unimportant	Should not be included
◯	◯	◯	◯	◯

***20. The helper should be aware that the person with signs or symptoms of delirium may be at risk of a medical emergency.**

Essential	Important	Don't know/Depends	Unimportant	Should not be included
◯	◯	◯	◯	◯

***21. The helper should be aware that although dementia or delirium more commonly occur in an older person, they can occur in a younger person as well.**

Essential	Important	Don't know/Depends	Unimportant	Should not be included
◯	◯	◯	◯	◯

***22. The helper should be aware that dementia is not a normal part of ageing.**

Essential	Important	Don't know/Depends	Unimportant	Should not be included
◯	◯	◯	◯	◯

The person

***23. The helper should be aware that the person may still retain much of their intellectual capacity.**

Essential	Important	Don't know/Depends	Unimportant	Should not be included
◯	◯	◯	◯	◯

***24. The helper should be aware that the person may still have the desire to be a contributing member of society.**

Essential	Important	Don't know/Depends	Unimportant	Should not be included
◯	◯	◯	◯	◯

***25. The helper should be aware that the person has a sense of self, personality, thoughts and feelings.**

Essential	Important	Don't know/Depends	Unimportant	Should not be included
◯	◯	◯	◯	◯

Helping the confused older person

***26. The helper should think of the person they are helping as a person with dementia and not a demented person.**

Essential	Important	Don't know/Depends	Unimportant	Should not be included
○	○	○	○	○

***27. The helper should be aware that the person may be aware of a loss of skills and may be frustrated with their increasing dependence on others.**

Essential	Important	Don't know/Depends	Unimportant	Should not be included
○	○	○	○	○

***28. The helper should be aware that the person's feelings and behaviours will be affected by the negative actions of others, e.g. patronising or angry behaviour.**

Essential	Important	Don't know/Depends	Unimportant	Should not be included
○	○	○	○	○

***29. The helper should be aware that changed behaviour in the person is not anyone's fault, but is the result of changes in the brain.**

Essential	Important	Don't know/Depends	Unimportant	Should not be included
○	○	○	○	○

30. Do you have any comments on the above statements? Is there anything you would like to add? Please write your suggestions in the box provided.

Memory and other cognitive problems

This section asks you what information the helper should know about memory and other cognitive problems. It also asks how the helper can assist the person with these limitations. Please rate how important (from ‘essential’ to ‘should not be included’) you think it is that each statement be included in the guidelines.

When providing first aid to the confused person, the helper will need to use their judgement because a person's decline in cognitive ability may be very mild or more severe. Please keep this in mind when rating the following items.

Please also keep the **definitions** in mind when rating the statements.

Confusion, dementia and delirium

Understanding changes in memory

Helping the confused older person

***31. The helper should be aware that major changes in memory are not a normal part of ageing.**

Essential	Important	Don't know/Depends	Unimportant	Should not be included
○	○	○	○	○

***32. The helper should be aware that the person may have difficulty remembering much, or any, new information.**

Essential	Important	Don't know/Depends	Unimportant	Should not be included
○	○	○	○	○

***33. The helper should be aware that the person may forget the first part of an explanation by the time the helper finishes it.**

Essential	Important	Don't know/Depends	Unimportant	Should not be included
○	○	○	○	○

***34. The helper should be aware that even though the person has failing memory or reduced understanding, it does not mean that they do not know how they feel in the here and now.**

Essential	Important	Don't know/Depends	Unimportant	Should not be included
○	○	○	○	○

***35. The helper should be aware that even though the person has failing memory or reduced understanding of a conversation, the person still has resultant feelings and emotions which may last for many hours.**

Essential	Important	Don't know/Depends	Unimportant	Should not be included
○	○	○	○	○

***36. The helper should be aware that the person is more likely to live in the moment as their memory fails.**

Essential	Important	Don't know/Depends	Unimportant	Should not be included
○	○	○	○	○

Helping the confused older person

***37. If the person has forgotten that they have done something and keeps asking to do it (e.g. attending a doctor's appointment), the helper should distract them.**

Essential	Important	Don't know/Depends	Unimportant	Should not be included
◯	◯	◯	◯	◯

***38. If the person does not recognise what an object is for, the helper should not explain because that would result in a sense of failure, as well as confusion and frustration.**

Essential	Important	Don't know/Depends	Unimportant	Should not be included
◯	◯	◯	◯	◯

***39. If the person has regressed into the past, the helper should not dismiss their resultant feelings and thoughts.**

Essential	Important	Don't know/Depends	Unimportant	Should not be included
◯	◯	◯	◯	◯

***40. The helper should be aware that regressions into the person's past are felt as actual current experiences for the person.**

Essential	Important	Don't know/Depends	Unimportant	Should not be included
◯	◯	◯	◯	◯

41. Do you have any comments on the above statements? Is there anything you would like to add? Please write your suggestions in the box provided.

Memory and other cognitive problems (continued)

This section asks you what information the helper should know about memory and other cognitive problems. It also asks how the helper can assist the person with these limitations. Please rate how important (from 'essential' to 'should not be included') you think it is that each statement be included in the guidelines.

When providing first aid to the confused person, the helper will need to use their judgement because a person's decline in cognitive ability may be very mild or more severe. Please keep this in mind when rating the following items.

Please also keep the **definitions** in mind when rating the statements.

Completing tasks and making decisions

Helping the confused older person

***42. The helper should allow the person plenty of time to complete tasks.**

Essential	Important	Don't know/Depends	Unimportant	Should not be included
◯	◯	◯	◯	◯

***43. The helper should break down tasks into small, simple, concrete steps for the person.**

Essential	Important	Don't know/Depends	Unimportant	Should not be included
◯	◯	◯	◯	◯

***44. The helper should offer help rather than just doing a task for the person, so that the person does not feel that they are being treated like a child or incapable.**

Essential	Important	Don't know/Depends	Unimportant	Should not be included
◯	◯	◯	◯	◯

***45. If the person is struggling with a task (e.g. dressing), the helper should intervene only when the person asks for help, even if this means that the task is not done as well as it used to be.**

Essential	Important	Don't know/Depends	Unimportant	Should not be included
◯	◯	◯	◯	◯

***46. If the person appears to be frustrated by a task, the helper should divert their attention before the stress becomes overwhelming.**

Essential	Important	Don't know/Depends	Unimportant	Should not be included
◯	◯	◯	◯	◯

***47. The helper should not give the person too many things to do at once, as this will increase their level of anxiety.**

Essential	Important	Don't know/Depends	Unimportant	Should not be included
◯	◯	◯	◯	◯

***48. The helper should not hurry the person because this can cause them anxiety.**

Essential	Important	Don't know/Depends	Unimportant	Should not be included
◯	◯	◯	◯	◯

Helping the confused older person

***49. The helper should attempt to reduce any stress on the person because stress can increase confusion.**

Essential	Important	Don't know/Depends	Unimportant	Should not be included
◯	◯	◯	◯	◯

***50. The helper should be aware that the person may become more confused if they are offered too many options at once.**

Essential	Important	Don't know/Depends	Unimportant	Should not be included
◯	◯	◯	◯	◯

***51. When the person needs to make a decision, the helper can assist by limiting any choice to two options.**

Essential	Important	Don't know/Depends	Unimportant	Should not be included
◯	◯	◯	◯	◯

Memory loss during conversation

***52. If the person complains that they cannot remember anything, the helper should acknowledge it rather than dismiss it (e.g. say "It must be frustrating.").**

Essential	Important	Don't know/Depends	Unimportant	Should not be included
◯	◯	◯	◯	◯

***53. If the person wants to talk about the changes in memory they are noticing, the helper should take the time to listen to them.**

Essential	Important	Don't know/Depends	Unimportant	Should not be included
◯	◯	◯	◯	◯

***54. If the person complains that they cannot remember anything, the helper should give the person a chance to talk about their memory loss, unless it seems to upset the person more than it helps them.**

Essential	Important	Don't know/Depends	Unimportant	Should not be included
◯	◯	◯	◯	◯

Helping the confused older person

***55. If the person talks about deceased people as though they are alive (e.g. a parent who has died long ago), rather than tell them that the person is dead, the helper should steer the conversation towards something else (e.g. asking the person what their parents were like or talking about something unrelated).**

Essential	Important	Don't know/Depends	Unimportant	Should not be included
◯	◯	◯	◯	◯

56. Do you have any comments on the above statements? Is there anything you would like to add? Please write your suggestions in the box provided.

Seeking help

This section asks you what information the helper should know about when and how the helper can assist the person to get professional help. Please rate how important (from 'essential' to 'should not be included') you think it is that each statement be included in the guidelines.

When providing first aid to the confused person, the helper will need to use their judgement because a person's decline in cognitive ability may be very mild or more severe. Please keep this in mind when rating the following items.

Please also keep the **definitions** in mind when rating the statements.

***57. The helper should be aware that early detection of dementia has benefits for the person's treatment and long-term management.**

Essential	Important	Don't know/Depends	Unimportant	Should not be included
◯	◯	◯	◯	◯

When to see a doctor

***58. The helper should be aware that changes in memory and other signs or symptoms do not necessarily indicate that the person has dementia, but may be related to other treatable health problems.**

Essential	Important	Don't know/Depends	Unimportant	Should not be included
◯	◯	◯	◯	◯

Helping the confused older person

***59. If a person is experiencing major changes in memory, the helper should encourage them to see a doctor sooner rather than later.**

Essential	Important	Don't know/Depends	Unimportant	Should not be included
○	○	○	○	○

***60. If the helper suspects that the person has dementia, they should keep a record of any behavioural or memory changes that they notice in order to show a doctor later on.**

Essential	Important	Don't know/Depends	Unimportant	Should not be included
○	○	○	○	○

***61. The helper should encourage the person to see a doctor if:**

	Essential	Important	Don't know/ depends	Unimportant	Should not be included
*the person has any signs or symptoms of dementia.	○	○	○	○	○
*the person has several signs or symptoms of dementia.	○	○	○	○	○
*there have been ongoing changes in the person's memory and functioning.	○	○	○	○	○
*others in the person's life are worried about changes in the person's memory and functioning.	○	○	○	○	○
*the person is worried that there have been changes in their memory and functioning.	○	○	○	○	○
*the person shows worsening of challenging behaviour.	○	○	○	○	○

62. Do you have any comments on the above statements? Is there anything you would like to add? Please write your suggestions in the box provided.

Seeking help (continued)

This section asks you what information the helper should know about when and how the helper can assist the person to get professional help. Please rate how important (from ‘essential’ to ‘should not be included’) you think it is that each statement be included in the guidelines.

Helping the confused older person

When providing first aid to the confused person, the helper will need to use their judgement because a person's decline in cognitive ability may be very mild or more severe. Please keep this in mind when rating the following items.

Please also keep the **definitions** in mind when rating the statements.

Talking with the person about professional help

*63. If the helper is worried about the person's memory, the helper should have a conversation with the person about it.

Essential	Important	Don't know/Depends	Unimportant	Should not be included
○	○	○	○	○

*64. During a conversation about the person's memory and functioning, the helper should:

	Essential	Important	Don't know/ depends	Unimportant	Should not be included
*discuss the changes they have observed in the person's memory and functioning.	○	○	○	○	○
*explain that they are concerned because they care.	○	○	○	○	○
*ask the person how they are feeling about their memory.	○	○	○	○	○
*make a plan with the person to see a doctor.	○	○	○	○	○
*try not to be upset if the person refuses to accept what they are saying.	○	○	○	○	○
*try to make the person feel at ease and reassure them that their memory problems are not their fault.	○	○	○	○	○
*try to keep the conversation positive by focusing on the benefits of early treatment for retaining skills and strengths.	○	○	○	○	○

65. Do you have any comments on the above statements? Is there anything you would like to add? Please write your suggestions in the box provided.

Seeking help (continued)

This section asks you what information the helper should know about when and how the helper can assist the person to get professional help. Please rate how important (from 'essential' to 'should not be included') you think it is that each statement be included in the guidelines.

Helping the confused older person

When providing first aid to the confused person, the helper will need to use their judgement because a person's decline in cognitive ability may be very mild or more severe. Please keep this in mind when rating the following items.

Please also keep the **definitions** in mind when rating the statements.

Assisting the person to get professional help

***66. The helper should be aware that the person may be reluctant to visit a doctor because they may not have the insight to realise something is wrong or, if they do, they may be afraid of having their fears confirmed.**

Essential	Important	Don't know/Depends	Unimportant	Should not be included
◯	◯	◯	◯	◯

***67. The helper should be aware that the person may be reluctant to visit a doctor because they are embarrassed or upset about their memory loss.**

Essential	Important	Don't know/Depends	Unimportant	Should not be included
◯	◯	◯	◯	◯

***68. The helper should assist the person to make a doctor's appointment and accompany them to the appointment in order to raise their concerns with the doctor.**

Essential	Important	Don't know/Depends	Unimportant	Should not be included
◯	◯	◯	◯	◯

***69. If the person has a scheduled doctor's appointment, the helper should accompany them in order to raise their concerns with the doctor.**

Essential	Important	Don't know/Depends	Unimportant	Should not be included
◯	◯	◯	◯	◯

***70. If the helper plans to attend the person's appointment with the doctor and the person questions this, the helper should tell the person that they have some questions for the doctor.**

Essential	Important	Don't know/Depends	Unimportant	Should not be included
◯	◯	◯	◯	◯

Helping the confused older person

***71. If the person does not want the helper to attend the doctor's appointment with them, the helper should call or send a letter to the person's doctor advising them of any concerns.**

Essential	Important	Don't know/Depends	Unimportant	Should not be included
○	○	○	○	○

***72. If the person is reluctant to get help, the helper should:**

	Essential	Important	Don't know/ depends	Unimportant	Should not be included
*discuss the matter with the person and try to identify the reason behind their reluctance.	○	○	○	○	○
*discuss the matter with the person and help them overcome any of their worries and fear by providing care and reassurance.	○	○	○	○	○
*suggest that the person go for a general medical check-up.	○	○	○	○	○
*suggest that it is time that both the person and themselves have a medical check-up.	○	○	○	○	○
*find a physical reason for a visit to a doctor, preferably for a health problem that the person willingly acknowledges, e.g. headaches, failing eyesight.	○	○	○	○	○
*inform the person that they and the family would worry less knowing that the person has the most up-to-date information on their health.	○	○	○	○	○
*acknowledge any fear the person expresses.	○	○	○	○	○
*enlist whoever has the greatest influence on the person to encourage them to get help, e.g. a family member or good friend.	○	○	○	○	○
*encourage a health appointment to find out how to avoid memory loss, even if the person already shows signs of memory problems.	○	○	○	○	○
*find a doctor or medical practice that might appeal to the person's interests or preferences.	○	○	○	○	○
*call a dementia or Alzheimer's helpline for assistance.	○	○	○	○	○
*find out if there are options for house calls by health professionals who specialise in dementia.	○	○	○	○	○

Helping the confused older person

***73. If the person refuses to get help and their health or safety is at risk, the helper should get aged care assessment services involved.**

Essential	Important	Don't know/Depends	Unimportant	Should not be included
○	○	○	○	○

74. Do you have any comments on the above statements? Is there anything you would like to add? Please write your suggestions in the box provided.

Seeking help (continued)

This section asks you what information the helper should know about when and how the helper can assist the person to get professional help. Please rate how important (from 'essential' to 'should not be included') you think it is that each statement be included in the guidelines.

When providing first aid to the confused person, the helper will need to use their judgement because a person's decline in cognitive ability may be very mild or more severe. Please keep this in mind when rating the following items.

Please also keep the **definitions** in mind when rating the statements.

Preparing to see the doctor

***75. Before the person attends the initial appointment with the doctor, the helper should prepare by talking to family members about their individual concerns.**

Essential	Important	Don't know/Depends	Unimportant	Should not be included
○	○	○	○	○

***76. Before the person attends the initial appointment with the doctor, the helper should prepare by thinking about what information would be useful at the appointment, e.g. medical and family history, a list of changes you have noticed in the person, questions for the doctor.**

Essential	Important	Don't know/Depends	Unimportant	Should not be included
○	○	○	○	○

***77. If the helper attends a doctor's appointment with the person, the helper should directly ask the doctor for a referral to a specialist clinic or social services.**

Essential	Important	Don't know/Depends	Unimportant	Should not be included
○	○	○	○	○

***78. If the helper attends the doctor's appointment with the person, the helper should ask the person's permission to be given confidential information.**

Essential	Important	Don't know/Depends	Unimportant	Should not be included
◯	◯	◯	◯	◯

Other supports

***79. The helper should find out about support groups for the person with early stage dementia and their family and friends.**

Essential	Important	Don't know/Depends	Unimportant	Should not be included
◯	◯	◯	◯	◯

***80. The helper should find out about organisations that provide resources or services to people with dementia and their carers.**

Essential	Important	Don't know/Depends	Unimportant	Should not be included
◯	◯	◯	◯	◯

***81. If the person is reluctant to accept community health services, the helper should ask the doctor to persuade the person to do so.**

Essential	Important	Don't know/Depends	Unimportant	Should not be included
◯	◯	◯	◯	◯

82. Do you have any comments on the above statements? Is there anything you would like to add? Please write your suggestions in the box provided.

Encountering someone who is wandering

This section asks you what information the helper should know in order to assist a person who is wandering.

Wandering *is a dementia-related behaviour that sees a disoriented individual move about, sometimes with repetitive pacing or lapping in one area, and other times leaving their usual environment. A wandering person may become lost, leave a safe environment or intrude in inappropriate places.*

Please rate how important (from 'essential' to 'should not be included') you think it is that each statement be included in the guidelines.

Helping the confused older person

When providing first aid to the confused person, the helper will need to use their judgement because a person's decline in cognitive ability may be very mild or more severe. Please keep this in mind when rating the following items.

Please also keep the **definitions** in mind when rating the statements.

***83. The helper should be aware of any ID systems in their local area for identifying and helping people who are confused and lost, e.g. Australia (Safe Return Home), Canada (Safely Home), United Kingdom (Medic Alert), United States (Safe Return).**

Essential	Important	Don't know/Depends	Unimportant	Should not be included
◯	◯	◯	◯	◯

***84. The helper should be aware of local organisations that provide identification cards for people who wander.**

Essential	Important	Don't know/Depends	Unimportant	Should not be included
◯	◯	◯	◯	◯

***85. The helper should not assume that a person who appears to be wandering is necessarily confused, e.g. the person may want exercise.**

Essential	Important	Don't know/Depends	Unimportant	Should not be included
◯	◯	◯	◯	◯

***86. The helper should be aware of signs that can help them recognise that a person is lost and needs help, e.g. inappropriate clothing, an unsteady gait, unsafe or inappropriate behaviour.**

Essential	Important	Don't know/Depends	Unimportant	Should not be included
◯	◯	◯	◯	◯

***87. The helper should be aware that the person who is wandering may become frightened which could further reduce their ability to cope.**

Essential	Important	Don't know/Depends	Unimportant	Should not be included
◯	◯	◯	◯	◯

***88. The helper should be aware that the person who is wandering may be doing so for a variety of reasons, e.g. they've set off to go somewhere and forgotten where it was they were going, searching for a place from their past, believe that they have a job to do or they are bored.**

Essential	Important	Don't know/Depends	Unimportant	Should not be included
◯	◯	◯	◯	◯

***89. If the helper encounters a person who is wandering, the helper should:**

	Essential	Important	Don't know/ depends	Unimportant	Should not be included
*always introduce themselves to the person and offer help.	◯	◯	◯	◯	◯
*adopt a caring attitude and an approach that communicates warmth and respect to the person because this will confirm that the helper is not a threat and they have the person's best interests at heart.	◯	◯	◯	◯	◯
*ask the person if they can contact a family member or friend.	◯	◯	◯	◯	◯
*advise the person if the helper plans to contact the person's family member or a friend.	◯	◯	◯	◯	◯
*check if the person has any identification on them that will provide the helper with useful information.	◯	◯	◯	◯	◯
*check whether the person is injured because they may be unable to communicate that information effectively.	◯	◯	◯	◯	◯
*keep in mind that the person may have health problems affecting their movement and orientation, e.g. moving is painful or their eyesight is poor.	◯	◯	◯	◯	◯
*try to understand the person's perspective about why they are wandering, because this may assist the helper to respond in an appropriate way.	◯	◯	◯	◯	◯
*not be afraid to try any approach that may help them connect with the person and gain their trust.	◯	◯	◯	◯	◯
*see if the person has any needs that they could help them meet, e.g. they may be thirsty, hungry or need to go to the toilet.	◯	◯	◯	◯	◯

*be aware that the person may have impaired judgement regarding their own safety.

***90. In a situation where the helper encounters someone confused or wandering and knows where they live, the helper should quietly join the person and, in a friendly calm manner, engage in light conversation, e.g. asking the person, "Where are you going?" or "How is your day going?" After a short while, the helper should suggest something to change the direction of the walk, either sitting down for a moment or making a turn.**

Essential	Important	Don't know/Depends	Unimportant	Should not be included
○	○	○	○	○

***91. If the helper encounters a person who is wandering, and the helper cannot find out any of the individual's emergency contact information, the helper should suggest that they together call the police for help.**

Essential	Important	Don't know/Depends	Unimportant	Should not be included
○	○	○	○	○

***92. If the helper encounters a person who is wandering and looks scared or anxious, the helper should engage the person in conversation and attempt to calm them down and gain their trust.**

Essential	Important	Don't know/Depends	Unimportant	Should not be included
○	○	○	○	○

93. Do you have any comments on the above statements? Is there anything you would like to add? Please write your suggestions in the box provided.

Communication

This section asks you what information the helper should know in order to communicate effectively with a confused person. Please rate how important (from 'essential' to 'should not be included') you think it is that each statement be included in the guidelines.

Helping the confused older person

When providing first aid to the confused person, the helper will need to use their judgement because a person's decline in cognitive ability may be very mild or more severe. Please keep this in mind when rating the following items.

Please also keep the **definitions** in mind when rating the statements.

Creating a suitable environment for good communication

*94. The helper should be aware that busy, cluttered or noisy places may cause distress for the person and may make communication more difficult.

Essential	Important	Don't know/Depends	Unimportant	Should not be included
◯	◯	◯	◯	◯

*95. To gain the complete attention of the person, the helper should eliminate or reduce distracting noises, such as television, music or other people's voices.

Essential	Important	Don't know/Depends	Unimportant	Should not be included
◯	◯	◯	◯	◯

*96. The helper should be aware that the person may misunderstand aspects of their environment and this may increase confusion or frustration, e.g. mirrors might make the person think there is somebody else in the room.

Essential	Important	Don't know/Depends	Unimportant	Should not be included
◯	◯	◯	◯	◯

*97. The helper should be aware that an unfamiliar environment is more likely to increase the person's confusion.

Essential	Important	Don't know/Depends	Unimportant	Should not be included
◯	◯	◯	◯	◯

*98. To gain the complete attention of the person, the helper should ensure that lights are bright but not glaring.

Essential	Important	Don't know/Depends	Unimportant	Should not be included
◯	◯	◯	◯	◯

How to approach the person for a discussion

*99. When the helper approaches the person, they should be prepared to give the person their full attention.

Essential	Important	Don't know/Depends	Unimportant	Should not be included
◯	◯	◯	◯	◯

Helping the confused older person

***100. The helper should approach the person slowly and from the front so that they can be easily seen and the person is not startled.**

Essential	Important	Don't know/Depends	Unimportant	Should not be included
○	○	○	○	○

***101. The helper should avoid sudden movements so that the person is not startled.**

Essential	Important	Don't know/Depends	Unimportant	Should not be included
○	○	○	○	○

102. Do you have any comments on the above statements? Is there anything you would like to add? Please write your suggestions in the box provided.

Communication (continued)

This section asks you what information the helper should know in order to communicate effectively with a confused person. Please rate how important (from 'essential' to 'should not be included') you think it is that each statement be included in the guidelines.

When providing first aid to the confused person, the helper will need to use their judgement because a person's decline in cognitive ability may be very mild or more severe. Please keep this in mind when rating the following items.

Please also keep the **definitions** in mind when rating the statements.

Getting and retaining attention

***103. Before starting a conversation with the person, the helper should ensure they have the person's attention by approaching them slowly and giving them time to focus.**

Essential	Important	Don't know/Depends	Unimportant	Should not be included
○	○	○	○	○

***104. If the person does not appear to notice the helper, the helper should position themselves so the person can look down at them, e.g. if the person is seated the helper could crouch down in front of their chair.**

Essential	Important	Don't know/Depends	Unimportant	Should not be included
○	○	○	○	○

Helping the confused older person

***105. The helper should try to be at eye level when talking to the person.**

Essential	Important	Don't know/Depends	Unimportant	Should not be included
◯	◯	◯	◯	◯

***106. In order to make the person feel comfortable, the helper should be seated at the same level as the person or lower.**

Essential	Important	Don't know/Depends	Unimportant	Should not be included
◯	◯	◯	◯	◯

***107. To gain the person's attention, the helper should touch the person's arm or hand gently, whilst saying their name several times.**

Essential	Important	Don't know/Depends	Unimportant	Should not be included
◯	◯	◯	◯	◯

***108. The helper should not interrupt the person if they are in the middle of a task or a conversation, as this may confuse them.**

Essential	Important	Don't know/Depends	Unimportant	Should not be included
◯	◯	◯	◯	◯

***109. The helper should set the tone for an adult interaction with the person, e.g. by offering a handshake.**

Essential	Important	Don't know/Depends	Unimportant	Should not be included
◯	◯	◯	◯	◯

***110. The helper should establish friendly eye contact when speaking to the person to assure them that they have the helper's full attention.**

Essential	Important	Don't know/Depends	Unimportant	Should not be included
◯	◯	◯	◯	◯

***111. The helper should not loom over the person or talk loudly, because this might be interpreted as hostile or threatening.**

Essential	Important	Don't know/Depends	Unimportant	Should not be included
◯	◯	◯	◯	◯

Helping the confused older person

***112. The helper should avoid standing too close to the person whilst talking as this can be intimidating.**

Essential	Important	Don't know/Depends	Unimportant	Should not be included
◯	◯	◯	◯	◯

***113. If others are present in the discussion, the helper should speak directly to the person and avoid excluding them from conversation.**

Essential	Important	Don't know/Depends	Unimportant	Should not be included
◯	◯	◯	◯	◯

***114. While talking to the person, the helper should stay still and ensure that the person can see the helper's face and gestures to make it easier for the person to follow the conversation and keep the person's attention.**

Essential	Important	Don't know/Depends	Unimportant	Should not be included
◯	◯	◯	◯	◯

***115. If the helper cannot get the person's attention, they should wait a few minutes and try again.**

Essential	Important	Don't know/Depends	Unimportant	Should not be included
◯	◯	◯	◯	◯

116. Do you have any comments on the above statements? Is there anything you would like to add? Please write your suggestions in the box provided.

Communication (continued)

This section asks you what information the helper should know in order to communicate effectively with a confused person. Please rate how important (from 'essential' to 'should not be included') you think it is that each statement be included in the guidelines.

When providing first aid to the confused person, the helper will need to use their judgement because a person's decline in cognitive ability may be very mild or more severe. Please keep this in mind when rating the following items.

Please also keep the **definitions** in mind when rating the statements.

Where the person doesn't consistently recognise the people in their life

Helping the confused older person

***117. The helper should be aware that it may take time for the person to recognise the helper as someone they already know and trust.**

Essential	Important	Don't know/Depends	Unimportant	Should not be included
◯	◯	◯	◯	◯

***118. The helper should introduce themselves when approaching the person, and not assume the person knows who they are.**

Essential	Important	Don't know/Depends	Unimportant	Should not be included
◯	◯	◯	◯	◯

***119. The helper should reintroduce themselves each time they approach the person because the person may forget the names of people and their relationships to them.**

Essential	Important	Don't know/Depends	Unimportant	Should not be included
◯	◯	◯	◯	◯

***120. The helper should use orienting names whenever possible, e.g. "Your son Jack".**

Essential	Important	Don't know/Depends	Unimportant	Should not be included
◯	◯	◯	◯	◯

Where the person is sometimes confused about time and place

***121. The helper should begin talking to the person by providing orienting information, e.g. they should be told where they are and why, who they are with and the time of day.**

Essential	Important	Don't know/Depends	Unimportant	Should not be included
◯	◯	◯	◯	◯

***122. Because the person may not be oriented to time, the helper should adjust their communication to refer to daily events rather than dates or times, e.g. instead of saying, "John will be here at two o'clock", say "John will be here after lunch".**

Essential	Important	Don't know/Depends	Unimportant	Should not be included
◯	◯	◯	◯	◯

123. Do you have any comments on the above statements? Is there anything you would like to add? Please write your suggestions in the box provided.

Communication (continued)

This section asks you what information the helper should know in order to communicate effectively with a confused person. Please rate how important (from 'essential' to 'should not be included') you think it is that each statement be included in the guidelines.

When providing first aid to the confused person, the helper will need to use their judgement because a person's decline in cognitive ability may be very mild or more severe. Please keep this in mind when rating the following items.

Please also keep the **definitions** in mind when rating the statements.

Manner of communication

***124. The helper should treat the person with respect and not talk down to them.**

Essential	Important	Don't know/Depends	Unimportant	Should not be included
○	○	○	○	○

***125. The helper should talk to the person in a warm, easy-going, pleasant manner.**

Essential	Important	Don't know/Depends	Unimportant	Should not be included
○	○	○	○	○

***126. If the helper feels they have done something wrong when communicating with the person (e.g. acted impatiently) then they should apologise to demonstrate their respect.**

Essential	Important	Don't know/Depends	Unimportant	Should not be included
○	○	○	○	○

***127. The helper should be positive, optimistic and reassuring, e.g. by using expressions such as, "Everything will be okay." "Don't worry." "We're doing great." "We're going to see this through." "I'm here to help you."**

Essential	Important	Don't know/Depends	Unimportant	Should not be included
○	○	○	○	○

***128. The helper should avoid negativity when speaking to the person, e.g. the helper should limit the number of 'don'ts' and avoid giving harsh orders.**

Essential	Important	Don't know/Depends	Unimportant	Should not be included
○	○	○	○	○

***129. The helper should offer options instead of commands in order to give the person a greater sense of control over their life.**

Essential	Important	Don't know/Depends	Unimportant	Should not be included
○	○	○	○	○

***130. The helper should be aware that the person may not be able to attend to a discussion or task for very long and they should look for signs of frustration.**

Essential	Important	Don't know/Depends	Unimportant	Should not be included
○	○	○	○	○

***131. The helper should stay still whilst talking to the person as this will make it easier for the person to follow what you are saying.**

Essential	Important	Don't know/Depends	Unimportant	Should not be included
○	○	○	○	○

***132. The helper should be aware that it may take time for the person to sense that the helper is friendly.**

Essential	Important	Don't know/Depends	Unimportant	Should not be included
○	○	○	○	○

***133. The helper should try and connect with the person by drawing upon the person's remaining social skills, e.g. "Hello, how are you?" and "Pleased to see you today."**

Essential	Important	Don't know/Depends	Unimportant	Should not be included
○	○	○	○	○

Helping the confused older person

***134. The helper should explain to the person each step of the way what they are doing in a soft, reassuring way, e.g. "I am going to help you sit down now. Then we can talk."**

Essential	Important	Don't know/Depends	Unimportant	Should not be included
○	○	○	○	○

135. Do you have any comments on the above statements? Is there anything you would like to add? Please write your suggestions in the box provided.

Communication (continued)

This section asks you what information the helper should know in order to communicate effectively with a confused person. Please rate how important (from 'essential' to 'should not be included') you think it is that each statement be included in the guidelines.

When providing first aid to the confused person, the helper will need to use their judgement because a person's decline in cognitive ability may be very mild or more severe. Please keep this in mind when rating the following items.

Please also keep the **definitions** in mind when rating the statements.

Language and conversation content

*136. When talking to a person the helper should:

	Essential	Important	Don't know/ depends	Unimportant	Should not be included
*use short words.	◯	◯	◯	◯	◯
*use short sentences.	◯	◯	◯	◯	◯
*use concrete words (words you can picture), rather than abstract words that may be difficult to understand.	◯	◯	◯	◯	◯
*focus on one main idea at a time.	◯	◯	◯	◯	◯
*stress words that are most important in a sentence, e.g. 'Here is your *coffee*.'	◯	◯	◯	◯	◯
*pause between sentences to allow time for the information to be understood by the person.	◯	◯	◯	◯	◯
*try to use positively framed instructions, such as "stay here" rather than "don't go away".	◯	◯	◯	◯	◯
*avoid using idioms, metaphors, slang and other speech variations because these may be difficult for the person to interpret.	◯	◯	◯	◯	◯
*avoid using expressions that can be taken too literally, e.g. "shake a leg".	◯	◯	◯	◯	◯
*avoid discussing too many things at once.	◯	◯	◯	◯	◯
*avoid using pronouns, e.g. instead of saying "Here it is," say, "Here is your hat."	◯	◯	◯	◯	◯
*avoid using third person pronouns (e.g. "he" or "she") and instead identify people by their actual names.	◯	◯	◯	◯	◯

*137. After the person responds to a question, the helper should repeat what the person said to show them they have understood.

Essential	Important	Don't know/Depends	Unimportant	Should not be included
◯	◯	◯	◯	◯

*138. The helper should accept that pauses in the conversation do not need to be filled with words.

Essential	Important	Don't know/Depends	Unimportant	Should not be included
◯	◯	◯	◯	◯

Helping the confused older person

139. Do you have any comments on the above statements? Is there anything you would like to add? Please write your suggestions in the box provided.

Communication (continued)

This section asks you what information the helper should know in order to communicate effectively with a confused person. Please rate how important (from 'essential' to 'should not be included') you think it is that each statement be included in the guidelines.

When providing first aid to the confused person, the helper will need to use their judgement because a person's decline in cognitive ability may be very mild or more severe. Please keep this in mind when rating the following items.

Please also keep the **definitions** in mind when rating the statements.

Asking the person questions

***140. The helper should ask simple questions that require the choice of a 'yes' or 'no' answer, rather than open-ended questions.**

Essential	Important	Don't know/Depends	Unimportant	Should not be included
○	○	○	○	○

***141. The helper should be aware that the person may respond well to open-ended questions and so the helper should not avoid using them.**

Essential	Important	Don't know/Depends	Unimportant	Should not be included
○	○	○	○	○

***142. The helper should use either open- or closed-ended questions depending on the purpose of the conversation with the person, e.g. closed-ended questions may be useful for helping the person in an everyday task, whereas open-ended questions may encourage conversation with the person about feelings.**

Essential	Important	Don't know/Depends	Unimportant	Should not be included
○	○	○	○	○

***143. The helper should not use phrases such as, "do you remember?", "don't you remember?" and "have you forgotten?", because it may upset the person and is unlikely to be helpful.**

Essential	Important	Don't know/Depends	Unimportant	Should not be included
◯	◯	◯	◯	◯

***144. The helper should only ask the person one question at a time.**

Essential	Important	Don't know/Depends	Unimportant	Should not be included
◯	◯	◯	◯	◯

***145. When offering the person a choice, the helper should list the available options so that the person can use the information to answer, e.g. "Would you like tea or coffee?" rather than "What would you like to drink?"**

Essential	Important	Don't know/Depends	Unimportant	Should not be included
◯	◯	◯	◯	◯

***146. The helper should use statements over questions, where possible, because they do not call upon the person to make decisions.**

Essential	Important	Don't know/Depends	Unimportant	Should not be included
◯	◯	◯	◯	◯

***147. The helper should avoid 'why' questions which may put the person on the defensive, e.g. "Why did you do that?"**

Essential	Important	Don't know/Depends	Unimportant	Should not be included
◯	◯	◯	◯	◯

***148. The helper should not ask questions that require a lot of thought or memory, e.g. don't ask, "What did you do today?" Instead, shape the question to address current feelings: "Are you having a good day?".**

Essential	Important	Don't know/Depends	Unimportant	Should not be included
◯	◯	◯	◯	◯

***149. The helper should be aware that the person's answers to questions may not reflect what they meant to say.**

Essential	Important	Don't know/Depends	Unimportant	Should not be included
○	○	○	○	○

***150. If the person doesn't respond to a question, the helper should wait a moment and repeat the question using exactly the same words.**

Essential	Important	Don't know/Depends	Unimportant	Should not be included
○	○	○	○	○

***151. If the helper has repeated a question using exactly the same words and the person still does not respond, the helper should try to get the message across in a different way.**

Essential	Important	Don't know/Depends	Unimportant	Should not be included
○	○	○	○	○

***152. If the person doesn't directly answer a question, or the answer seems out of context, the helper should show that they have heard them and encourage them to say more about their answer.**

Essential	Important	Don't know/Depends	Unimportant	Should not be included
○	○	○	○	○

***153. The helper should avoid testing the person's memory because this can be embarrassing and hurtful.**

Essential	Important	Don't know/Depends	Unimportant	Should not be included
○	○	○	○	○

***154. The helper should avoid quizzing the person and instead help them by providing appropriate information for a situation, e.g. when introducing people, you might say, "Here's your nephew John and his wife Sharon." instead of asking "Do you remember everyone's name?"**

Essential	Important	Don't know/Depends	Unimportant	Should not be included
○	○	○	○	○

Helping the confused older person

***155. The helper should ask questions that call for short answers, as this may make the person feel successful instead of embarrassed and frustrated over their inability to form and keep in mind a lengthy answer.**

Essential	Important	Don't know/Depends	Unimportant	Should not be included
○	○	○	○	○

156. Do you have any comments on the above statements? Is there anything you would like to add? Please write your suggestions in the box provided.

Communication (continued)

This section asks you what information the helper should know in order to communicate effectively with a confused person. Please rate how important (from 'essential' to 'should not be included') you think it is that each statement be included in the guidelines.

When providing first aid to the confused person, the helper will need to use their judgement because a person's decline in cognitive ability may be very mild or more severe. Please keep this in mind when rating the following items.

Please also keep the **definitions** in mind when rating the statements.

Encouraging communication and comprehension

***157. The helper should aid the person's comprehension by introducing a topic at the start of a discussion and then gradually fill in the specifics. This will help the person link the content and the details.**

Essential	Important	Don't know/Depends	Unimportant	Should not be included
○	○	○	○	○

***158. The helper should initially assume that the person understands what the helper is saying.**

Essential	Important	Don't know/Depends	Unimportant	Should not be included
○	○	○	○	○

***159. The helper needs to make sure that what they are saying is understood by the person and should not assume that the person understands because they are nodding or giving a superficial response.**

Essential	Important	Don't know/Depends	Unimportant	Should not be included
○	○	○	○	○

***160. The helper should encourage the person to continue to express themselves, even if they are having trouble making themselves understood.**

Essential	Important	Don't know/Depends	Unimportant	Should not be included
○	○	○	○	○

***161. The helper should acknowledge the feelings the person is experiencing.**

Essential	Important	Don't know/Depends	Unimportant	Should not be included
○	○	○	○	○

***162. The helper should provide validation and acknowledgement to the person by reflecting back what the person has said.**

Essential	Important	Don't know/Depends	Unimportant	Should not be included
○	○	○	○	○

***163. The helper should provide validation and acknowledgement to the person by listening with interest and nodding their head appropriately.**

Essential	Important	Don't know/Depends	Unimportant	Should not be included
○	○	○	○	○

***164. The helper should validate the person's beliefs or feelings, no matter how outlandish these might be, because this might help the person stay calm or feel good.**

Essential	Important	Don't know/Depends	Unimportant	Should not be included
○	○	○	○	○

***165. The helper should be aware that humour may help them communicate effectively with the person.**

Essential	Important	Don't know/Depends	Unimportant	Should not be included
○	○	○	○	○

***166. The helper should try using visual cues to help the person understand what they mean, e.g. point towards the sandwich if you are asking if they would like one.**

Essential	Important	Don't know/Depends	Unimportant	Should not be included
○	○	○	○	○

***167. The helper should keep conversation with the person brief, as it is very easy for the person to lose the thread of the discussion.**

Essential	Important	Don't know/Depends	Unimportant	Should not be included
◯	◯	◯	◯	◯

***168. The helper should speak at their normal pace, because speaking slower means taking longer to say what needs to be said, making it harder for the person to follow.**

Essential	Important	Don't know/Depends	Unimportant	Should not be included
◯	◯	◯	◯	◯

***169. The helper should do their best to keep a conversation on track because the person may tend to get lost in a flurry of words and thoughts.**

Essential	Important	Don't know/Depends	Unimportant	Should not be included
◯	◯	◯	◯	◯

***170. If the person loses their train of thought, the helper should reassure the person by using expressions such as "Everything will be okay," "Don't worry" and "You're doing well."**

Essential	Important	Don't know/Depends	Unimportant	Should not be included
◯	◯	◯	◯	◯

***171. The helper should continue sharing their day-to-day thoughts and feelings with the person, as it lets the person know that they are valued.**

Essential	Important	Don't know/Depends	Unimportant	Should not be included
◯	◯	◯	◯	◯

Communicating with the person when others are present

***172. The helper should try and keep conversations with the person as one-on-one whenever possible because even small groups may make the person confused and anxious.**

Essential	Important	Don't know/Depends	Unimportant	Should not be included
◯	◯	◯	◯	◯

Helping the confused older person

***173. In a group situation where a one-on-one conversation with the person is not possible, the helper should try to ensure that only one person speaks at a time.**

Essential	Important	Don't know/Depends	Unimportant	Should not be included
○	○	○	○	○

***174. When someone else is trying to communicate with the person, the helper should let the person respond and not answer questions for them, e.g. at medical appointments.**

Essential	Important	Don't know/Depends	Unimportant	Should not be included
○	○	○	○	○

***175. The helper should remind others that they should avoid talking about the person when the person is within hearing range.**

Essential	Important	Don't know/Depends	Unimportant	Should not be included
○	○	○	○	○

***176. In a group situation, the helper should show others how to include the person by addressing the person directly and through body language.**

Essential	Important	Don't know/Depends	Unimportant	Should not be included
○	○	○	○	○

***177. If the helper observes someone using baby-talk with the person, the helper should say, "I'm sorry, but (name) relates best when you talk to them in a normal adult manner."**

Essential	Important	Don't know/Depends	Unimportant	Should not be included
○	○	○	○	○

178. Do you have any comments on the above statements? Is there anything you would like to add? Please write your suggestions in the box provided.

Non-verbal communication

Helping the confused older person

This section asks you what information the helper should know in order to communicate effectively with a confused person. Please rate how important (from 'essential' to 'should not be included') you think it is that each statement be included in the guidelines.

When providing first aid to the confused person, the helper will need to use their judgement because a person's decline in cognitive ability may be very mild or more severe. Please keep this in mind when rating the following items.

Please also keep the **definitions** in mind when rating the statements.

Awareness of body language

***179. The helper should be aware that visual cues, props, gestures, and other forms of body language are also helpful in reinforcing verbal messages.**

Essential	Important	Don't know/Depends	Unimportant	Should not be included
○	○	○	○	○

***180. The helper should be aware that communicating with the person using body language may be more effective than spoken words.**

Essential	Important	Don't know/Depends	Unimportant	Should not be included
○	○	○	○	○

***181. The helper should observe the person's tone of voice and body language to help understand how the person is feeling.**

Essential	Important	Don't know/Depends	Unimportant	Should not be included
○	○	○	○	○

Understanding the person's body language

***182. The helper should look for and respond to the physical and nonverbal cues that may indicate the person's needs or feelings.**

Essential	Important	Don't know/Depends	Unimportant	Should not be included
○	○	○	○	○

***183. The helper should be aware that the person who is unable to communicate may use nonverbal cues to signal a need, e.g. the person who appears anxious or agitated may be in pain, need to go to the toilet or be troubled by something going on around them.**

Essential	Important	Don't know/Depends	Unimportant	Should not be included
○	○	○	○	○

***184. The helper should observe the person's breathing to gauge how they are feeling, e.g. faster breathing may indicate distress, while slower breathing may show they are calm.**

Essential	Important	Don't know/Depends	Unimportant	Should not be included
◯	◯	◯	◯	◯

***185. The helper should be aware that if the person breaks off eye contact, this may mean they have not understood what the helper is saying, but do not want to admit it.**

Essential	Important	Don't know/Depends	Unimportant	Should not be included
◯	◯	◯	◯	◯

The helper's body language

***186. The helper should be aware of the facial expressions they are conveying to the person.**

Essential	Important	Don't know/Depends	Unimportant	Should not be included
◯	◯	◯	◯	◯

***187. The helper should try to keep their facial expressions and gestures positive.**

Essential	Important	Don't know/Depends	Unimportant	Should not be included
◯	◯	◯	◯	◯

***188. The helper should use facial expressions to communicate meanings to the person because these may be more readily understood.**

Essential	Important	Don't know/Depends	Unimportant	Should not be included
◯	◯	◯	◯	◯

***189. The helper should be aware that their behaviour may send a message to the person, e.g. their behaviour may indicate that they are frustrated with the person.**

Essential	Important	Don't know/Depends	Unimportant	Should not be included
◯	◯	◯	◯	◯

Helping the confused older person

***190. The helper should use a non-threatening tone of voice and body language in order to gain the person's confidence in the helper's ability to assist.**

Essential	Important	Don't know/Depends	Unimportant	Should not be included
○	○	○	○	○

***191. The helper should try to make sure that their non-verbal communication matches their verbal communication.**

Essential	Important	Don't know/Depends	Unimportant	Should not be included
○	○	○	○	○

192. Do you have any comments on the above statements? Is there anything you would like to add? Please write your suggestions in the box provided.

Non-verbal communication (continued)

This section asks you what information the helper should know in order to communicate effectively with a confused person. Please rate how important (from 'essential' to 'should not be included') you think it is that each statement be included in the guidelines.

When providing first aid to the confused person, the helper will need to use their judgement because a person's decline in cognitive ability may be very mild or more severe. Please keep this in mind when rating the following items.

Please also keep the **definitions** in mind when rating the statements.

Using touch to communicate

***193. The helper should be aware that a gentle touch of the arm or hand can communicate to the person that the helper is interested and really cares.**

Essential	Important	Don't know/Depends	Unimportant	Should not be included
○	○	○	○	○

***194. The helper should be aware that a simple touch of the hand or shoulder may calm the person who is fearful, angry or upset.**

Essential	Important	Don't know/Depends	Unimportant	Should not be included
○	○	○	○	○

***195. The helper should be aware that if they have a close relationship with the person, touch can be a very powerful way of connecting with them.**

Essential	Important	Don't know/Depends	Unimportant	Should not be included
○	○	○	○	○

***196. The helper should use touch to comfort and reassure the person.**

Essential	Important	Don't know/Depends	Unimportant	Should not be included
○	○	○	○	○

***197. Before using touch to provide comfort, the helper should ask the person if it is alright to touch them.**

Essential	Important	Don't know/Depends	Unimportant	Should not be included
○	○	○	○	○

***198. The helper should pay attention to non-verbal cues that the person does not want to be touched.**

Essential	Important	Don't know/Depends	Unimportant	Should not be included
○	○	○	○	○

Tone and pitch

***199. The helper should be aware that their tone of voice is as important as the words they say to the person.**

Essential	Important	Don't know/Depends	Unimportant	Should not be included
○	○	○	○	○

***200. The helper should be aware that their tone of voice or displays of emotion may be mirrored by the person, e.g. the helper's anxious tone may trigger anxiety in the person.**

Essential	Important	Don't know/Depends	Unimportant	Should not be included
○	○	○	○	○

***201. The helper should try to talk in a low pitch to the person because people lose their ability to hear higher pitches as they age.**

Essential	Important	Don't know/Depends	Unimportant	Should not be included
○	○	○	○	○

202. Do you have any comments on the above statements? Is there anything you would like to add? Please write your suggestions in the box provided.

Challenges you may encounter during communication

This section asks you what information the helper should know in order to respond to challenges that may occur whilst communicating with a confused person. Please rate how important (from 'essential' to 'should not be included') you think it is that each statement be included in the guidelines.

When providing first aid to the confused person, the helper will need to use their judgement because a person's decline in cognitive ability may be very mild or more severe. Please keep this in mind when rating the following items.

Please also keep the **definitions** in mind when rating the statements.

When the helper doesn't understand what the person is saying

***203. If the helper does not understand what the person is trying to say, the helper should ask the person to point, gesture or touch.**

Essential	Important	Don't know/Depends	Unimportant	Should not be included
○	○	○	○	○

***204. If the helper does not understand what the person is saying then the helper should apologise and ask the person to repeat it.**

Essential	Important	Don't know/Depends	Unimportant	Should not be included
○	○	○	○	○

***205. When communication is difficult, the helper should not give up trying to understand the person.**

Essential	Important	Don't know/Depends	Unimportant	Should not be included
○	○	○	○	○

When the person can't find the right words

***206. The helper should be aware that the person may need to 'talk around' a topic before finding the right word or phrase.**

Essential	Important	Don't know/Depends	Unimportant	Should not be included
○	○	○	○	○

***207. The helper should be aware that sometimes the best thing they can do is remain silent and let the person find their own way to cope with difficulties in conversation, e.g. finding a word to use.**

Essential	Important	Don't know/Depends	Unimportant	Should not be included
◯	◯	◯	◯	◯

***208. The helper should not finish the person's sentences for them or 'fill in the blanks' too quickly.**

Essential	Important	Don't know/Depends	Unimportant	Should not be included
◯	◯	◯	◯	◯

***209. If the person is unable to find the right word, but the helper has understood what they are trying to say, the helper should not correct the person as this may cause unnecessary frustration.**

Essential	Important	Don't know/Depends	Unimportant	Should not be included
◯	◯	◯	◯	◯

***210. The helper should avoid pointing out any word errors made by the person as this can be discouraging for someone with memory loss.**

Essential	Important	Don't know/Depends	Unimportant	Should not be included
◯	◯	◯	◯	◯

***211. Although the helper should help the person when they are having difficulty finding the right word or phrase, the helper should avoid the temptation to interrupt or speak for the person.**

Essential	Important	Don't know/Depends	Unimportant	Should not be included
◯	◯	◯	◯	◯

***212. If the person is trying to respond verbally but is becoming frustrated, the helper should help out by finishing a sentence or supplying a word.**

Essential	Important	Don't know/Depends	Unimportant	Should not be included
◯	◯	◯	◯	◯

***213. Once the helper has given the person time to find the appropriate words and the words aren't forthcoming, the helper should gently suggest what the person might want to say.**

Essential	Important	Don't know/Depends	Unimportant	Should not be included
○	○	○	○	○

***214. The helper should help the person who cannot remember a word because the more time that is spent searching for the word, the more likely that the person will lose the thought altogether.**

Essential	Important	Don't know/Depends	Unimportant	Should not be included
○	○	○	○	○

***215. If the person's words are mixed up and seem nonsensical, the helper should smile and say, "I am having a problem understanding". This implies that the person is not the problem.**

Essential	Important	Don't know/Depends	Unimportant	Should not be included
○	○	○	○	○

***216. When the person's words aren't making sense, the helper should focus on the emotion that the person is trying to convey because this will help the person feel understood even if they cannot find the appropriate words.**

Essential	Important	Don't know/Depends	Unimportant	Should not be included
○	○	○	○	○

***217. If the person is having trouble expressing themselves, the helper should let them know it is alright and encourage them to continue to explain their thoughts.**

Essential	Important	Don't know/Depends	Unimportant	Should not be included
○	○	○	○	○

***218. If the person is having trouble expressing themselves and the helper suspects the person is in pain, the helper should point to or touch the area you think might be hurting and ask the person to nod if it hurts there.**

Essential	Important	Don't know/Depends	Unimportant	Should not be included
○	○	○	○	○

219. Do you have any comments on the above statements? Is there anything you would like to add? Please write your suggestions in the box provided.

Challenges you may encounter during communication (continued)

This section asks you what information the helper should know in order to respond to challenges that may occur whilst communicating with a confused person. Please rate how important (from 'essential' to 'should not be included') you think it is that each statement be included in the guidelines.

When providing first aid to the confused person, the helper will need to use their judgement because a person's decline in cognitive ability may be very mild or more severe. Please keep this in mind when rating the following items.

Please also keep the **definitions** in mind when rating the statements.

Problems with comprehension

***220. The helper should check that the person with communication problems is not being affected by something unrelated, e.g. their hearing aid may not be working or they are wearing the wrong glasses.**

Essential	Important	Don't know/Depends	Unimportant	Should not be included
○	○	○	○	○

***221. The helper should ask the person for feedback on whether the person has understood them and look for signs of understanding, e.g. the person's body language and facial expression.**

Essential	Important	Don't know/Depends	Unimportant	Should not be included
○	○	○	○	○

***222. If the person doesn't understand, the helper should resist the urge to talk louder as this will upset the person and make communication more difficult.**

Essential	Important	Don't know/Depends	Unimportant	Should not be included
○	○	○	○	○

***223. If the person is having trouble understanding, then the helper could try using written communication instead.**

Essential	Important	Don't know/Depends	Unimportant	Should not be included
○	○	○	○	○

Arguments

***224. The helper should avoid arguing with the person because this is likely to cause the person to become angry, anxious, frustrated or more confused.**

Essential	Important	Don't know/Depends	Unimportant	Should not be included
○	○	○	○	○

***225. Rather than asking questions that are likely to trigger immediate resistance, the helper should tell the person what is going to happen, e.g. rather than asking "Do you want to have your shower now?", the helper should say "It's time for your shower now".**

Essential	Important	Don't know/Depends	Unimportant	Should not be included
○	○	○	○	○

***226. If an argument develops, the helper should change the topic of conversation or begin a new activity.**

Essential	Important	Don't know/Depends	Unimportant	Should not be included
○	○	○	○	○

***227. If an argument develops, the helper should agree with the person and then try to change the subject because they are likely to forget afterwards.**

Essential	Important	Don't know/Depends	Unimportant	Should not be included
○	○	○	○	○

Silence

Helping the confused older person

***228. The helper should be aware that silence can be a way to connect with the person.**

Essential	Important	Don't know/Depends	Unimportant	Should not be included
○	○	○	○	○

***229. The helper should be aware that silence is not necessarily a negative thing.**

Essential	Important	Don't know/Depends	Unimportant	Should not be included
○	○	○	○	○

***230. The helper should be aware that they may have more of a problem with silence than the person does.**

Essential	Important	Don't know/Depends	Unimportant	Should not be included
○	○	○	○	○

***231. The helper should not automatically interpret the person's silence as anger or depression.**

Essential	Important	Don't know/Depends	Unimportant	Should not be included
○	○	○	○	○

***232. The helper should be aware that if the person does not talk much this does not mean that their thoughts and feelings are absent.**

Essential	Important	Don't know/Depends	Unimportant	Should not be included
○	○	○	○	○

233. Do you have any comments on the above statements? Is there anything you would like to add? Please write your suggestions in the box provided.

Challenges you may encounter during communication (continued)

This section asks you what information the helper should know in order to respond to challenges that may occur whilst communicating with a confused person. Please rate how important (from 'essential' to 'should not be included') you think it is that each statement be included in the guidelines.

When providing first aid to the confused person, the helper will need to use their judgement because a person's decline in cognitive ability may be very mild or more severe. Please keep this in mind when rating the following items.

Please also keep the **definitions** in mind when rating the statements.

Responding to incoherence, unresponsiveness or agitation

Helping the confused older person

***234. The helper should not blame the person for mistakes that arise from the person's lack of ability or their misunderstanding of the situation.**

Essential	Important	Don't know/Depends	Unimportant	Should not be included
◯	◯	◯	◯	◯

***235. If the person begins rambling, the helper should avoid looking away or acting distracted because the person will sense the lack of interest and this may close down communication.**

Essential	Important	Don't know/Depends	Unimportant	Should not be included
◯	◯	◯	◯	◯

***236. If the person starts talking incoherently or loses their train of thought, the helper should repeat the last coherent words, as this may help the person pick up the thread of conversation again.**

Essential	Important	Don't know/Depends	Unimportant	Should not be included
◯	◯	◯	◯	◯

***237. If the helper has to repeat what they have said because of the person's forgetfulness, the helper should avoid paraphrasing because this may confuse the person.**

Essential	Important	Don't know/Depends	Unimportant	Should not be included
◯	◯	◯	◯	◯

***238. The helper should paraphrase rather than exactly repeat what the person has said to them because this will help the person produce more coherent communication.**

Essential	Important	Don't know/Depends	Unimportant	Should not be included
◯	◯	◯	◯	◯

***239. If the person starts talking incoherently, the helper should phrase any understood words as a question as this might encourage a reply, e.g. "What were you saying about...?".**

Essential	Important	Don't know/Depends	Unimportant	Should not be included
◯	◯	◯	◯	◯

Helping the confused older person

***240. If the person is talking in a confused way, the helper should show the person they are listening by maintaining eye contact, smiling and talking in a gentle tone, as this will encourage the person to continue communicating.**

Essential	Important	Don't know/Depends	Unimportant	Should not be included
◯	◯	◯	◯	◯

***241. If the person does not respond, or if it looks like the person is not paying attention, the helper should try again after a break when the person may be more focused.**

Essential	Important	Don't know/Depends	Unimportant	Should not be included
◯	◯	◯	◯	◯

***242. If the person does not want to talk, turns away or says or gestures "No!" then the helper should try again with a new approach after a break.**

Essential	Important	Don't know/Depends	Unimportant	Should not be included
◯	◯	◯	◯	◯

***243. If the person does not want to talk, turns away or says or gestures "No!" then the helper should not force them to talk.**

Essential	Important	Don't know/Depends	Unimportant	Should not be included
◯	◯	◯	◯	◯

244. Do you have any comments on the above statements? Is there anything you would like to add? Please write your suggestions in the box provided.

Discussing sensitive issues and making decisions with the person

This section concerns conversations about sensitive issues which might include when to see a doctor about symptoms of dementia, stopping driving or the need for a change in level of care or living arrangements. In this section, 'a conversation' refers to a conversation about a sensitive issue. Please rate how important (from 'essential' to 'should not be included') you think it is that each statement be included in the guidelines.

When providing first aid to the confused person, the helper will need to use their judgement because a person's decline in cognitive ability may be very mild or more severe. Please keep this in mind when rating the following items.

Please also keep the **definitions** in mind when rating the statements.

How to discuss sensitive issues

***245. The helper should choose a place familiar to the person as a setting for a conversation because this is likely to help facilitate communication and minimise anxiety.**

Essential	Important	Don't know/Depends	Unimportant	Should not be included
○	○	○	○	○

***246. The helper should have a one-on-one conversation with the person in a quiet setting, as this will increase the chances of getting and maintaining the attention of the person.**

Essential	Important	Don't know/Depends	Unimportant	Should not be included
○	○	○	○	○

***247. If it is possible, the helper should wait until the person is relaxed and focused before trying to communicate with them, e.g. the person may be more tired or confused in the evening and more responsive after they have rested.**

Essential	Important	Don't know/Depends	Unimportant	Should not be included
○	○	○	○	○

***248. The helper should approach the person in a calm, gentle and non-judgmental manner because this sets the mood for the subsequent conversation.**

Essential	Important	Don't know/Depends	Unimportant	Should not be included
○	○	○	○	○

***249. The helper should begin the conversation with neutral topics to help build trust and help the person feel relaxed, e.g. talking about the weather, family.**

Essential	Important	Don't know/Depends	Unimportant	Should not be included
○	○	○	○	○

***250. When starting the conversation, the helper should state the purpose of the conversation.**

Essential	Important	Don't know/Depends	Unimportant	Should not be included
○	○	○	○	○

Helping the confused older person

***251. The helper should be aware that there is no need to be embarrassed at displays of negative emotion by the person (e.g. tearful, angry), because it is good for the person to express their emotions.**

Essential	Important	Don't know/Depends	Unimportant	Should not be included
○	○	○	○	○

***252. If the person shows negative emotions (e.g. tearful, angry), the helper should not attempt to stop or avoid this.**

Essential	Important	Don't know/Depends	Unimportant	Should not be included
○	○	○	○	○

***253. When discussing topics which evoke a strong emotional response from the person, the helper should not reject or dismiss what the person says about their feelings, e.g. 'Oh, you don't need to worry about that'.**

Essential	Important	Don't know/Depends	Unimportant	Should not be included
○	○	○	○	○

254. Do you have any comments on the above statements? Is there anything you would like to add? Please write your suggestions in the box provided.

Discussing sensitive issues and making decisions with the person (continued...

This section concerns conversations about sensitive issues which might include when to see a doctor about symptoms of dementia, stopping driving or the need for a change in level of care or living arrangements. In this section, 'a conversation' refers to a conversation about a sensitive issue. Please rate how important (from 'essential' to 'should not be included') you think it is that each statement be included in the guidelines.

When providing first aid to the confused person, the helper will need to use their judgement because a person's decline in cognitive ability may be very mild or more severe. Please keep this in mind when rating the following items.

Please also keep the **definitions** in mind when rating the statements.

Discussing the diagnosis

***255. The helper should be aware that people with dementia usually want to know the truth about their diagnosis and can cope with the implications.**

Essential	Important	Don't know/Depends	Unimportant	Should not be included
◯	◯	◯	◯	◯

***256. The helper should be aware that it is generally recommended that a person with dementia be told of their diagnosis, though a person has a right not to know their diagnosis if that is their clear and informed preference.**

Essential	Important	Don't know/Depends	Unimportant	Should not be included
◯	◯	◯	◯	◯

***257. The helper should be aware that the person may feel a sense of relief from knowing their diagnosis.**

Essential	Important	Don't know/Depends	Unimportant	Should not be included
◯	◯	◯	◯	◯

***258. The helper should be aware that knowing the diagnosis can enable the person to take an active role in planning for their future.**

Essential	Important	Don't know/Depends	Unimportant	Should not be included
◯	◯	◯	◯	◯

***259. If the person has not been told their diagnosis of dementia by a health professional, the helper should tell them.**

Essential	Important	Don't know/Depends	Unimportant	Should not be included
◯	◯	◯	◯	◯

***260. The helper should ensure that the person is told their diagnosis so they know why they are experiencing changes in functioning and can take steps to improve their quality of life.**

Essential	Important	Don't know/Depends	Unimportant	Should not be included
◯	◯	◯	◯	◯

***261. If the person has not been told their diagnosis of dementia by a health professional and they are denying that anything it wrong, then the helper should not tell the person about their diagnosis because it may distress them.**

Essential	Important	Don't know/Depends	Unimportant	Should not be included
◯	◯	◯	◯	◯

***262. If the person has not been told their diagnosis of dementia by a health professional and they are aware something is wrong and asking what is happening, the helper should tell the person about their diagnosis.**

Essential	Important	Don't know/Depends	Unimportant	Should not be included
◯	◯	◯	◯	◯

***263. The helper should be aware that the person may not acknowledge their diagnosis and this could be due to an inability to understand or a self-protective mechanism to help them cope.**

Essential	Important	Don't know/Depends	Unimportant	Should not be included
◯	◯	◯	◯	◯

***264. If the person does not acknowledge their diagnosis, the helper should not try to force the person to recognise it.**

Essential	Important	Don't know/Depends	Unimportant	Should not be included
◯	◯	◯	◯	◯

***265. The helper should choose how they explain to the person what is happening based on the person's ability to understand, e.g. A straightforward explanation versus a more gentle approach, "You have a memory problem".**

Essential	Important	Don't know/Depends	Unimportant	Should not be included
◯	◯	◯	◯	◯

***266. If needed, the helper should get advice from the person's health professionals on how to talk to the person about their diagnosis.**

Essential	Important	Don't know/Depends	Unimportant	Should not be included
◯	◯	◯	◯	◯

Helping the confused older person

***267. The helper should make sure that the person reaches some understanding about the diagnosis because they need to know that anyone caring for them will sometimes need a break.**

Essential	Important	Don't know/Depends	Unimportant	Should not be included
○	○	○	○	○

***268. If the person does not understand all that their diagnosis means or they deny it, the helper should accept their reaction and avoid further detailed explanations of the disease.**

Essential	Important	Don't know/Depends	Unimportant	Should not be included
○	○	○	○	○

***269. Whilst discussing the diagnosis, the helper should reassure the person and let them know that they will be supported and helped.**

Essential	Important	Don't know/Depends	Unimportant	Should not be included
○	○	○	○	○

***270. The helper should use the term 'memory loss' to discuss the diagnosis if the word 'dementia' or the disease name will upset the person.**

Essential	Important	Don't know/Depends	Unimportant	Should not be included
○	○	○	○	○

271. Do you have any comments on the above statements? Is there anything you would like to add? Please write your suggestions in the box provided.

Discussing sensitive issues and making decisions with the person (continued...

Helping the confused older person

This section concerns conversations about sensitive issues which might include when to see a doctor about symptoms of dementia, stopping driving or the need for a change in level of care or living arrangements. In this section, 'a conversation' refers to a conversation about a sensitive issue. Please rate how important (from ‘essential’ to ‘should not be included’) you think it is that each statement be included in the guidelines.

When providing first aid to the confused person, the helper will need to use their judgement because a person's decline in cognitive ability may be very mild or more severe. Please keep this in mind when rating the following items.

Please also keep the **definitions** in mind when rating the statements.

Discussions post-diagnosis

*272. The helper should remain open to the person's need to talk about their diagnosis, increased limitations and negative feelings as their dementia progresses.

Essential	Important	Don't know/Depends	Unimportant	Should not be included
○	○	○	○	○

*273. The helper should be aware that discussions at diagnosis can provide a basis for more detailed discussions later on, e.g. discussions about dementia progression, advice on topics that may be discussed later.

Essential	Important	Don't know/Depends	Unimportant	Should not be included
○	○	○	○	○

Advance Directive

An advance directive is a document describing how the person wants to be treated when they are unable to make their own decisions due to their present state of illness. Advance directives should specify the circumstances under which they will be used and who will take the various actions specified, e.g. when the person is becoming unwell or when they are acutely unwell.

*274. After diagnosis, the helper should discuss an advance directive with the person on the relevant topics, e.g. care preferences, management of finances and arrangements after death.

Essential	Important	Don't know/Depends	Unimportant	Should not be included
○	○	○	○	○

Power of attorney

A power of attorney is where another person is appointed to carry out specified or all legal or financial matters for someone.

*275. After diagnosis, the helper should discuss assigning a power of attorney, e.g. management of finances and health care decisions.

Essential	Important	Don't know/Depends	Unimportant	Should not be included
○	○	○	○	○

***276. The helper should arrange times with the person to discuss the person's wishes, concerns and any preferences for the future.**

Essential	Important	Don't know/Depends	Unimportant	Should not be included
○	○	○	○	○

***277. The helper should tell the person that any decisions made about the future can be revisited if the person wishes.**

Essential	Important	Don't know/Depends	Unimportant	Should not be included
○	○	○	○	○

***278. If the person has concerns about facing the future, the helper should reassure the person about the advantages of planning, e.g. "Don't be scared of planning ahead. It will make life much easier in the future."**

Essential	Important	Don't know/Depends	Unimportant	Should not be included
○	○	○	○	○

***279. After diagnosis, the helper should provide the person with follow-up information about the symptoms of dementia and the need for increasing assistance, e.g. the helper might say, "because of your memory and other problems, you may need to let people help you more than you have in the past".**

Essential	Important	Don't know/Depends	Unimportant	Should not be included
○	○	○	○	○

***280. The helper should learn as much as possible about dementia and the person's diagnosis.**

Essential	Important	Don't know/Depends	Unimportant	Should not be included
○	○	○	○	○

***281. The helper should be aware that it may be helpful for the person and for themselves to share any sadness and concern about the future.**

Essential	Important	Don't know/Depends	Unimportant	Should not be included
○	○	○	○	○

Helping the confused older person

***282. The helper should give the person the opportunity to talk about their experiences and how they see themselves as they lose some of their functioning and capabilities.**

Essential	Important	Don't know/Depends	Unimportant	Should not be included
◯	◯	◯	◯	◯

***283. The helper should give the person information about counselling because it may be useful for the person in coming to terms with the new situation and accepting the limitations the illness imposes.**

Essential	Important	Don't know/Depends	Unimportant	Should not be included
◯	◯	◯	◯	◯

***284. The helper should give the person's family and friends information on the person's disease because the more they learn, the more comfortable they may feel around the person.**

Essential	Important	Don't know/Depends	Unimportant	Should not be included
◯	◯	◯	◯	◯

285. Do you have any comments on the above statements? Is there anything you would like to add? Please write your suggestions in the box provided.

Discussing driving and making decisions with the person

This section concerns conversations and decisions about stopping or restricting driving. In this section, the helper will generally be a family caregiver. Please rate how important (from 'essential' to 'should not be included') you think it is that each statement be included in the guidelines.

When providing first aid to the confused person, the helper will need to use their judgement because a person's decline in cognitive ability may be very mild or more severe. Please keep this in mind when rating the following items.

Please also keep the **definitions** in mind when rating the statements.

Awareness about dementia and driving ability

***286. The helper should be aware that although the person might be driving safely early on, their progressive decline in cognitive abilities means a time will come when they will be at high risk of causing an accident.**

Essential	Important	Don't know/Depends	Unimportant	Should not be included
◯	◯	◯	◯	◯

***287. The helper should be aware that although the person may appear to be driving safely, they may be relying entirely on the habits of driving and may be unable to respond appropriately to a new situation.**

Essential	Important	Don't know/Depends	Unimportant	Should not be included
◯	◯	◯	◯	◯

***288. The helper should be aware that even if the person doesn't appear to be having trouble driving, the person may not be safe to drive because they might not be capable of reacting quickly to an unexpected problem or making a decision needed to avoid an accident.**

Essential	Important	Don't know/Depends	Unimportant	Should not be included
◯	◯	◯	◯	◯

***289. The helper should be aware that the person may forget unsafe driving incidents and therefore have a false sense of confidence about their driving ability.**

Essential	Important	Don't know/Depends	Unimportant	Should not be included
◯	◯	◯	◯	◯

***290. The helper should be aware that an early diagnosis and medications may help the person with dementia drive for longer.**

Essential	Important	Don't know/Depends	Unimportant	Should not be included
◯	◯	◯	◯	◯

***291. The helper should be aware that stopping driving can be a sensitive issue because it may be linked to independence for the person.**

Essential	Important	Don't know/Depends	Unimportant	Should not be included
◯	◯	◯	◯	◯

Planning for future driving decisions

***292. The helper should talk to the person about driving issues, e.g. safety and liability.**

Essential	Important	Don't know/Depends	Unimportant	Should not be included
◯	◯	◯	◯	◯

***293. The helper should encourage the person to begin to plan for when they stop driving, e.g. what transport they will use, setting up automatic bill payment online and delivery services.**

Essential	Important	Don't know/Depends	Unimportant	Should not be included
◯	◯	◯	◯	◯

***294. The helper should suggest that the person have some 'no driving days' so that they become familiar with other transport alternatives for when they will no longer be able to drive.**

Essential	Important	Don't know/Depends	Unimportant	Should not be included
◯	◯	◯	◯	◯

***295. The helper should help the person put a written agreement in place regarding under what future circumstances the person's driving should be restricted.**

Essential	Important	Don't know/Depends	Unimportant	Should not be included
◯	◯	◯	◯	◯

***296. The helper should begin discussing and planning future driving restrictions <u>with the person</u> shortly after diagnosis of dementia.**

Essential	Important	Don't know/Depends	Unimportant	Should not be included
◯	◯	◯	◯	◯

***297. The helper should begin discussing and planning future driving restrictions <u>with the person's family</u> shortly after diagnosis of dementia.**

Essential	Important	Don't know/Depends	Unimportant	Should not be included
◯	◯	◯	◯	◯

Helping the confused older person

***298. The helper should communicate with the person about their driving early and often, because this can help the person decide on a course of action before an accident occurs.**

Essential	Important	Don't know/Depends	Unimportant	Should not be included
◯	◯	◯	◯	◯

***299. The helper should encourage the person to express what the loss of driving means to them because this may help the transition to not driving in the future.**

Essential	Important	Don't know/Depends	Unimportant	Should not be included
◯	◯	◯	◯	◯

***300. Even if the person is currently safe to drive, the helper should encourage the person diagnosed with dementia to gradually start making the transition from being a driver to being a passenger because this may help ease the adjustment.**

Essential	Important	Don't know/Depends	Unimportant	Should not be included
◯	◯	◯	◯	◯

***301. Even if the person is currently safe to drive, the helper should encourage the person diagnosed with dementia to gradually modify their driving, e.g. drive only on familiar roads, avoid long distances, avoid driving at night or in bad weather.**

Essential	Important	Don't know/Depends	Unimportant	Should not be included
◯	◯	◯	◯	◯

302. Do you have any comments on the above statements? Is there anything you would like to add? Please write your suggestions in the box provided.

Discussing driving and making decisions with the person (continued)

Helping the confused older person

This section concerns conversations and decisions about stopping or restricting driving. In this section, the helper will generally be a family caregiver. Please rate how important (from 'essential' to 'should not be included') you think it is that each statement be included in the guidelines.

When providing first aid to the confused person, the helper will need to use their judgement because a person's decline in cognitive ability may be very mild or more severe. Please keep this in mind when rating the following items.

Please also keep the **definitions** in mind when rating the statements.

How to know when the person should stop driving

***303. The helper should be aware that no examination or single indicator exists to determine when the person poses a danger to themselves or others by driving.**

Essential	Important	Don't know/Depends	Unimportant	Should not be included
◯	◯	◯	◯	◯

***304. The helper should not allow the person with any signs of dementia to drive because the safety risk is too great.**

Essential	Important	Don't know/Depends	Unimportant	Should not be included
◯	◯	◯	◯	◯

***305. The helper should be aware that a diagnosis of dementia is not automatically a reason to take away the right to drive.**

Essential	Important	Don't know/Depends	Unimportant	Should not be included
◯	◯	◯	◯	◯

***306. If the person is diagnosed with dementia, the helper should discourage driving soon after the diagnosis.**

Essential	Important	Don't know/Depends	Unimportant	Should not be included
◯	◯	◯	◯	◯

***307. If the person is diagnosed with dementia, the helper should help the person to decide whether it is safe for them to continue to drive.**

Essential	Important	Don't know/Depends	Unimportant	Should not be included
◯	◯	◯	◯	◯

***308. The helper should trust their assessment of the person's driving, as this is likely to be a good predictor of actual safety.**

Essential	Important	Don't know/Depends	Unimportant	Should not be included
◯	◯	◯	◯	◯

***309. If the helper is concerned about the safety of the person's driving, the helper should:**

	Essential	Important	Don't know/ depends	Unimportant	Should not be included
*share observations of the person's unsafe driving with the person, other family members and healthcare providers.	◯	◯	◯	◯	◯
*ask the person's health professionals to raise questions about driving safety with the person.	◯	◯	◯	◯	◯
*contact your local driver licensing authority to discuss your concerns.	◯	◯	◯	◯	◯
*encourage the person to have a driving assessment, e.g. occupational therapy driving assessment.	◯	◯	◯	◯	◯
*encourage the person to have regular driving reassessments every six months.	◯	◯	◯	◯	◯
*monitor the person's driving frequently, at least once a month.	◯	◯	◯	◯	◯

310. Do you have any comments on the above statements? Is there anything you would like to add? Please write your suggestions in the box provided.

Discussing driving and making decisions with the person (continued)

This section concerns conversations and decisions about stopping or restricting driving. In this section, the helper will generally be a family caregiver. Please rate how important (from 'essential' to 'should not be included') you think it is that each statement be included in the guidelines.

When providing first aid to the confused person, the helper will need to use their judgement because a person's decline in cognitive ability may be very mild or more severe. Please keep this in mind when rating the following items.

Please also keep the **definitions** in mind when rating the statements.

How to know when the person should stop driving

***311. The helper should determine when the person should stop driving by:**

	Essential	Important	Don't know/ depends	Unimportant	Should not be included
*considering whether they or others feel uncomfortable driving with the person.	◯	◯	◯	◯	◯
*considering whether they would want a child or grandchild to be driven by the person.	◯	◯	◯	◯	◯
*asking family and friends about the person's driving performance.	◯	◯	◯	◯	◯
*finding out if the person has been involved in any recent accidents whilst driving.	◯	◯	◯	◯	◯
*finding out if the person is more frequently becoming lost while driving.	◯	◯	◯	◯	◯
*asking the person to have a driving assessment, e.g. occupational therapy driving assessment.	◯	◯	◯	◯	◯
*documenting the dates and incidents which indicate the person's driving ability has declined, e.g. failing to signal when changing lanes, driving too fast or too slow, braking late or early.	◯	◯	◯	◯	◯
*observing if the person is modifying their driving behaviour to accommodate changes in skill, e.g. driving shorter distances, driving only on familiar roads, avoiding night driving.	◯	◯	◯	◯	◯

***312. The helper should not try to keep the person driving longer by acting as a 'co-pilot' (e.g. by giving instruction and directions to the person when they drive), because in an emergency situation there is rarely enough time for instructions to be given and acted upon to avoid an accident.**

Essential	Important	Don't know/Depends	Unimportant	Should not be included
◯	◯	◯	◯	◯

***313. The helper should assess the person's potential of having a car accident by having the person sit in the passenger seat and asking them to give specific instructions about how to drive.**

Essential	Important	Don't know/Depends	Unimportant	Should not be included
○	○	○	○	○

***314. The helper should inform the person that if they have been diagnosed with a condition that affects their driving ability they may not be covered by insurance if they have an accident.**

Essential	Important	Don't know/Depends	Unimportant	Should not be included
○	○	○	○	○

***315. The helper should be aware of relevant local laws regarding driving after a diagnosis of dementia.**

Essential	Important	Don't know/Depends	Unimportant	Should not be included
○	○	○	○	○

***316. The helper should include the person, as far as possible, in decision making regarding any driving restrictions.**

Essential	Important	Don't know/Depends	Unimportant	Should not be included
○	○	○	○	○

***317. If it is clear that the person can no longer drive safely, the helper should have a discussion with the person about stopping driving.**

Essential	Important	Don't know/Depends	Unimportant	Should not be included
○	○	○	○	○

***318. If it is clear that the person can no longer drive safely, the helper must not delay in taking the necessary steps to stop the person driving.**

Essential	Important	Don't know/Depends	Unimportant	Should not be included
○	○	○	○	○

Unsafe driving incidents include moving into a wrong lane, hitting curbs, driving at inappropriate speeds, delayed responses to unexpected situations, not anticipating dangerous situations, increased agitation or irritation when driving, getting lost in familiar places, near misses.

Helping the confused older person

***319. If the person has had an increase in frequency of <u>unsafe driving incidents</u>, the helper should have a discussion with them about restricting their driving.**

Essential	Important	Don't know/Depends	Unimportant	Should not be included
○	○	○	○	○

***320. If the person has had an increase in frequency of <u>unsafe driving incidents</u>, the helper should act to restrict the person's driving.**

Essential	Important	Don't know/Depends	Unimportant	Should not be included
○	○	○	○	○

321. Do you have any comments on the above statements? Is there anything you would like to add? Please write your suggestions in the box provided.

Discussing driving and making decisions with the person (continued)

This section concerns conversations and decisions about stopping or restricting driving. In this section, the helper will generally be a family caregiver. Please rate how important (from 'essential' to 'should not be included') you think it is that each statement be included in the guidelines.

When providing first aid to the confused person, the helper will need to use their judgement because a person's decline in cognitive ability may be very mild or more severe. Please keep this in mind when rating the following items.

Please also keep the **definitions** in mind when rating the statements.

<u>How to broach the subject of driving safety with the person</u>

***322. If the helper is concerned about the person's driving ability, they should have a frank discussion with the person about these concerns.**

Essential	Important	Don't know/Depends	Unimportant	Should not be included
○	○	○	○	○

***323. If the helper is having a frank discussion with the person about concerns over their driving ability, they should ask others to be present who can lend further support to the case.**

Essential	Important	Don't know/Depends	Unimportant	Should not be included
○	○	○	○	○

***324. If the helper is having a frank discussion with the person about concerns over their driving ability, they should bring any records of the person's driving that indicate the decline in the person's driving ability.**

Essential	Important	Don't know/Depends	Unimportant	Should not be included
○	○	○	○	○

***325. If the helper is going to have a frank discussion with the person about concerns over their driving ability, they should be prepared for a wide range of reactions, e.g. sadness, relief, anger, defensiveness.**

Essential	Important	Don't know/Depends	Unimportant	Should not be included
○	○	○	○	○

***326. If the person has made a prior written agreement about when to limit or stop driving, the helper should use this document to initiate a conversation about driving restrictions.**

Essential	Important	Don't know/Depends	Unimportant	Should not be included
○	○	○	○	○

***327. The helper should have the discussion about driving safety each time there is a change in the person's medications or health status.**

Essential	Important	Don't know/Depends	Unimportant	Should not be included
○	○	○	○	○

***328. The helper should initiate a discussion about driving in a way that will not lead the person to be defensive about their abilities, e.g. Rather than saying, "Your driving is terrible, you are getting lost, and you're just not safe", the helper should say "You are getting absent-minded about stop lights."**

Essential	Important	Don't know/Depends	Unimportant	Should not be included
○	○	○	○	○

***329. When discussing driving restrictions, the helper should:**

	Essential	Important	Don't know/ depends	Unimportant	Should not be included
*acknowledge how difficult it may be for the person to give up driving.	◯	◯	◯	◯	◯
*look for ways to help the person save face and maintain their self esteem, because giving up driving can mean the person admitting their increasing limitations.	◯	◯	◯	◯	◯
*listen to the concerns of the person because it will be helpful for them to feel as if their concerns and feelings are being recognised and heard.	◯	◯	◯	◯	◯
*make the point that everyone will have to stop driving at some point.	◯	◯	◯	◯	◯
*focus on the nature of the disease, i.e. that many people with dementia have safe past driving records, but this does not mean they will be safe as a driver with dementia in the future.	◯	◯	◯	◯	◯

***330. The helper should use appeals about safety when talking to the person about driving in the earlier stages of their cognitive decline.**

Essential	Important	Don't know/Depends	Unimportant	Should not be included
◯	◯	◯	◯	◯

***331. When the helper uses appeals to safety when talking to the person about driving, they should cite specific driving incidents because this may help convince the person to restrict their driving.**

Essential	Important	Don't know/Depends	Unimportant	Should not be included
◯	◯	◯	◯	◯

***332. The helper should avoid making a rational argument with the person who has been advised not to drive.**

Essential	Important	Don't know/Depends	Unimportant	Should not be included
◯	◯	◯	◯	◯

Helping the confused older person

***333. Once the decision is made that the person should stop driving, the helper should stand by the decision.**

Essential	Important	Don't know/Depends	Unimportant	Should not be included
○	○	○	○	○

334. Do you have any comments on the above statements? Is there anything you would like to add? Please write your suggestions in the box provided.

Discussing driving and making decisions with the person (continued)

This section concerns conversations and decisions about stopping or restricting driving. In this section, the helper will generally be a family caregiver. Please rate how important (from 'essential' to 'should not be included') you think it is that each statement be included in the guidelines.

When providing first aid to the confused person, the helper will need to use their judgement because a person's decline in cognitive ability may be very mild or more severe. Please keep this in mind when rating the following items.

Please also keep the **definitions** in mind when rating the statements.

What to do if the discussion doesn't go well

***335. If the person is reluctant to talk about driving, the helper should ask the person's health professional to bring up the subject of driving during an appointment.**

Essential	Important	Don't know/Depends	Unimportant	Should not be included
○	○	○	○	○

***336. If the discussion with the person does not go well, the helper should remain patient, firm and empathetic.**

Essential	Important	Don't know/Depends	Unimportant	Should not be included
○	○	○	○	○

***337. If the discussion with the person does not go well, the helper should not blame themselves and remember that the person's impaired insight may be making it difficult to understand that their driving is no longer safe.**

Essential	Important	Don't know/Depends	Unimportant	Should not be included
○	○	○	○	○

***338. If the person does not agree to stop driving, the helper should enlist a professional mediator to help, as this will provide a neutral approach that lets the person feel heard and respected.**

Essential	Important	Don't know/Depends	Unimportant	Should not be included
○	○	○	○	○

How to help the person give up driving

***339. The helper should help the person to reduce their driving gradually over time rather than all at once.**

Essential	Important	Don't know/Depends	Unimportant	Should not be included
○	○	○	○	○

***340. Once the decision is made that the person should restrict driving, the helper should be consistent and vigilant to ensure that the person adheres to any driving restrictions.**

Essential	Important	Don't know/Depends	Unimportant	Should not be included
○	○	○	○	○

***341. If the person is able, the helper should encourage them to take charge of their new transport arrangements, e.g. by getting details of local transport services, arranging transport with friends or family.**

Essential	Important	Don't know/Depends	Unimportant	Should not be included
○	○	○	○	○

***342. The helper should help the person give up driving by offering alternatives, e.g. "I'll drive today and you can look at the scenery".**

Essential	Important	Don't know/Depends	Unimportant	Should not be included
○	○	○	○	○

***343. The helper should help the person give up driving by distracting the person from feeling the need to go out driving.**

Essential	Important	Don't know/Depends	Unimportant	Should not be included
○	○	○	○	○

***344. The helper should ask the person's health professional to tell the person that they are no longer safe to drive.**

Essential	Important	Don't know/Depends	Unimportant	Should not be included
○	○	○	○	○

***345. The helper should try to minimise the impact of any driving restrictions placed upon the person, e.g. if possible, be available to drive the person, arrange for home deliveries of medication and groceries.**

Essential	Important	Don't know/Depends	Unimportant	Should not be included
○	○	○	○	○

***346. The helper should be aware they may need to remind the person that they should not be driving because they may forget or decide to continue.**

Essential	Important	Don't know/Depends	Unimportant	Should not be included
○	○	○	○	○

***347. To help the person transition to not driving, the helper should ask the person's family members, friends and neighbours to support the person emotionally, socially and practically, e.g. by visiting the person and helping with transportation.**

Essential	Important	Don't know/Depends	Unimportant	Should not be included
○	○	○	○	○

***348. The helper should find out if there are support groups available locally for the person who is giving up driving and offer this information to the person.**

Essential	Important	Don't know/Depends	Unimportant	Should not be included
○	○	○	○	○

***349. If the helper requires further assistance in dealing with the person's driving they should call a dementia or Alzheimer's helpline for assistance.**

Essential	Important	Don't know/Depends	Unimportant	Should not be included
○	○	○	○	○

Helping the confused older person

***350. If the person has recently moved home, the helper should use the unfamiliarity of the surroundings as an opportunity to encourage the person to limit or stop driving.**

Essential	Important	Don't know/Depends	Unimportant	Should not be included
◯	◯	◯	◯	◯

***351. The helper should build a case for selling the person's car by itemising the costs of maintaining and operating a car.**

Essential	Important	Don't know/Depends	Unimportant	Should not be included
◯	◯	◯	◯	◯

What to do if the person wants to keep driving when it is not safe for them to do so

***352. If the person is not convinced they should no longer drive, the helper should enlist the help of the person's doctor to write a 'prescription' to stop driving.**

Essential	Important	Don't know/Depends	Unimportant	Should not be included
◯	◯	◯	◯	◯

***353. If the person is not convinced they should no longer drive, both the person and the helper should agree to abide by the results of a driving assessment, e.g. an occupational therapy driving assessment.**

Essential	Important	Don't know/Depends	Unimportant	Should not be included
◯	◯	◯	◯	◯

***354. If the person is insistent on driving, the helper should find ways to make driving impossible for the person, e.g. disabling the car in some way, hiding the keys.**

Essential	Important	Don't know/Depends	Unimportant	Should not be included
◯	◯	◯	◯	◯

355. Do you have any comments on the above statements? Is there anything you would like to add? Please write your suggestions in the box provided.

Care discussions and decisions

Helping the confused older person

This section concerns conversations and decisions about level of care and living arrangements. In this section, the helper will generally be a family caregiver. Please rate how important (from 'essential' to 'should not be included') you think it is that each statement be included in the guidelines.

When providing first aid to the confused person, the helper will need to use their judgement because a person's decline in cognitive ability may be very mild or more severe. Please keep this in mind when rating the following items.

Please also keep the **definitions** in mind when rating the statements.

Awareness about care decisions

***356. The helper should be aware that a diagnosis of dementia does not automatically mean that the person is incapable of living alone.**

Essential	Important	Don't know/Depends	Unimportant	Should not be included
◯	◯	◯	◯	◯

***357. The helper should be aware that, if they are providing care for the person, the time may come when they can no longer provide the kind of care that the person requires.**

Essential	Important	Don't know/Depends	Unimportant	Should not be included
◯	◯	◯	◯	◯

***358. The helper should be aware that due to the progressive decline of dementia, it is likely that the person will reach a point where they will be safer in residential care.**

Essential	Important	Don't know/Depends	Unimportant	Should not be included
◯	◯	◯	◯	◯

***359. The helper should be aware that the person may be concerned that a move away from home would mean a loss of independence and control in their daily lives.**

Essential	Important	Don't know/Depends	Unimportant	Should not be included
◯	◯	◯	◯	◯

***360. The helper should remember that moving the person to residential care is not a failure on anyone's part, but rather is necessitated by a need for greater care due to the disease.**

Essential	Important	Don't know/Depends	Unimportant	Should not be included
◯	◯	◯	◯	◯

Helping the confused older person

***361. The helper should try to accept that they are not betraying the person by considering residential care for them.**

Essential	Important	Don't know/Depends	Unimportant	Should not be included
○	○	○	○	○

***362. The helper should be aware that a move to residential care does not mean that they must completely give up any caring role they have.**

Essential	Important	Don't know/Depends	Unimportant	Should not be included
○	○	○	○	○

***363. The helper should be aware that if the person moves to residential care, this may improve the relationship between the person and the helper because time together may be less stressful and free from the worries of practical care.**

Essential	Important	Don't know/Depends	Unimportant	Should not be included
○	○	○	○	○

***364. The helper should be aware that the earlier the option of residential care is explored, the better chance there is that the person's preferences for their future care can be taken into account.**

Essential	Important	Don't know/Depends	Unimportant	Should not be included
○	○	○	○	○

***365. The helper should be aware that it is best practice to move the person's place of living as infrequently as possible.**

Essential	Important	Don't know/Depends	Unimportant	Should not be included
○	○	○	○	○

***366. The helper should be aware that the person may not realise the impact that their care needs have on others.**

Essential	Important	Don't know/Depends	Unimportant	Should not be included
○	○	○	○	○

367. Do you have any comments on the above statements? Is there anything you would like to add? Please write your suggestions in the box provided.

Care discussions and decisions (continued)

This section concerns conversations and decisions about level of care and living arrangements. In this section, the helper will generally be a family caregiver. Please rate how important (from ‘essential’ to ‘should not be included’) you think it is that each statement be included in the guidelines.

When providing first aid to the confused person, the helper will need to use their judgement because a person's decline in cognitive ability may be very mild or more severe. Please keep this in mind when rating the following items.

Please also keep the **definitions** in mind when rating the statements.

Planning for future care decisions after a diagnosis

***368. The helper should include the person in discussions concerning their care and living arrangements where possible.**

Essential	Important	Don't know/Depends	Unimportant	Should not be included
○	○	○	○	○

***369. The helper should make sure the person feels included in the decision making and preparation, as this can help the transition, e.g. touring a residential care home together.**

Essential	Important	Don't know/Depends	Unimportant	Should not be included
○	○	○	○	○

***370. The helper should encourage the person who has received a diagnosis of dementia to make decisions in advance about their future care, e.g. what the person would want to happen if their current living arrangement became too difficult.**

Essential	Important	Don't know/Depends	Unimportant	Should not be included
○	○	○	○	○

***371. In planning for future care decisions with the person, the helper should consider and discuss with the person the range of factors that may impact upon care and living arrangements during the course of the disease, e.g. the health of family members, financial matters, the different stages of the decline.**

Essential	Important	Don't know/Depends	Unimportant	Should not be included
○	○	○	○	○

***372. The helper should include other key people (e.g. the person's family and health professional) in open and honest discussions concerning the person's care and living arrangements so they can help and support the decision to make a change when the time comes.**

Essential	Important	Don't know/Depends	Unimportant	Should not be included
○	○	○	○	○

***373. The helper should talk to the person's health professional early on about what can be done to smooth the transition to residential care, if there is an eventual need.**

Essential	Important	Don't know/Depends	Unimportant	Should not be included
○	○	○	○	○

***374. The helper should remember that their own physical and emotional health is as important as that of the person.**

Essential	Important	Don't know/Depends	Unimportant	Should not be included
○	○	○	○	○

***375. If the person has impaired insight about the need for a possible move, the helper should limit the person's involvement in the planning process.**

Essential	Important	Don't know/Depends	Unimportant	Should not be included
○	○	○	○	○

***376. As the dementia progresses, the helper should monitor the person's living situation carefully for risks to safety.**

Essential	Important	Don't know/Depends	Unimportant	Should not be included
○	○	○	○	○

***377. The helper should be aware of the range of care services offered in the person's community that may be useful at different stages of the person's dementia, e.g. respite, day centres, supported residential care.**

Essential	Important	Don't know/Depends	Unimportant	Should not be included
○	○	○	○	○

***378. The helper should make early contact with any local aged care assessment services to learn about what levels of care may be available to the person in the future.**

Essential	Important	Don't know/Depends	Unimportant	Should not be included
○	○	○	○	○

***379. The helper should familiarise themselves with any eligibility criteria for residential care or other services that the person may need in the future.**

Essential	Important	Don't know/Depends	Unimportant	Should not be included
○	○	○	○	○

***380. The helper should familiarise themselves with any eligibility criteria for residential care or other services that the person may need in the future.**

Essential	Important	Don't know/Depends	Unimportant	Should not be included
○	○	○	○	○

***381. The helper should know what respite options are available in the event that they have their own health problem and the person needs to temporarily get care elsewhere.**

Essential	Important	Don't know/Depends	Unimportant	Should not be included
○	○	○	○	○

***382. The helper should ensure they have a plan for the person's care in the event that something happens to them and they can no longer provide care.**

Essential	Important	Don't know/Depends	Unimportant	Should not be included
○	○	○	○	○

<u>**Living alone, respite care and other support services**</u>

Helping the confused older person

***383. The helper should consider introducing outside help or support services to help care for the person in the early stages of the person's dementia, because it may be accepted more easily at this stage than later on.**

Essential	Important	Don't know/Depends	Unimportant	Should not be included
○	○	○	○	○

***384. The helper should be aware that the person needs to live in environments that best support their safety and quality of life and this may mean living at home with support services, even if there is some risk.**

Essential	Important	Don't know/Depends	Unimportant	Should not be included
○	○	○	○	○

***385. The helper should identify any risks to the person's living arrangement and work with the person's health care professionals to lessen them where possible, e.g. using meal delivery services if cooking becomes unsafe.**

Essential	Important	Don't know/Depends	Unimportant	Should not be included
○	○	○	○	○

386. Do you have any comments on the above statements? Is there anything you would like to add? Please write your suggestions in the box provided.

Care discussions and decisions (continued)

This section concerns conversations and decisions about level of care and living arrangements. In this section, the helper will generally be a family caregiver. Please rate how important (from 'essential' to 'should not be included') you think it is that each statement be included in the guidelines.

When providing first aid to the confused person, the helper will need to use their judgement because a person's decline in cognitive ability may be very mild or more severe. Please keep this in mind when rating the following items.

Please also keep the **definitions** in mind when rating the statements.

How to know when the person needs a higher level of care or a more supported living arrangement

Helping the confused older person

***387. The helper should be aware that there is no single indicator to determine when the person should move into residential care and that it varies between people and families.**

Essential	Important	Don't know/Depends	Unimportant	Should not be included
○	○	○	○	○

***388. The helper should be aware that when the person can no longer live safely at home, residential care is usually the best solution.**

Essential	Important	Don't know/Depends	Unimportant	Should not be included
○	○	○	○	○

***389. When deciding whether it is time to change to a higher level of care or a more supported living arrangement for the person, the helper should consider whether:**

	Essential	Important	Don't know/ depends	Unimportant	Should not be included
*the person's values, views, history or preferences would be consistent with the proposed change.	○	○	○	○	○
*the person's communication has changed, e.g. phone calls have slowed or ceased, non-emergency calls are made at unreasonable hours.	○	○	○	○	○
*there are signs in the person's house that show they are not managing e.g. refrigerator has expired food or is empty, unopened mail, unclean.	○	○	○	○	○
*there are changes in the person's social life, e.g. disengaged from previous social activities or relationships, forgetting appointments.	○	○	○	○	○
*there are changes in the person's self-care, e.g. inexplicable weight change, altered grooming standards.	○	○	○	○	○
*the person cannot maintain their personal hygiene.	○	○	○	○	○
*the person is not getting an adequate diet.	○	○	○	○	○
*the person cannot manage their medication	○	○	○	○	○
*there have been particular incidents or problems, e.g. robbery because a stranger was let in or a door was left unlocked.	○	○	○	○	○
*there is risk to the safety of the person or others, e.g. because the person is forgetting to turn off the stove, falling on stairs, unresponsive to emergencies.	○	○	○	○	○
*there are challenging behaviours that require greater supervision, e.g. wandering, aggression.	○	○	○	○	○
*the person cannot handle their day-to-day financial transactions.	○	○	○	○	○
*the risks of the current living arrangement (e.g. safety issues) outweigh the benefits (e.g. independence, familiar location)	○	○	○	○	○
*the strain on caregivers or family has become too great, e.g. night time restlessness is keeping others from getting a good night's sleep, around-	○	○	○	○	○

the-clock care is too stressful or overwhelming.					
*the family caregivers' negative feelings about being with the person have begun to outweigh the positive feelings.	◯	◯	◯	◯	◯
*the person is no longer able to recognise or interact with their environment.	◯	◯	◯	◯	◯
*the person is missing out on social contact.	◯	◯	◯	◯	◯
*the cost of alternative forms of care is affordable.	◯	◯	◯	◯	◯
*there are others who can help with caregiving in the person's current living situation.	◯	◯	◯	◯	◯
*there are suitable alternative forms of care available for the person.	◯	◯	◯	◯	◯

390. Do you have any comments on the above statements? Is there anything you would like to add? Please write your suggestions in the box provided.

***391. The helper should discuss the decision with the person's health care professionals because they can often give their professional opinion on the type of care that someone needs.**

Essential	Important	Don't know/Depends	Unimportant	Should not be included
◯	◯	◯	◯	◯

How to broach the subject of residential care with the person

***392. The helper should be prepared for a range of reactions to the decision, e.g. anger, bitterness, sadness and accusations.**

Essential	Important	Don't know/Depends	Unimportant	Should not be included
◯	◯	◯	◯	◯

***393. If the helper finds it difficult to tell the person that they need to change their current living situation, they should enlist someone to help them, e.g. the person's health professional or someone else they respect.**

Essential	Important	Don't know/Depends	Unimportant	Should not be included
◯	◯	◯	◯	◯

Helping the confused older person

How to handle difficulties on the decision for residential care

***394. If the person is resistant to the idea of a change in their living situation, the helper should be firm about the decision that has been made.**

Essential	Important	Don't know/Depends	Unimportant	Should not be included
○	○	○	○	○

***395. If the helper is distressed about having made the decision to change the person's living situation, they should seek counselling support.**

Essential	Important	Don't know/Depends	Unimportant	Should not be included
○	○	○	○	○

396. Do you have any comments on the above statements? Is there anything you would like to add? Please write your suggestions in the box provided.

Challenging Behaviours

This section asks you what information the helper should know in order to assist a person whose behaviour they are finding challenging.

***Challenging behaviours** refers to behaviours which are difficult, inappropriate, undesirable or unacceptable. For example, this may include agitated, repetitive, aggressive or disinhibited behaviours.*

Please rate how important (from 'essential' to 'should not be included') you think it is that each statement be included in the guidelines.

When providing first aid to the confused person, the helper will need to use their judgement because a person's decline in cognitive ability may be very mild or more severe. Please keep this in mind when rating the following items.

Please also keep the **definitions** in mind when rating the statements.

General challenging behaviours

***397. The helper should be aware that the person may appear uncooperative, inattentive or irrational due to their confusion and is not behaving this way on purpose to annoy or irritate.**

Essential	Important	Don't know/Depends	Unimportant	Should not be included
○	○	○	○	○

***398. The helper should be aware that the person may display challenging behaviours in order to gain a greater sense of control over their life.**

Essential	Important	Don't know/Depends	Unimportant	Should not be included
○	○	○	○	○

***399. If the person is behaving inappropriately, the helper should remember it is their illness causing the behaviour.**

Essential	Important	Don't know/Depends	Unimportant	Should not be included
○	○	○	○	○

***400. The helper should be aware that challenging behaviour by the person may not be meaningless or random and could be because the person has a need that they cannot communicate.**

Essential	Important	Don't know/Depends	Unimportant	Should not be included
○	○	○	○	○

***401. If the helper is finding the person's behaviour challenging, they should try to identify any need underlying the behaviour and try to help the person to meet the need.**

Essential	Important	Don't know/Depends	Unimportant	Should not be included
○	○	○	○	○

***402. In an emotionally charged situation with the person, where the helper believes they will lose control, they should remove themselves, if possible, and return when they feel calmer.**

Essential	Important	Don't know/Depends	Unimportant	Should not be included
○	○	○	○	○

***403. The helper should not try to reason with the person if they no longer have the ability to do so.**

Essential	Important	Don't know/Depends	Unimportant	Should not be included
○	○	○	○	○

***404. The helper should not argue with the person engaging in challenging behaviours.**

Essential	Important	Don't know/Depends	Unimportant	Should not be included
○	○	○	○	○

***405. The helper should not hesitate to tell white lies to the person to make a difficult situation manageable.**

Essential	Important	Don't know/Depends	Unimportant	Should not be included
○	○	○	○	○

***406. If the person is displaying challenging behaviour that the helper wants to stop, the helper should interrupt what the person is doing or distract the person, e.g. ask the person a question about their early life.**

Essential	Important	Don't know/Depends	Unimportant	Should not be included
○	○	○	○	○

***407. If the person is not doing what the helper wants them to do (e.g. eating their meal), the helper should model the action.**

Essential	Important	Don't know/Depends	Unimportant	Should not be included
○	○	○	○	○

408. Do you have any comments on the above statements? Is there anything you would like to add? Please write your suggestions in the box provided.

Challenging Behaviours (continued)

This section asks you what information the helper should know in order to assist a person whose behaviour they are finding challenging.

Challenging behaviours *refers to behaviours which are difficult, inappropriate, undesirable or unacceptable. For example, this may include agitated, repetitive, aggressive or disinhibited behaviours.*

Please rate how important (from ‘essential’ to ‘should not be included’) you think it is that each statement be included in the guidelines.

When providing first aid to the confused person, the helper will need to use their judgement because a person's decline in cognitive ability may be very mild or more severe. Please keep this in mind when rating the following items.

Please also keep the **definitions** in mind when rating the statements.

When the person is agitated, angry or upset

***409. The helper should be aware that loud noises, an over-stimulating environment, or physical clutter often cause agitated behaviour.**

Essential	Important	Don't know/Depends	Unimportant	Should not be included
○	○	○	○	○

***410. If the person becomes agitated, the helper should:**

	Essential	Important	Don't know/ depends	Unimportant	Should not be included
*react calmly.	○	○	○	○	○
*reassure the person that they are safe and that everything is under control.	○	○	○	○	○
*apologise to the person even if the situation isn't their fault.	○	○	○	○	○
*promise the person that they will stay until the person feels better.	○	○	○	○	○
*try using music to help the person to calm down, e.g. singing their favourite song.	○	○	○	○	○

***411. If the person becomes agitated during conversation, the helper should change to another activity or topic of conversation thereby removing the situation that is causing the agitation.**

Essential	Important	Don't know/Depends	Unimportant	Should not be included
○	○	○	○	○

***412. If the person is frightened or agitated, the helper should be careful about touching the person because the person might interpret the touch as a form of restraint and become angry.**

Essential	Important	Don't know/Depends	Unimportant	Should not be included
○	○	○	○	○

***413. The helper should not physically restrain the person because they may feel fenced in and become aggressive.**

Essential	Important	Don't know/Depends	Unimportant	Should not be included
○	○	○	○	○

***414. The helper should be aware that agitation can occur every day at around the same time for a person (e.g. around sunset) and that this agitation will pass.**

Essential	Important	Don't know/Depends	Unimportant	Should not be included
○	○	○	○	○

***415. If the person appears anxious about something, the helper should offer comfort and reassurance rather than responding to the specifics of what the person is trying to say.**

Essential	Important	Don't know/Depends	Unimportant	Should not be included
○	○	○	○	○

***416. If the person becomes angry or upset in public, the helper should stand further back to give the person space and wait it out.**

Essential	Important	Don't know/Depends	Unimportant	Should not be included
○	○	○	○	○

***417. If the person is angry and upset but isn't doing any harm, the helper need not do anything, as this will generally pass quickly.**

Essential	Important	Don't know/Depends	Unimportant	Should not be included
○	○	○	○	○

***418. If the person seems angry it may be because of a misunderstanding, in which case the helper should take the time to explain in simple sentences what is happening.**

Essential	Important	Don't know/Depends	Unimportant	Should not be included
○	○	○	○	○

***419. The helper should be aware of some of the common reasons for anger or aggression in a person with dementia, e.g. situations where the person feels trapped, controlled, fearful, humiliated or helpless, or where there are changes in surroundings or routine.**

Essential	Important	Don't know/Depends	Unimportant	Should not be included
○	○	○	○	○

Helping the confused older person

***420. If the person becomes angry or upset, the helper should remain calm and, if possible, move the person elsewhere in a quiet, unhurried way.**

Essential	Important	Don't know/Depends	Unimportant	Should not be included
◯	◯	◯	◯	◯

***421. If the person is angry or upset, the helper should not argue, explain or restrain, as this might make things worse.**

Essential	Important	Don't know/Depends	Unimportant	Should not be included
◯	◯	◯	◯	◯

422. Do you have any comments on the above statements? Is there anything you would like to add? Please write your suggestions in the box provided.

Challenging Behaviours (continued)

This section asks you what information the helper should know in order to assist a person whose behaviour they are finding challenging.

Challenging behaviours *refers to behaviours which are difficult, inappropriate, undesirable or unacceptable. For example, this may include agitated, repetitive, aggressive or disinhibited behaviours.*

Please rate how important (from 'essential' to 'should not be included') you think it is that each statement be included in the guidelines.

When providing first aid to the confused person, the helper will need to use their judgement because a person's decline in cognitive ability may be very mild or more severe. Please keep this in mind when rating the following items.

Please also keep the **definitions** in mind when rating the statements.

Repetitive behaviour

Repetitive behaviour *includes the repetition of questions, phrases, movements or actions, e.g. pacing.*

***423. If the person says the same thing over and over, the helper should encourage the person to say more about the subject.**

Essential	Important	Don't know/Depends	Unimportant	Should not be included
◯	◯	◯	◯	◯

***424. If the person repeats the same words over and over, the helper should not change the topic or distract the individual as this might break the communication efforts of the person.**

Essential	Important	Don't know/Depends	Unimportant	Should not be included
○	○	○	○	○

***425. If the person asks the same question over and over, the helper should politely answer the first few times and then use distraction to change the topic.**

Essential	Important	Don't know/Depends	Unimportant	Should not be included
○	○	○	○	○

***426. If the person repeatedly asks the same question, the helper should try to guess what the person's underlying concern is and reassure them about this, e.g. a repeated question about the time might be because they are worried they will miss an event.**

Essential	Important	Don't know/Depends	Unimportant	Should not be included
○	○	○	○	○

***427. If the person repeats the same story over and over again, the helper should listen politely, acknowledge the importance of the story, and then try to shift to another topic.**

Essential	Important	Don't know/Depends	Unimportant	Should not be included
○	○	○	○	○

***428. If the person repeats something over and over again, the helper should avoid expressing annoyance as this may upset the person.**

Essential	Important	Don't know/Depends	Unimportant	Should not be included
○	○	○	○	○

***429. The helper should listen to the person even if they are using repetitive or nonsensical words or phrases, because these may have meaning for the person.**

Essential	Important	Don't know/Depends	Unimportant	Should not be included
○	○	○	○	○

***430. The helper should answer repetitive questions because this can be reassuring to the person.**

Essential	Important	Don't know/Depends	Unimportant	Should not be included
○	○	○	○	○

***431. If the person is pacing and it is safe to do so, the helper should let them pace.**

Essential	Important	Don't know/Depends	Unimportant	Should not be included
○	○	○	○	○

432. Do you have any comments on the above statements? Is there anything you would like to add? Please write your suggestions in the box provided.

Challenging Behaviours (continued)

This section asks you what information the helper should know in order to assist a person whose behaviour they are finding challenging.

Challenging behaviours *refers to behaviours which are difficult, inappropriate, undesirable or unacceptable. For example, this may include agitated, repetitive, aggressive or disinhibited behaviours.*

Please rate how important (from ‘essential’ to ‘should not be included’) you think it is that each statement be included in the guidelines.

When providing first aid to the confused person, the helper will need to use their judgement because a person's decline in cognitive ability may be very mild or more severe. Please keep this in mind when rating the following items.

Please also keep the **definitions** in mind when rating the statements.

Aggressive behaviours

Aggressive behaviours *are actions which seem forceful, violent, hostile or destructive. Aggressive behaviours may include being abusive or threatening. This behaviour might be verbal (e.g. abusive language) or physical (e.g. hitting or pinching).*

***433. If the helper is concerned about the person becoming aggressive, they should remove potentially dangerous objects from the environment.**

Essential	Important	Don't know/Depends	Unimportant	Should not be included
○	○	○	○	○

✱434. If the person becomes aggressive, the helper should leave the room and give the person the time and space to calm down.

Essential	Important	Don't know/Depends	Unimportant	Should not be included
◯	◯	◯	◯	◯

✱435. If the person becomes aggressive, the helper should use touch and calming words, making sure to do this in a way that will not make the person feel as if the helper is trying to restrain them.

Essential	Important	Don't know/Depends	Unimportant	Should not be included
◯	◯	◯	◯	◯

✱436. If the person becomes physically aggressive the helper should back away and say, 'Please don't hurt me' or 'Please stop' and remove all other people from the room until the person calms down.

Essential	Important	Don't know/Depends	Unimportant	Should not be included
◯	◯	◯	◯	◯

✱437. If the person becomes physically aggressive the helper should remove themselves and anyone else from the room until the person calms down.

Essential	Important	Don't know/Depends	Unimportant	Should not be included
◯	◯	◯	◯	◯

✱438. The helper should only physically restrain the person if there is risk of harm to self or others and nothing else works.

Essential	Important	Don't know/Depends	Unimportant	Should not be included
◯	◯	◯	◯	◯

✱439. If the person becomes aggressive and anyone is at risk of harm, the helper should contact emergency help.

Essential	Important	Don't know/Depends	Unimportant	Should not be included
◯	◯	◯	◯	◯

Disinhibited behaviours

***Disinhibited behaviours** are actions which seem tactless, rude or offensive. They occur when people don’t follow the usual social rules about what or where to say or do something.*

Helping the confused older person

***440. If the person starts behaving in a disinhibited way, the helper should ignore this behaviour.**

Essential	Important	Don't know/Depends	Unimportant	Should not be included
◯	◯	◯	◯	◯

***441. If the person starts behaving in a disinhibited way, the helper should gently correct or divert them.**

Essential	Important	Don't know/Depends	Unimportant	Should not be included
◯	◯	◯	◯	◯

***442. If the person starts behaving in a disinhibited way, the helper should ignore this behaviour as far as possible and then, if necessary, gently correct or divert them.**

Essential	Important	Don't know/Depends	Unimportant	Should not be included
◯	◯	◯	◯	◯

***443. The helper should react with patience and gentleness to disinhibited behaviours, even though they may be embarrassing.**

Essential	Important	Don't know/Depends	Unimportant	Should not be included
◯	◯	◯	◯	◯

***444. The helper should not correct or confront any disinhibited behaviour by the person because the behaviour is not deliberate.**

Essential	Important	Don't know/Depends	Unimportant	Should not be included
◯	◯	◯	◯	◯

***445. The helper should not make fun of any disinhibited behaviour.**

Essential	Important	Don't know/Depends	Unimportant	Should not be included
◯	◯	◯	◯	◯

***446. If the person starts swearing inappropriately, the helper should use the same swear words in a normal conversation so the person will become uninterested in the swear words.**

Essential	Important	Don't know/Depends	Unimportant	Should not be included
◯	◯	◯	◯	◯

***447. If the person engages in inappropriate sexual behaviour, the helper should remind the person that the behaviour is inappropriate and guide them to a private place.**

Essential	Important	Don't know/Depends	Unimportant	Should not be included
○	○	○	○	○

448. Do you have any comments on the above statements? Is there anything you would like to add? Please write your suggestions in the box provided.

Challenging Behaviours (continued)

This section asks you what information the helper should know in order to assist a person whose behaviour they are finding challenging.

***Challenging behaviours** refers to behaviours which are difficult, inappropriate, undesirable or unacceptable. For example, this may include agitated, repetitive, aggressive or disinhibited behaviours.*

Please rate how important (from 'essential' to 'should not be included') you think it is that each statement be included in the guidelines.

When providing first aid to the confused person, the helper will need to use their judgement because a person's decline in cognitive ability may be very mild or more severe. Please keep this in mind when rating the following items.

Please also keep the **definitions** in mind when rating the statements.

Different realities, hallucinations and delusions

***Hallucinations** are false perceptions. A person who is experiencing an hallucination might hear, see, feel or taste things that are not actually there.*

***Delusions** are fixed false beliefs. Although the delusions might seem bizarre, they are very real to the person experiencing them.*

***Confabulation** is the filling in of gaps in a person's memory with fabrications that they believes to be true.*

***449. The helper should be aware that the person may confabulate to cope with the confusion they are experiencing.**

Essential	Important	Don't know/Depends	Unimportant	Should not be included
○	○	○	○	○

***450. If the person is experiencing a different reality (e.g. believes they are in a different time or place), the helper should not try to correct this.**

Essential	Important	Don't know/Depends	Unimportant	Should not be included
○	○	○	○	○

Helping the confused older person

***451. If the person is experiencing a different reality, the helper should go along with their reality so as not to provoke anxiety.**

Essential	Important	Don't know/Depends	Unimportant	Should not be included
◯	◯	◯	◯	◯

***452. If the person is experiencing a different reality, the helper should help them by first going along with their reality, with empathy and sensitivity, and then trying to divert the person's attention back to the present.**

Essential	Important	Don't know/Depends	Unimportant	Should not be included
◯	◯	◯	◯	◯

***453. Instead of arguing with the person about whether their experience is real, the helper should respond to the person's emotional tone (e.g. fear, anger, frustration) and provide comfort and reassurance, e.g. remind the person that you are with them, tell them they are safe.**

Essential	Important	Don't know/Depends	Unimportant	Should not be included
◯	◯	◯	◯	◯

***454. If the person is hallucinating, the helper should :**

	Essential	Important	Don't know/ depends	Unimportant	Should not be included
*not argue about whether the voices or sights are there because the hallucination is real to the person who experiences it and reasoning will not make the hallucination go away.	○	○	○	○	○
*respond in a calm, supportive manner and offer reassurance.	○	○	○	○	○
*approach them cautiously, trying not to startle or frighten them.	○	○	○	○	○
*ask about the feelings behind the hallucinations and empathise with the person.	○	○	○	○	○
*provide a distraction because the person may be able to "tune out" the hallucination by focusing on something else.	○	○	○	○	○
*distract the person by getting them to focus on the helper, e.g. hold their hand or tap them on the shoulder.	○	○	○	○	○
*distract the person, e.g. start a conversation, take them into a well-lit room.	○	○	○	○	○
*should try to reduce factors that may be contributing to the hallucination, e.g. unclear background noises, darkness, being alone.	○	○	○	○	○

***455. If the person is hallucinating but not bothered by it, the helper does not need to take any action.**

Essential	Important	Don't know/Depends	Unimportant	Should not be included
○	○	○	○	○

***456. If the person has been hallucinating, the helper should check if the person has had enough to eat and drink and had enough sleep.**

Essential	Important	Don't know/Depends	Unimportant	Should not be included
○	○	○	○	○

***457. If the person is having a disturbing hallucination, the helper should give all the comfort they would give if their experience was real.**

Essential	Important	Don't know/Depends	Unimportant	Should not be included
○	○	○	○	○

***458. If the person is hallucinating or has a delusional belief, the helper should not try to explain or clarify what is really happening, as this will not help and may make the situation worse by increasing the person's anxiety.**

Essential	Important	Don't know/Depends	Unimportant	Should not be included
○	○	○	○	○

***459. If the person is frightened by the hallucinations and delusions they are experiencing, the helper should acknowledge the person's fears.**

Essential	Important	Don't know/Depends	Unimportant	Should not be included
○	○	○	○	○

***460. If the person has hallucinations or delusions, and the person is very distressed and cannot be calmed down, the helper should contact the person's doctor.**

Essential	Important	Don't know/Depends	Unimportant	Should not be included
○	○	○	○	○

***461. If the person has a delusion or hallucination, the helper should not collude with them, but should avoid confrontation, e.g. "I didn't hear anything, but I know you are frightened. I'll look around to make sure everything is okay."**

Essential	Important	Don't know/Depends	Unimportant	Should not be included
○	○	○	○	○

***462. The helper should be aware that the person may appear to have a delusion because they misidentify people or misinterpret situations and feel threatened.**

Essential	Important	Don't know/Depends	Unimportant	Should not be included
○	○	○	○	○

***463. If the person is deluded that someone is stealing from them, the helper should offer to locate the missing items.**

Essential	Important	Don't know/Depends	Unimportant	Should not be included
○	○	○	○	○

***464. If the person is making unfounded accusations, the helper should immediately agree with the person's version of events.**

Essential	Important	Don't know/Depends	Unimportant	Should not be included
◯	◯	◯	◯	◯

***465. If the person is making unfounded accusations, the helper should offer a more acceptable explanation for the person's concerns, e.g. to the accusation, "You stole my special coat," the helper might answer, "I forgot to tell you that I put it in storage for the summer," even if the coat was sold twenty years ago.**

Essential	Important	Don't know/Depends	Unimportant	Should not be included
◯	◯	◯	◯	◯

466. Do you have any comments on the above statements? Is there anything you would like to add? Please write your suggestions in the box provided.

Delirium

This section asks you what information the helper should know in order to assist and interact with a person who has delirium. Please rate how important (from 'essential' to 'should not be included') you think it is that each statement be included in the guidelines.

***Delirium** is a condition where a sudden and obvious worsening of a person's usual level of functioning appears over hours or days. Delirium can involve problems with attention, awareness, orientation to environment and other areas of cognitive functioning. Delirium is due to illness or environmental factors such as medication.*

When providing first aid to the confused person, the helper will need to use their judgement because a person's decline in cognitive ability may be very mild or more severe. Please keep this in mind when rating the following items.

Please also keep the **definitions** in mind when rating the statements.

How to assist the person with suspected delirium

***467. If the helper suspects the person is experiencing delirium and their behaviour is a serious risk to their safety and the safety of others, the helper should take the person to hospital or call an ambulance.**

Essential	Important	Don't know/Depends	Unimportant	Should not be included
◯	◯	◯	◯	◯

***468. If the helper suspects the person is experiencing delirium, the helper should contact a doctor immediately to inform them of the sudden changes in the person and arrange an appointment.**

Essential	Important	Don't know/Depends	Unimportant	Should not be included
○	○	○	○	○

***469. If the helper suspects the person is experiencing delirium, the helper should write down their observations about the sudden changes in behaviour and physical function (e.g. when the confusion/other problems began), because this will be helpful information for medical professionals.**

Essential	Important	Don't know/Depends	Unimportant	Should not be included
○	○	○	○	○

***470. When the helper takes the person with suspected delirium to medical help, the helper should be prepared to provide information about the person's changes in behaviour or physical function, past medical history and current medications, noting any new medications or changes in dosage.**

Essential	Important	Don't know/Depends	Unimportant	Should not be included
○	○	○	○	○

How to interact with the person who has delirium

***471. The helper should be aware that it can be comforting for the person experiencing delirium to see familiar faces and friends, especially if the person is in hospital.**

Essential	Important	Don't know/Depends	Unimportant	Should not be included
○	○	○	○	○

***472. The helper should be aware that being in a delirious state may reawaken other past distressing or frightening experiences for the person and these may be experienced as reality or as part of a dreamlike state.**

Essential	Important	Don't know/Depends	Unimportant	Should not be included
○	○	○	○	○

***473. If the helper is caring for a person with delirium, they should:**

	Essential	Important	Don't know/ depends	Unimportant	Should not be included
*talk clearly and slowly.	◯	◯	◯	◯	◯
*ensure the person gets adequate food and fluids.	◯	◯	◯	◯	◯
*orientate the person to their environment, i.e. remind them where they are, what time of day it is and who you are.	◯	◯	◯	◯	◯
*only allow visits from those the person knows well.	◯	◯	◯	◯	◯
*inform any visitors of what to expect.	◯	◯	◯	◯	◯
*diminish distracting loud noises such as radio and television.	◯	◯	◯	◯	◯
*ensure there is adequate lighting.	◯	◯	◯	◯	◯
*ensure a person has their comfort items such as familiar blankets, photos and favourite music or clothing.	◯	◯	◯	◯	◯
*check that the person is wearing their hearing aids or glasses.	◯	◯	◯	◯	◯
*protect the person from falls and dangerous objects.	◯	◯	◯	◯	◯
*not restrain a person.	◯	◯	◯	◯	◯
*try placing a mattress on the floor for night time waking.	◯	◯	◯	◯	◯
*discourage day time sleeping.	◯	◯	◯	◯	◯
*avoid sudden movements that may frighten them.	◯	◯	◯	◯	◯
*not take it personally if the person fails to recognise the helper. *introduce themselves each time, if necessary.	◯	◯	◯	◯	◯
*try not to over-excite the person with too much activity.	◯	◯	◯	◯	◯
*use nicknames or other familiar phrases that are likely to be reassuring.	◯	◯	◯	◯	◯
*let staff know if there is anything they could say or do that will make the person feel more at ease or reassured.	◯	◯	◯	◯	◯

Helping the confused older person

***474. If the helper knows that the person experiencing delirium has had previous experiences of trauma (e.g. being trapped, frightened or very ill), the helper should tell the health professional.**

Essential	Important	Don't know/Depends	Unimportant	Should not be included
○	○	○	○	○

***475. The helper should monitor the person experiencing delirium, because they are more at risk of falls than other older people.**

Essential	Important	Don't know/Depends	Unimportant	Should not be included
○	○	○	○	○

***476. To reduce the risk of falls, the helper should assist the person experiencing delirium with walking and moving from a sitting or lying position.**

Essential	Important	Don't know/Depends	Unimportant	Should not be included
○	○	○	○	○

477. Do you have any comments on the above statements? Is there anything you would like to add? Please write your suggestions in the box provided.

Feedback and thank you

478. 1. Did anything in these surveys cause you to feel distressed?

- ○ Yes
- ○ No

Helping the confused older person

If something in these surveys caused you to feel distressed or upset then we advise you to talk to a supportive person about these feelings. You might wish to talk to someone using the relevant helpline listed below.

Australia
National Dementia Helpline
1800 100 500

New Zealand
Local Alzheimers New Zealand organisations
0800 004 001

Canada
Alzheimer Society of B.C. Dementia Helpline
Province-wide: 1-800-936-6033
Lower Mainland: 604-681-8651

Ireland
Alzheimer National Helpline
1800 341 341

United Kingdom
National Dementia Helpline
0300 222 1122

United States
Alzheimer's Association 24/7 Helpline
1.800.272.3900

That is the end of the first round survey!

Thank you very much for your contribution.

By pressing the "next" button your final responses will be registered with our survey software. Once all panel members have lodged their responses, we will collate the data and send you a report on the findings and the second survey.

We are extremely grateful for your contribution.

Best Wishes,
The Centre for Mental Health, University of Melbourne and Mental Health First Aid Australia Research Team

Helping the confused older person - Round 2

INTRODUCTION

How this questionnaire was developed

The statements in this questionnaire were derived from the results of the Round 1 survey. You will note that each statement is marked as either a new or rerate item. New items were derived from the comments provided in the first survey. An item is rerated when 80% or more of the panel members from one of the groups rated it as essential or important **AND** if 70%–79% of panel members from the other panel rated it as essential or important.

Instructions

Please complete the questionnaire by rating each statement **according to how important you believe it is for inclusion in the guidelines** for helping a confused older person. Please keep in mind that the guidelines will be used by the general public and as such, the statements need to be rated according to how important each one is for someone, who does not necessarily have a counselling or clinical background, to help an older person with confusion.

This questionnaire should take approximately 60 minutes to complete. You can complete the survey in two or more sittings. Your answers are saved when you click *'Next'* at the bottom of a page. This marks your page and you can begin again at a later date on the next page. **Please be aware that once you have logged on and started responding you must complete the questionnaire on the same computer.**

* 1. Please provide your name so I can verify who is eligible to complete Round 3.

* 2.
Area of expertise

○ Carer advocate

○ Professional

Helping the confused older person - Round 2

Overview of Questionnaire

The questionnaire is divided into the following sections:

1. General awareness

2. Memory and other cognitive problems

3. Seeking help

4. Encountering someone wandering

5. Communication

6. Challenges you may encounter during communication

7. Discussing sensitive issues and making decisions with the person

8. Challenging behaviours

9. Delirium

Helping the confused older person - Round 2

Definitions

These terms may have different meanings for participants. The definitions below are how these terms are used in this survey. When completing the survey, please ensure you use these definitions only.

Person in this survey refers to an **older person who is experiencing confusion** which may be due to dementia, delirium or other conditions. Older person in this survey refers to those aged 65 or older, however it is expected that the resulting guidelines may also be relevant to assisting adults with confusion who are younger.

Mental health first aid for the confused older person is the help offered to a person who may be developing dementia or delirium, is experiencing a worsening of existing dementia symptoms or is in a crisis due to their confusion.

Helper in this survey refers to the individual who provides mental health first aid as defined above. **The helper may be a family member, friend, neighbour, concerned community member or a paid carer without specialist qualifications.**

Confusion in this survey is a broad term that refers to a decline in normal cognitive ability, which may vary from mild to severe. The cognitive changes may be associated with dementia or delirium. It may include a number of the following signs and symptoms: lack of alertness, poor attention span, disorientation to time and place, trouble following a conversation, unclear or illogical speech, impaired short-term memory, difficulty in planning and carrying out tasks, inappropriate behaviour, disconnection from reality or delusional beliefs.

Dementia is a condition involving progressive decline of cognitive abilities such as short-term memory, language and the ability to plan and carry out tasks. Dementia is an umbrella term for a large group of illnesses that cause this progressive decline. The symptoms appear over months and years. The initial signs of confusion may be mild, or may be only apparent at certain times or in certain situations, but the condition tends to get worse over time.

Delirium is a condition where a sudden and obvious worsening of a person's usual level of functioning appears over hours or days. Delirium can involve problems with attention, awareness, orientation to environment and other areas of cognitive functioning. Delirium is caused by an underlying disease or environmental factors, such as medication.

Helping the confused older person - Round 2

General awareness

This section asks you about what general information the helper should know about confusion, dementia and delirium. Please rate how important (from 'essential' to 'should not be included') you think it is that each statement be included in the guidelines.

When providing first aid to the confused person, the helper will need to use their judgement because a person's decline in cognitive ability may be very mild or more severe. Please keep this in mind when rating the following items.

Please also keep the **definitions** in mind when rating the statements.

* 3. The helper should be aware that signs and symptoms of dementia can differ from person to person. (New)

Essential	Important	Don't know/Depends	Unimportant	Should not be included
○	○	○	○	○

* 4. The helper should be aware that early detection of dementia has benefits for the family's acceptance and long-term management of the condition. (New)

Essential	Important	Don't know/Depends	Unimportant	Should not be included
○	○	○	○	○

Helping the confused older person - Round 2

Memory and other cognitive problems

This section asks you what information the helper should know about memory and other cognitive problems. It also asks how the helper can assist the person with these limitations. Please rate how important (from 'essential' to 'should not be included') you think it is that each statement be included in the guidelines.

When providing first aid to the confused person, the helper will need to use their judgement because a person's decline in cognitive ability may be very mild or more severe. Please keep this in mind when rating the following items.

Please also keep the **definitions** in mind when rating the statements.

Understanding changes in memory

* 5. The helper should not assume that the person cannot learn and enjoy new things. (New)

Essential	Important	Don't know/Depends	Unimportant	Should not be included
○	○	○	○	○

* 6. If the person is going to be in an unfamiliar place, the helper should ensure that the person has some familiar people with them to reduce the chance of confusion. (New)

Essential	Important	Don't know/Depends	Unimportant	Should not be included
○	○	○	○	○

Helping the confused older person - Round 2

Memory and other cognitive problems (continued)

This section asks you what information the helper should know about memory and other cognitive problems. It also asks how the helper can assist the person with these limitations. Please rate how important (from ‘essential’ to ‘should not be included’) you think it is that each statement be included in the guidelines.

When providing first aid to the confused person, the helper will need to use their judgement because a person's decline in cognitive ability may be very mild or more severe. Please keep this in mind when rating the following items.

Please also keep the **definitions** in mind when rating the statements.

Completing tasks and making decisions

* 7. The helper should allow time for the person to complete a task, because they may take longer than they used to. (New)

Essential	Important	Don't know/Depends	Unimportant	Should not be included
○	○	○	○	○

* 8. If the person appears to be frustrated by a task, the helper should divert their attention before the stress becomes overwhelming. (Rerate)

Essential	Important	Don't know/Depends	Unimportant	Should not be included
○	○	○	○	○

* 9. The helper should allow the person to make whatever decisions they are capable of making, as long as these do not pose a risk of harm to the person or others. (New)

Essential	Important	Don't know/Depends	Unimportant	Should not be included
○	○	○	○	○

* 10. When the person needs to make a decision, the helper can assist by limiting any choice to two options. (Rerate)

Essential	Important	Don't know/Depends	Unimportant	Should not be included
○	○	○	○	○

* 11. When the person needs to make a decision, the helper can assist by limiting any choice to a small number of options. (New)

Essential	Important	Don't know/Depends	Unimportant	Should not be included
○	○	○	○	○

* 12. When the person needs to make a decision, the helper can assist by limiting any choice to options that are safe and mutually beneficial. (New)

Essential	Important	Don't know/Depends	Unimportant	Should not be included
○	○	○	○	○

* 13. If the person has memory problems, the helper should be aware of a range of strategies they can use to assist the person. (New)

Essential	Important	Don't know/Depends	Unimportant	Should not be included
○	○	○	○	○

Helping the confused older person - Round 2

Memory and other cognitive problems (cont.)

This section asks you what information the helper should know about memory and other cognitive problems. It also asks how the helper can assist the person with these limitations. Please rate how important (from 'essential' to 'should not be included') you think it is that each statement be included in the guidelines.

When providing first aid to the confused person, the helper will need to use their judgement because a person's decline in cognitive ability may be very mild or more severe. Please keep this in mind when rating the following items.

Please also keep the **definitions** in mind when rating the statements.

Completing tasks and making decisions (cont.)

* 14. If the person has forgotten that they have done something and keeps asking to do it (e.g. attending a doctor's appointment), the helper should consider using the following strategies:

	Essential	Important	Don't know/Depends	Unimportant	Should not be included
distracting the person. (New)	○	○	○	○	○
acknowledging the emotions behind the person's concerns. (New)	○	○	○	○	○
showing them the evidence that they have done it. (New)	○	○	○	○	○
repeating the answer, in a kind and reassuring tone of voice. (New)	○	○	○	○	○
patiently accepting the repetition, if the above strategies do not help. (New)	○	○	○	○	○

* 15. If the person does not recognise what an object is for, the helper should consider using the following strategies:

	Essential	Important	Don't know/Depends	Unimportant	Should not be included
acknowledging any frustration the person may be experiencing. (New)	○	○	○	○	○
explaining what it is for in a sensitive manner to avoid the person feeling a sense of failure (New)	○	○	○	○	○
using step-by-step prompts. (New)	○	○	○	○	○
demonstrating use of the item. (New)	○	○	○	○	○
using hand-over-hand guidance. (New)	○	○	○	○	○
miming use of the object. (New)	○	○	○	○	○

* 16. If the person is struggling with a task (e.g. dressing), the helper should consider using the following strategies:

	Essential	Important	Don't know/Depends	Unimportant	Should not be included
acknowledging the person's frustration. (New)	○	○	○	○	○
distracting them for a few minutes and then helping them start again. (New)	○	○	○	○	○
explaining how to do it in a sensitive manner to avoid the person feeling a sense of failure. (New)	○	○	○	○	○
using step-by-step prompts. (New)	○	○	○	○	○
demonstrating how to do it. (New)	○	○	○	○	○
using hand-over-hand guidance. (New)	○	○	○	○	○
miming how to do it (New)	○	○	○	○	○
help the person at the point at which they have become stuck. (New)	○	○	○	○	○
intervening and completing the task for them (New)	○	○	○	○	○

* 17. If the person is struggling with a task (e.g. dressing), the helper should intervene **only** when the person indicates that they want help. (New)

Essential	Important	Don't know/Depends	Unimportant	Should not be included
○	○	○	○	○

* 18. The helper should be aware that assistance means doing something 'with' the person, not doing 'for' them, so as to maintain as much of the person's independence as possible. (New)

Essential	Important	Don't know/Depends	Unimportant	Should not be included
○	○	○	○	○

* 19. If the person talks about deceased people as though they are alive (e.g. a parent who has died long ago) and they are distressed, the helper should consider using the following strategies:

	Essential	Important	Don't know/Depends	Unimportant	Should not be included
gently explain that the person has passed away. (New)	◯	◯	◯	◯	◯
do not correct them. (New)	◯	◯	◯	◯	◯
engage in a conversation about the deceased person. (New)	◯	◯	◯	◯	◯
try to understand why they are talking about the person, in case it references a need that can be addressed. (New)	◯	◯	◯	◯	◯

* 20. If the person talks about deceased people as though they are alive (e.g. a parent who has died long ago) and they are **not** distressed, the helper should consider using the following strategies:

	Essential	Important	Don't know/Depends	Unimportant	Should not be included
gently explain that the person has passed away. (New)	◯	◯	◯	◯	◯
do not correct them. (New)	◯	◯	◯	◯	◯
engage in a conversation about the deceased person. (New)	◯	◯	◯	◯	◯
try to understand why they are talking about the person, in case it references a need that can be addressed. (New)	◯	◯	◯	◯	◯

Helping the confused older person - Round 2

Seeking help

This section asks you what information the helper should know about when and how the helper can assist the person to get professional help. Please rate how important (from ‘essential’ to ‘should not be included’) you think it is that each statement be included in the guidelines.

When providing first aid to the confused person, the helper will need to use their judgement because a person's decline in cognitive ability may be very mild or more severe. Please keep this in mind when rating the following items.

Please also keep the **definitions** in mind when rating the statements.

Raising the topic of seeking help for memory problems

* 21. If the helper is worried about the person's memory, before raising this with them, the helper should consult with someone experienced in this type of conversation. (New)

Essential	Important	Don't know/Depends	Unimportant	Should not be included
○	○	○	○	○

* 22. During a conversation about the person's memory and functioning, the helper should:

	Essential	Important	Don't know/ depends	Unimportant	Should not be included
explain that they are concerned because they care. (Rerate)	○	○	○	○	○
make a plan with the person to see a doctor. (Rerate)	○	○	○	○	○
reassure them that they care for or love the person regardless of their memory and functioning, as this will provide a sense of security for the person. (New)	○	○	○	○	○

* 23. The helper should encourage the person to see a doctor if the person has any signs or symptoms of dementia. (Rerate)

Essential	Important	Don't know/Depends	Unimportant	Should not be included
○	○	○	○	○

Helping the confused older person - Round 2

Seeking help (continued)

This section asks you what information the helper should know about when and how the helper can assist the person to get professional help. Please rate how important (from ‘essential’ to ‘should not be included’) you think it is that each statement be included in the guidelines.

When providing first aid to the confused person, the helper will need to use their judgement because a person's decline in cognitive ability may be very mild or more severe. Please keep this in mind when rating the following items.

Please also keep the **definitions** in mind when rating the statements.

If the person does not want to seek help

* 24. If the person is reluctant to get help, the helper should:

	Essential	Important	Don't know/ depends	Unimportant	Should not be included
discuss the matter with the person and try to identify the reason behind their reluctance. (Rerate)	○	○	○	○	○
suggest that the person go for a general medical check-up. (Rerate)	○	○	○	○	○
tell the person that a doctor's visit will help to rule out any physical or temporary conditions causing their signs or symptoms. (New)	○	○	○	○	○
call a dementia or Alzheimer's helpline for assistance. (Rerate)	○	○	○	○	○
find out if there are options for house calls by health professionals who specialise in dementia. (Rerate)	○	○	○	○	○
make an appointment with the person's doctor for the helper to discuss their concerns without the person present. (New)	○	○	○	○	○
identify another individual who has a good relationship with the person to support them to seek help. (New)	○	○	○	○	○
suggest to the person that the helper will monitor the person's memory problems for a month and then revisit the topic of seeking help. (New)	○	○	○	○	○
respect the person's wishes. (New)	○	○	○	○	○

* 25. If the person gets distressed by a conversation about seeking professional help, the helper should stop the conversation. (New)

Essential	Important	Don't know/Depends	Unimportant	Should not be included
○	○	○	○	○

* 26. If the person refuses to get help and their health or safety is at risk, the helper should get aged care assessment services involved. (Rerate)

Essential	Important	Don't know/Depends	Unimportant	Should not be included
○	○	○	○	○

Helping the confused older person - Round 2

Seeking help (continued)

This section asks you what information the helper should know about when and how the helper can assist the person to get professional help. Please rate how important (from 'essential' to 'should not be included') you think it is that each statement be included in the guidelines.

When providing first aid to the confused person, the helper will need to use their judgement because a person's decline in cognitive ability may be very mild or more severe. Please keep this in mind when rating the following items.

Please also keep the **definitions** in mind when rating the statements.

Professional and other supports

* 27. In order to act as an advocate for the person, the helper should go with them to any doctor or health professional appointments. (New)

Essential	Important	Don't know/Depends	Unimportant	Should not be included
○	○	○	○	○

* 28. If the helper attends the doctor's appointment with the person, the helper should ask the person's permission to be given confidential information. (Rerate)

Essential	Important	Don't know/Depends	Unimportant	Should not be included
○	○	○	○	○

* 29. If the helper attends a doctor's appointment with the person, the helper should directly ask the doctor for a referral to a dementia specialist. (New)

Essential	Important	Don't know/Depends	Unimportant	Should not be included
○	○	○	○	○

* 30. The helper should find out about support groups for the person with early stage dementia and their family and friends. (Rerate)

Essential	Important	Don't know/Depends	Unimportant	Should not be included
○	○	○	○	○

Helping the confused older person - Round 2

Encountering someone who is wandering

This section asks you what information the helper should know in order to assist a person who is wandering.

Wandering *is a dementia-related behaviour that sees a disoriented individual move about, sometimes with repetitive pacing or lapping in one area, and other times leaving their usual environment. A wandering person may become lost, leave a safe environment or intrude in inappropriate places.*

Please rate how important (from ‘essential’ to ‘should not be included’) you think it is that each statement be included in the guidelines.

When providing first aid to the confused person, the helper will need to use their judgement because a person's decline in cognitive ability may be very mild or more severe. Please keep this in mind when rating the following items.

Please also keep the **definitions** in mind when rating the statements.

* 31. If the helper encounters a person who is wandering, the helper should:

	Essential	Important	Don't know/ depends	Unimportant	Should not be included
advise the person if the helper plans to contact the person's family member or a friend. (Rerate)	○	○	○	○	○
check if the person has some form of tracking device that they could use to contact the person's home or carer. (New)	○	○	○	○	○

* 32. If the helper encounters a person who is wandering, and the helper cannot find out any of the individual's emergency contact information, the helper should:

	Essential	Important	Don't know/Depends	Unimportant	Should not be included
suggest that together they call the police for help. (Rerate)	○	○	○	○	○
suggest that they sit and have a chat together, while the helper quietly calls emergency services. (New)	○	○	○	○	○

* 33. The helper should not leave the person who is wandering alone, even if the person declines their help. (New)

Essential	Important	Don't know/Depends	Unimportant	Should not be included
○	○	○	○	○

* 34. The helper should not leave the person who is wandering**and distressed** alone, even if the person declines their help. (New)

Essential	Important	Don't know/Depends	Unimportant	Should not be included
○	○	○	○	○

* 35. If the helper encounters a person who is wandering, and the helper is very worried about the person's health or safety, the helper should call emergency services. (New)

Essential	Important	Don't know/Depends	Unimportant	Should not be included
○	○	○	○	○

* 36. If the person gets upset at an offer to contact emergency services, the helper should ask someone in the vicinity to make the call. (New)

Essential	Important	Don't know/Depends	Unimportant	Should not be included
○	○	○	○	○

* 37. When emergency services are called, the helper and emergency services officer should sit and talk quietly with the person to see how they can help. (New)

Essential	Important	Don't know/Depends	Unimportant	Should not be included
○	○	○	○	○

* 38. The helper should attempt to provide assistance to the person who is wandering without putting themselves in danger. (New)

Essential	Important	Don't know/Depends	Unimportant	Should not be included
○	○	○	○	○

Helping the confused older person - Round 2

Communication

This section asks you what information the helper should know in order to communicate effectively with a confused person. Please rate how important (from ‘essential’ to ‘should not be included’) you think it is that each statement be included in the guidelines.

When providing first aid to the confused person, the helper will need to use their judgement because a person's decline in cognitive ability may be very mild or more severe. Please keep this in mind when rating the following items.

Please also keep the **definitions** in mind when rating the statements.

Getting and retaining attention

* 39. To gain the complete attention of the person, the helper should join in on the person's activity. (New)

Essential	Important	Don't know/Depends	Unimportant	Should not be included
○	○	○	○	○

* 40. If the helper cannot get the person's attention, they should wait a few minutes and try again. (Rerate)

Essential	Important	Don't know/Depends	Unimportant	Should not be included
○	○	○	○	○

* 41. If the helper cannot get the person's attention and the conversation is not important, they should leave it for another time. (New)

Essential	Important	Don't know/Depends	Unimportant	Should not be included
○	○	○	○	○

* 42. If the person does not appear to notice the helper, the helper should position themselves so the person can look down at them, e.g. if the person is seated the helper could crouch down in front of their chair. (Rerate)

Essential	Important	Don't know/Depends	Unimportant	Should not be included
○	○	○	○	○

* 43. Unless necessary, the helper should not interrupt the person in the middle of a task or conversation, as this may confuse them. (New)

Essential	Important	Don't know/Depends	Unimportant	Should not be included
○	○	○	○	○

Helping the confused older person - Round 2

Communication (continued)

This section asks you what information the helper should know in order to communicate effectively with a confused person. Please rate how important (from ‘essential’ to ‘should not be included’) you think it is that each statement be included in the guidelines.

When providing first aid to the confused person, the helper will need to use their judgement because a person's decline in cognitive ability may be very mild or more severe. Please keep this in mind when rating the following items.

Please also keep the **definitions** in mind when rating the statements.

Where the person doesn't consistently recognise the people in their life

* 44. The helper should reintroduce themselves each time they approach the person because the person may forget the names of people and their relationships to them. (Rerate)

Essential	Important	Don't know/Depends	Unimportant	Should not be included
○	○	○	○	○

Helping the confused older person - Round 2

Communication (continued)

This section asks you what information the helper should know in order to communicate effectively with a confused person. Please rate how important (from ‘essential’ to ‘should not be included’) you think it is that each statement be included in the guidelines.

When providing first aid to the confused person, the helper will need to use their judgement because a person's decline in cognitive ability may be very mild or more severe. Please keep this in mind when rating the following items.

Please also keep the **definitions** in mind when rating the statements.

Manner of communication

* 45. When talking to a person the helper should:

	Essential	Important	Don't know/Depends	Unimportant	Should not be included
use short sentences. (Rerate)	◯	◯	◯	◯	◯
avoid using expressions that can be taken too literally, e.g. "shake a leg". (Rerate)	◯	◯	◯	◯	◯
tailor their use of language and vocabulary to be similar to the person's own. (New)	◯	◯	◯	◯	◯
adjust the pace of their speech depending on how well the person appears to be understanding them. (New)	◯	◯	◯	◯	◯
speak at their normal pace, because speaking slower may come across as condescending and patronising. (New)	◯	◯	◯	◯	◯
avoid standing too close to the person whilst talking as this can be intimidating. (Rerate)	◯	◯	◯	◯	◯
stay still whilst talking to the person as this will make it easier for the person to follow what you are saying. (Rerate)	◯	◯	◯	◯	◯

* 46. The helper should be aware that talking with the person may require skilled communication and they should be prepared to accept that they may make mistakes. (New)

Essential	Important	Don't know/Depends	Unimportant	Should not be included
◯	◯	◯	◯	◯

* 47. The helper should not use phrases such as, "do you remember?", "don't you remember?" and "have you forgotten?", because it may upset the person and is unlikely to be helpful. (Rerate)

Essential	Important	Don't know/Depends	Unimportant	Should not be included
◯	◯	◯	◯	◯

* 48. The helper should avoid 'why' questions which may put the person on the defensive, e.g. "Why did you do that?" (Rerate)

Essential	Important	Don't know/Depends	Unimportant	Should not be included
○	○	○	○	○

* 49. The helper should not ask questions that require a lot of thought or memory, e.g. don't ask, "What did you do today?" Instead, shape the question to address current feelings: "Are you having a good day?". (Rerate)

Essential	Important	Don't know/Depends	Unimportant	Should not be included
○	○	○	○	○

* 50. The helper should avoid testing the person's memory because this can be embarrassing and hurtful. (Rerate)

Essential	Important	Don't know/Depends	Unimportant	Should not be included
○	○	○	○	○

* 51. If the helper has repeated a question using exactly the same words and the person still does not respond, the helper should try to get the message across in a different way. (Rerate)

Essential	Important	Don't know/Depends	Unimportant	Should not be included
○	○	○	○	○

* 52. The helper should provide validation and acknowledgement to the person by reflecting back what the person has said. (Rerate)

Essential	Important	Don't know/Depends	Unimportant	Should not be included
○	○	○	○	○

* 53. After the person responds to a question, the helper should paraphrase what the person said to show them they have understood. (New)

Essential	Important	Don't know/Depends	Unimportant	Should not be included
○	○	○	○	○

* 54. The helper should continue sharing their day-to-day thoughts and feelings with the person, as it lets the person know that they are valued. (Rerate)

Essential	Important	Don't know/Depends	Unimportant	Should not be included
○	○	○	○	○

Helping the confused older person - Round 2

Communication (continued)

This section asks you what information the helper should know in order to communicate effectively with a confused person. Please rate how important (from ‘essential’ to ‘should not be included’) you think it is that each statement be included in the guidelines.

When providing first aid to the confused person, the helper will need to use their judgement because a person's decline in cognitive ability may be very mild or more severe. Please keep this in mind when rating the following items.

Please also keep the **definitions** in mind when rating the statements.

Advocating for the person when they are communicating with others

* 55. In a group situation where a one-on-one conversation with the person is not possible, the helper should try to ensure that only one person speaks at a time. (Rerate)

Essential	Important	Don't know/Depends	Unimportant	Should not be included
○	○	○	○	○

* 56. The helper should remind others that they should avoid talking about the person when the person is within hearing range. (Rerate)

Essential	Important	Don't know/Depends	Unimportant	Should not be included
○	○	○	○	○

* 57. If the helper observes someone using baby-talk with the person, the helper should say, "I'm sorry, but (name) relates best when you talk to them in a normal adult manner." (Rerate)

Essential	Important	Don't know/Depends	Unimportant	Should not be included
○	○	○	○	○

* 58. If the helper observes someone using baby-talk with the person, the helper should take them aside and tell them they need to talk with the person in an adult manner. (New)

Essential	Important	Don't know/Depends	Unimportant	Should not be included
○	○	○	○	○

* 59. Where possible, the helper should prepare others before they visit with the person so they know what to expect and how to communicate with the person. (New)

Essential	Important	Don't know/Depends	Unimportant	Should not be included
○	○	○	○	○

Helping the confused older person - Round 2

Non-verbal communication

This section asks you what information the helper should know in order to communicate effectively with a confused person. Please rate how important (from ‘essential’ to ‘should not be included’) you think it is that each statement be included in the guidelines.

When providing first aid to the confused person, the helper will need to use their judgement because a person's decline in cognitive ability may be very mild or more severe. Please keep this in mind when rating the following items.

Please also keep the **definitions** in mind when rating the statements.

* 60. The helper should be aware that communicating with the person using body language may be more effective than spoken words. (Rerate)

Essential	Important	Don't know/Depends	Unimportant	Should not be included
○	○	○	○	○

* 61. The helper should observe the person's breathing to gauge how they are feeling, e.g. faster breathing may indicate distress, while slower breathing may show they are calm. (Rerate)

Essential	Important	Don't know/Depends	Unimportant	Should not be included
○	○	○	○	○

* 62. The helper should try to keep their facial expressions and gestures positive. (Rerate)

Essential	Important	Don't know/Depends	Unimportant	Should not be included
○	○	○	○	○

* 63. The helper should use facial expressions to communicate meanings to the person because these may be more readily understood. (Rerate)

Essential	Important	Don't know/Depends	Unimportant	Should not be included
○	○	○	○	○

* 64. The helper should be aware that a gentle touch of the arm or hand can communicate to the person that the helper is interested and really cares. (Rerate)

Essential	Important	Don't know/Depends	Unimportant	Should not be included
○	○	○	○	○

Helping the confused older person - Round 2

Challenges you may encounter during communication

This section asks you what information the helper should know in order to respond to challenges that may occur whilst communicating with a confused person. Please rate how important (from ‘essential’ to ‘should not be included’) you think it is that each statement be included in the guidelines.

When providing first aid to the confused person, the helper will need to use their judgement because a person's decline in cognitive ability may be very mild or more severe. Please keep this in mind when rating the following items.

Please also keep the **definitions** in mind when rating the statements.

* 65. The helper should try to be patient when assisting the person. (New)

Essential	Important	Don't know/Depends	Unimportant	Should not be included
○	○	○	○	○

* 66. The helper should be aware that sometimes the best thing they can do is remain silent and let the person find their own way to cope with difficulties in conversation, e.g. finding a word to use. (Rerate)

Essential	Important	Don't know/Depends	Unimportant	Should not be included
○	○	○	○	○

When the helper doesn't understand what the person is saying

* 67. If the person's words are mixed up and seem nonsensical, the helper should smile and say, "I am having a problem understanding". This implies that the person is not the problem. (Rerate)

Essential	Important	Don't know/Depends	Unimportant	Should not be included
○	○	○	○	○

When the person can't find the right words

* 68. If the person is unable to find the right word, but the helper has understood what they are trying to say, the helper should clarify the message to make sure they have understood. (New)

Essential	Important	Don't know/Depends	Unimportant	Should not be included
○	○	○	○	○

* 69. If the person uses the wrong word, the helper should not point out the error but repeat back the person's message using the correct word. (New)

Essential	Important	Don't know/Depends	Unimportant	Should not be included
○	○	○	○	○

Helping the confused older person - Round 2

Challenges you may encounter during communication (continued)

This section asks you what information the helper should know in order to respond to challenges that may occur whilst communicating with a confused person. Please rate how important (from ‘essential’ to ‘should not be included’) you think it is that each statement be included in the guidelines.

When providing first aid to the confused person, the helper will need to use their judgement because a person's decline in cognitive ability may be very mild or more severe. Please keep this in mind when rating the following items.

Please also keep the **definitions** in mind when rating the statements.

Problems with comprehension

* 70. The helper should ask the person for feedback on whether the person has understood them and look for signs of understanding, e.g. the person's body language and facial expression. (Rerate)

Essential	Important	Don't know/Depends	Unimportant	Should not be included
○	○	○	○	○

* 71. The helper should ask the person for feedback on whether the person has understood them. (New)

Essential	Important	Don't know/Depends	Unimportant	Should not be included
○	○	○	○	○

* 72. The helper should look for signs of understanding, e.g. the person's body language and facial expression. (New)

Essential	Important	Don't know/Depends	Unimportant	Should not be included
○	○	○	○	○

* 73. If the person doesn't understand, the helper should resist the urge to talk louder as this will upset the person and make communication more difficult. (Rerate)

Essential	Important	Don't know/Depends	Unimportant	Should not be included
○	○	○	○	○

* 74. If the helper does not understand what the person is saying then the helper should guess and respond accordingly. (New)

Essential	Important	Don't know/Depends	Unimportant	Should not be included
○	○	○	○	○

Encountering resistance

* 75. Rather than asking questions that are likely to trigger immediate resistance, the helper should tell the person what is going to happen, e.g. rather than asking "Do you want to have your shower now?", the helper should say "It's time for your shower now". (Rerate)

Essential	Important	Don't know/Depends	Unimportant	Should not be included
○	○	○	○	○

* 76. Rather than asking questions that are likely to trigger immediate resistance (e.g. "Do you want to take a shower), the helper should break the task down into steps and use statements rather than questions, e.g. 1. "I will give you a hand to the bathroom", 2. "put your hand under the water and tell me if it is the right temperature", etc. (New)

Essential	Important	Don't know/Depends	Unimportant	Should not be included
○	○	○	○	○

* 77. Rather than just telling the person what is about to happen, e.g. "It's time for your shower now", the helper should try to give a reason that it needs to occur, e.g. "You need to have your shower now, so you are clean and ready for breakfast." (New)

Essential	Important	Don't know/Depends	Unimportant	Should not be included
○	○	○	○	○

Arguments

* 78. The helper should be aware that arguments may develop from the person's frustration and are not a reflection on the helper. (New)

Essential	Important	Don't know/Depends	Unimportant	Should not be included
○	○	○	○	○

* 79. If an argument develops **over a minor matter**, the helper should:

	Essential	Important	Don't know/Depends	Unimportant	Should not be included
acknowledge the person's feelings and frustrations. (New)	○	○	○	○	○
apologise and then let the matter be. (New)	○	○	○	○	○
change the topic of conversation or begin a new activity. (New)	○	○	○	○	○
agree with the person and then try to change the subject. (New)	○	○	○	○	○

* 80. If an argument develops, the helper should:

	Essential	Important	Don't know/Depends	Unimportant	Should not be included
acknowledge the person's feelings and frustrations. (New)	○	○	○	○	○
apologise and then let the matter be. (New)	○	○	○	○	○
change the topic of conversation or begin a new activity. (New)	○	○	○	○	○
agree with the person and then try to change the subject. (New)	○	○	○	○	○

Helping the confused older person - Round 2

Discussing sensitive issues and making decisions with the person

This section concerns conversations about sensitive issues which might include when to see a doctor about symptoms of dementia, stopping driving or the need for a change in level of care or living arrangements. In this section, 'a conversation' refers to a conversation about a sensitive issue. Please rate how important (from ‘essential’ to ‘should not be included’) you think it is that each statement be included in the guidelines.

When providing first aid to the confused person, the helper will need to use their judgement because a person's decline in cognitive ability may be very mild or more severe. Please keep this in mind when rating the following items.

Please also keep the **definitions** in mind when rating the statements.

How to discuss sensitive issues

* 81. When discussing a sensitive topic, the helper should:

	Essential	Important	Don't know/Depends	Unimportant	Should not be included
choose a place familiar to the person as a setting for a conversation because this is likely to help facilitate communication and minimise anxiety. (Rerate)	◯	◯	◯	◯	◯
begin the conversation with neutral topics to help build trust and help the person feel relaxed, e.g. talking about the weather, family. (Rerate)	◯	◯	◯	◯	◯
attempt to remain calm and objective. (New)	◯	◯	◯	◯	◯
consider enlisting the help of another person who can remain calm and objective. (New)	◯	◯	◯	◯	◯

* 82. The helper should be aware that there is no need to be embarrassed at displays of negative emotion by the person (e.g. tearful, angry), because it is good for the person to express their emotions. (Rerate)

Essential	Important	Don't know/Depends	Unimportant	Should not be included
◯	◯	◯	◯	◯

* 83. If the person shows negative emotions (e.g. tearful, angry), the helper should take them somewhere private where the person can express this without feeling embarrassed. (New)

Essential	Important	Don't know/Depends	Unimportant	Should not be included
◯	◯	◯	◯	◯

* 84. When discussing topics which evoke a strong emotional response from the person, the helper should validate and acknowledge how the person is feeling, e.g. "I can see this is upsetting, that's very understandable." (New)

Essential	Important	Don't know/Depends	Unimportant	Should not be included
◯	◯	◯	◯	◯

Discussing the diagnosis

* 85. The helper should be aware that people with dementia usually want to know the truth about their diagnosis. (New)

Essential	Important	Don't know/Depends	Unimportant	Should not be included
◯	◯	◯	◯	◯

* 86. The helper should be aware that knowing the diagnosis can enable the person to take an active role in planning for their future. (Rerate)

Essential	Important	Don't know/Depends	Unimportant	Should not be included
◯	◯	◯	◯	◯

* 87. If the person does not understand all that their diagnosis means or they deny it, the helper should accept their reaction. (New)

Essential	Important	Don't know/Depends	Unimportant	Should not be included
◯	◯	◯	◯	◯

* 88. The helper should choose how they explain to the person what is happening based on the person's ability to understand, e.g. A straightforward explanation versus a more gentle approach, "You have a memory problem". (Rerate)

Essential	Important	Don't know/Depends	Unimportant	Should not be included
◯	◯	◯	◯	◯

* 89. Whilst discussing the diagnosis, the helper should reassure the person that there are things they can do themselves that can support their memory and maintain their independence. (New)

Essential	Important	Don't know/Depends	Unimportant	Should not be included
◯	◯	◯	◯	◯

Helping the confused older person - Round 2

Discussing sensitive issues and making decisions with the person (continued)

This section concerns conversations about sensitive issues which might include when to see a doctor about symptoms of dementia, stopping driving or the need for a change in level of care or living arrangements. In this section, 'a conversation' refers to a conversation about a sensitive issue. Please rate how important (from 'essential' to 'should not be included') you think it is that each statement be included in the guidelines.

When providing first aid to the confused person, the helper will need to use their judgement because a person's decline in cognitive ability may be very mild or more severe. Please keep this in mind when rating the following items.

Please also keep the **definitions** in mind when rating the statements.

Discussing plans for the future

An advance directive is a document describing how the person wants to be treated when they are unable to make their own decisions due to their present state of illness. Advance directives should specify the circumstances under which they will be used and who will take the various actions specified, e.g. when the person is becoming unwell or when they are acutely unwell.

A power of attorney is where another person is appointed to carry out specified or all legal or financial matters for someone.

* 90. The helper should arrange times with the person to discuss the person's wishes, concerns and any preferences for the future. (Rerate)

Essential	Important	Don't know/Depends	Unimportant	Should not be included
○	○	○	○	○

* 91. If the person has concerns about facing the future, the helper should reassure the person about the advantages of planning, e.g. "Don't be scared of planning ahead. It will make life much easier in the future." (Rerate)

Essential	Important	Don't know/Depends	Unimportant	Should not be included
○	○	○	○	○

* 92. If the person does not have an advance care directive on relevant topics (e.g. care preferences, management of finances and arrangements after death), the helper should encourage or assist them to make an appointment with an appropriate professional to develop one. (New)

Essential	Important	Don't know/Depends	Unimportant	Should not be included
○	○	○	○	○

* 93. If the person has not arranged a power of attorney, the helper should encourage or assist them to make these arrangements with an appropriate professional. (New)

Essential	Important	Don't know/Depends	Unimportant	Should not be included
○	○	○	○	○

* 94. The helper should allow the person to make whatever decisions they are capable of making, as long as these do not involve danger to the person or to others. (New)

Essential	Important	Don't know/Depends	Unimportant	Should not be included
○	○	○	○	○

Discussing driving and making decisions with the person

This section concerns conversations and decisions about stopping or restricting driving. In this section, the helper will generally be a family caregiver. Please rate how important (from 'essential' to 'should not be included') you think it is that each statement be included in the guidelines.

When providing first aid to the confused person, the helper will need to use their judgement because a person's decline in cognitive ability may be very mild or more severe. Please keep this in mind when rating the following items.

Please also keep the **definitions** in mind when rating the statements.

Discussing and planning for future driving decisions

* 95. The helper should be aware that an early diagnosis and management may help the person with dementia drive for longer. (New)

Essential	Important	Don't know/Depends	Unimportant	Should not be included
○	○	○	○	○

* 96. The helper should talk to the person about driving issues, e.g. safety and liability. (Rerate)

Essential	Important	Don't know/Depends	Unimportant	Should not be included
○	○	○	○	○

* 97. The helper should initiate a discussion about driving in a way that will not lead the person to be defensive about their abilities, e.g. Rather than saying, "Your driving is terrible, you are getting lost, and you're just not safe", the helper should say "You are getting absent-minded about stop lights." (Rerate)

Essential	Important	Don't know/Depends	Unimportant	Should not be included
○	○	○	○	○

* 98. The helper should initiate a discussion about driving in a way that is**less likely** to lead the person to be defensive about their abilities, e.g. Rather than saying, "Your driving is terrible, you are getting lost, and you're just not safe", the helper should say "I am concerned about your safety, how are you feeling about your driving?" (New)

Essential	Important	Don't know/Depends	Unimportant	Should not be included
○	○	○	○	○

* 99. When discussing driving restrictions, the helper should focus on the nature of the disease, i.e. that many people with dementia have safe past driving records, but this does not mean they will be safe as a driver with dementia in the future. (Rerate)

Essential	Important	Don't know/Depends	Unimportant	Should not be included
○	○	○	○	○

* 100. The helper should communicate with the person about their driving early, because this can help the person decide on a course of action before an accident occurs. (New)

Essential	Important	Don't know/Depends	Unimportant	Should not be included
○	○	○	○	○

* 101. Shortly after diagnosis, the helper should begin discussing and planning**with the person** about when they should stop driving. (New)

Essential	Important	Don't know/Depends	Unimportant	Should not be included
○	○	○	○	○

* 102. Shortly after diagnosis, the helper should begin discussing and planning**with the person's family** about when the person should stop driving. (New)

Essential	Important	Don't know/Depends	Unimportant	Should not be included
○	○	○	○	○

* 103. The helper should help the person put a written agreement in place regarding under what future circumstances the person should stop driving. (New)

Essential	Important	Don't know/Depends	Unimportant	Should not be included
○	○	○	○	○

* 104. The helper should encourage the person to begin to plan for when they stop driving, e.g. what transport they will use, setting up automatic bill payment online and delivery services. (Rerate)

Essential	Important	Don't know/Depends	Unimportant	Should not be included
○	○	○	○	○

* 105. The helper should encourage the person to express what the loss of driving means to them because this may help the transition to not driving in the future. (Rerate)

Essential	Important	Don't know/Depends	Unimportant	Should not be included
○	○	○	○	○

Helping the confused older person - Round 2

Discussing driving and making decisions with the person (continued)

This section concerns conversations and decisions about stopping or restricting driving. In this section, the helper will generally be a family caregiver. Please rate how important (from ‘essential’ to ‘should not be included’) you think it is that each statement be included in the guidelines.

When providing first aid to the confused person, the helper will need to use their judgement because a person's decline in cognitive ability may be very mild or more severe. Please keep this in mind when rating the following items.

Please also keep the **definitions** in mind when rating the statements.

How to know when the person should stop driving

* 106. Even if the person is currently safe to drive, the helper should encourage the person diagnosed with dementia to gradually modify their driving, e.g. drive only on familiar roads, avoid long distances, avoid driving at night or in bad weather. (Rerate)

Essential	Important	Don't know/Depends	Unimportant	Should not be included
◯	◯	◯	◯	◯

* 107. Even if the person is currently safe to drive, the helper should be aware that they can become disoriented and lost, even on familiar roads, and that methods for identification and communication should be considered. (New)

Essential	Important	Don't know/Depends	Unimportant	Should not be included
◯	◯	◯	◯	◯

* 108. The helper should be aware that no examination or single indicator exists to determine when the person poses a danger to themselves or others by driving. (Rerate)

Essential	Important	Don't know/Depends	Unimportant	Should not be included
◯	◯	◯	◯	◯

* 109. The helper should be aware that a diagnosis of dementia is not automatically a reason to take away the right to drive. (New)

Essential	Important	Don't know/Depends	Unimportant	Should not be included
◯	◯	◯	◯	◯

Helping the confused older person - Round 2

Discussing driving and making decisions with the person (continued)

This section concerns conversations and decisions about stopping or restricting driving. In this section, the helper will generally be a family caregiver. Please rate how important (from ‘essential’ to ‘should not be included’) you think it is that each statement be included in the guidelines.

When providing first aid to the confused person, the helper will need to use their judgement because a person's decline in cognitive ability may be very mild or more severe. Please keep this in mind when rating the following items.

Please also keep the **definitions** in mind when rating the statements.

How to know when the person should stop driving

* 110. The helper should determine when the person should stop driving by:

	Essential	Important	Don't know/ depends	Unimportant	Should not be included
considering whether they or others feel uncomfortable driving with the person. (Rerate)	○	○	○	○	○
asking the person to have a driving assessment, e.g. occupational therapy driving assessment. (Rerate)	○	○	○	○	○

* 111. The helper should include the person, as far as possible, in decision-making regarding any driving restrictions. (Rerate)

Essential	Important	Don't know/Depends	Unimportant	Should not be included
○	○	○	○	○

* 112. If it is clear that the person can no longer drive safely, the helper should have a discussion with the person about stopping driving. (Rerate)

Essential	Important	Don't know/Depends	Unimportant	Should not be included
○	○	○	○	○

* 113. If the person has had an increase in frequency of unsafe driving incidents, the helper should:

	Essential	Important	Don't know/Depends	Unimportant	Should not be included
have a discussion with them about restricting their driving. (Rerate)	○	○	○	○	○
point out that they may get in trouble with the police or not be covered by insurance if they continue to drive. (New)	○	○	○	○	○

Helping the confused older person - Round 2

Discussing driving and making decisions with the person (continued)

This section concerns conversations and decisions about stopping or restricting driving. In this section, the helper will generally be a family caregiver. Please rate how important (from 'essential' to 'should not be included') you think it is that each statement be included in the guidelines.

When providing first aid to the confused person, the helper will need to use their judgement because a person's decline in cognitive ability may be very mild or more severe. Please keep this in mind when rating the following items.

Please also keep the **definitions** in mind when rating the statements.

How to broach the subject of driving safety with the person

* 114. The helper should use appeals about safety when talking to the person about driving in the earlier stages of their cognitive decline. (Rerate)

Essential	Important	Don't know/Depends	Unimportant	Should not be included
○	○	○	○	○

* 115. If the helper has had a frank discussion with the person about concerns over their driving ability, but the person continues to drive, the helper should ask other trusted friends or family members to help persuade the person. (New)

Essential	Important	Don't know/Depends	Unimportant	Should not be included
○	○	○	○	○

* 116. If the person does not agree to stop driving, the helper should enlist a professional mediator to help, as this will provide a neutral approach that lets the person feel heard and respected. (Rerate)

Essential	Important	Don't know/Depends	Unimportant	Should not be included
○	○	○	○	○

* 117. If the person is insistent on driving, and the helper decides to find ways to make driving impossible for the person (e.g. disabling the car in some way, hiding the keys), they should be prepared for angry or aggressive behaviour from the person and take steps to minimise any possible risk to safety. (New)

Essential	Important	Don't know/Depends	Unimportant	Should not be included
○	○	○	○	○

Helping the confused older person - Round 2

Discussing driving and making decisions with the person (continued)

This section concerns conversations and decisions about stopping or restricting driving. In this section, the helper will generally be a family caregiver. Please rate how important (from 'essential' to 'should not be included') you think it is that each statement be included in the guidelines.

When providing first aid to the confused person, the helper will need to use their judgement because a person's decline in cognitive ability may be very mild or more severe. Please keep this in mind when rating the following items.

Please also keep the **definitions** in mind when rating the statements.

How to help the person give up driving

* 118. The helper should help the person give up driving by offering alternatives, e.g. "I'll drive today and you can look at the scenery". (Rerate)

Essential	Important	Don't know/Depends	Unimportant	Should not be included
○	○	○	○	○

* 119. If the person is not convinced they should no longer drive, the helper should enlist the help of the person's doctor to write a 'prescription' to stop driving. (Rerate)

Essential	Important	Don't know/Depends	Unimportant	Should not be included
○	○	○	○	○

* 120. If the person is not convinced they should no longer drive, both the person and the helper should agree to abide by the results of a driving assessment, e.g. an occupational therapy driving assessment. (Rerate)

Essential	Important	Don't know/Depends	Unimportant	Should not be included
○	○	○	○	○

* 121. If the person is insistent on driving, the helper should find ways to make driving impossible for the person, e.g. disabling the car in some way, hiding the keys. (Rerate)

Essential	Important	Don't know/Depends	Unimportant	Should not be included
○	○	○	○	○

* 122. If the person decides to stop driving, the helper should arrange for the person to get an alternate form of identification to their driver's licence. (New)

Essential	Important	Don't know/Depends	Unimportant	Should not be included
○	○	○	○	○

Helping the confused older person - Round 2

Care discussions and decisions

This section concerns conversations and decisions about level of care and living arrangements. In this section, the helper will generally be a family caregiver. Please rate how important (from ‘essential’ to ‘should not be included’) you think it is that each statement be included in the guidelines.

When providing first aid to the confused person, the helper will need to use their judgement because a person's decline in cognitive ability may be very mild or more severe. Please keep this in mind when rating the following items.

Please also keep the **definitions** in mind when rating the statements.

Planning for future care decisions after a diagnosis

* 123. The helper should be aware that the earlier the option of residential care is explored, the better chance there is that the person's preferences for their future care can be taken into account. (Rerate)

Essential	Important	Don't know/Depends	Unimportant	Should not be included
◯	◯	◯	◯	◯

* 124. The helper should include the person in discussions concerning their care and living arrangements where possible. (Rerate)

Essential	Important	Don't know/Depends	Unimportant	Should not be included
◯	◯	◯	◯	◯

* 125. The helper should make sure the person feels included in the decision making and preparation, as this can help the transition, e.g. touring a residential care home together. (Rerate)

Essential	Important	Don't know/Depends	Unimportant	Should not be included
◯	◯	◯	◯	◯

* 126. The helper should encourage the person who has received a diagnosis of dementia to make decisions in advance about their future care, e.g. what the person would want to happen if their current living arrangement became too difficult. (Rerate)

Essential	Important	Don't know/Depends	Unimportant	Should not be included
◯	◯	◯	◯	◯

* 127. In planning for future care decisions with the person, the helper should consider and discuss with the person the range of factors that may impact upon care and living arrangements during the course of the disease, e.g. the health of family members, financial matters, the different stages of the decline. (Rerate)

Essential	Important	Don't know/Depends	Unimportant	Should not be included
◯	◯	◯	◯	◯

* 128. The helper should talk to the person's health professional early on about what can be done to smooth the transition to residential care, if there is an eventual need. (Rerate)

Essential	Important	Don't know/Depends	Unimportant	Should not be included
◯	◯	◯	◯	◯

* 129. The helper should familiarise themselves with any eligibility criteria for residential care or other services that the person may need in the future. (Rerate)

Essential	Important	Don't know/Depends	Unimportant	Should not be included
◯	◯	◯	◯	◯

* 130. The helper should consider introducing outside help or support services to help care for the person in the early stages of the person's dementia, because it may be accepted more easily at this stage than later on. (Rerate)

Essential	Important	Don't know/Depends	Unimportant	Should not be included
○	○	○	○	○

Helping the confused older person - Round 2

Care discussions and decisions (continued)

This section concerns conversations and decisions about level of care and living arrangements. In this section, the helper will generally be a family caregiver. Please rate how important (from ‘essential’ to ‘should not be included’) you think it is that each statement be included in the guidelines.

When providing first aid to the confused person, the helper will need to use their judgement because a person's decline in cognitive ability may be very mild or more severe. Please keep this in mind when rating the following items.

Please also keep the **definitions** in mind when rating the statements.

How to know when the person needs a higher level of care or a more supported living arrangement

* 131. When deciding whether it is time to change to a higher level of care or a more supported living arrangement for the person, the helper should consider whether:

	Essential	Important	Don't know/ depends	Unimportant	Should not be included
the person's communication has changed, e.g. phone calls have slowed or ceased, non-emergency calls are made at unreasonable hours. (Rerate)	○	○	○	○	○
there are changes in the person's social life, e.g. disengaged from previous social activities or relationships, forgetting appointments. (Rerate)	○	○	○	○	○
the person cannot handle their day-to-day financial transactions. (Rerate)	○	○	○	○	○
the family carergivers' negative feelings about being with the person have begun to outweigh the positive feelings. (Rerate)	○	○	○	○	○
the person is no longer able to recognise or interact with their environment. (Rerate)	○	○	○	○	○
there are others who can help with caregiving in the person's current living situation. (Rerate)	○	○	○	○	○

How to handle difficulties on the decision for residential care

* 132. The helper should be aware that moving the person to residential care may not necessarily mean less time devoted to caring for the person, as they may still need to be involved in their care, e.g. checking medications are correct, that daily needs are being met. (New)

Essential	Important	Don't know/Depends	Unimportant	Should not be included
○	○	○	○	○

* 133. If the person does not want to move into residential care, but it is clearly needed, the helper should consider asking the person's health care professional to take the 'blame' for the move into residential care. (New)

Essential	Important	Don't know/Depends	Unimportant	Should not be included
◯	◯	◯	◯	◯

* 134. The helper should discuss the decision to move the person to residential care with a helpline or their local dementia support organisation. (New)

Essential	Important	Don't know/Depends	Unimportant	Should not be included
◯	◯	◯	◯	◯

* 135. If the person attempts to get out of newly made arrangements, the helper should validate any distress the person is feeling about the new arrangement, but reinforce the non-negotiable nature of the new arrangement, e.g. "I realise it is upsetting that you can't live at home, but it is no longer safe for you to do so". (New)

Essential	Important	Don't know/Depends	Unimportant	Should not be included
◯	◯	◯	◯	◯

Helping the confused older person - Round 2

Challenging Behaviours

This section asks you what information the helper should know in order to assist a person whose behaviour they are finding challenging.

Challenging behaviours *refers to behaviours which are difficult, inappropriate, undesirable or unacceptable. For example, this may include agitated, repetitive, aggressive or disinhibited behaviours.*

Please rate how important (from ‘essential’ to ‘should not be included’) you think it is that each statement be included in the guidelines.

When providing first aid to the confused person, the helper will need to use their judgement because a person's decline in cognitive ability may be very mild or more severe. Please keep this in mind when rating the following items.

Please also keep the **definitions** in mind when rating the statements.

General challenging behaviours

* 136. The helper should be aware that the person may display challenging behaviours in order to gain a greater sense of control over their life. (Rerate)

Essential	Important	Don't know/Depends	Unimportant	Should not be included
◯	◯	◯	◯	◯

* 137. The helper should be aware that there may be underlying psychological issues other than dementia that may cause challenging behaviour to increase, such as anxiety or depression. (New)

Essential	Important	Don't know/Depends	Unimportant	Should not be included
◯	◯	◯	◯	◯

* 138. If the person is displaying challenging behaviour that the helper wants to stop, the helper should interrupt what the person is doing or distract the person by doing a physical activity with the person. (New)

Essential	Important	Don't know/Depends	Unimportant	Should not be included
◯	◯	◯	◯	◯

* 139. If the person is displaying challenging behaviour that the helper wants to stop, **and the helper cannot work out what needs the person is trying to communicate**, the helper should interrupt what the person is doing or distract the person, e.g. ask the person a question about their early life. (New)

Essential	Important	Don't know/Depends	Unimportant	Should not be included
◯	◯	◯	◯	◯

Helping the confused older person - Round 2

Challenging Behaviours (continued)

This section asks you what information the helper should know in order to assist a person whose behaviour they are finding challenging.

Challenging behaviours *refers to behaviours which are difficult, inappropriate, undesirable or unacceptable. For example, this may include agitated, repetitive, aggressive or disinhibited behaviours.*

Please rate how important (from ‘essential’ to ‘should not be included’) you think it is that each statement be included in the guidelines.

When providing first aid to the confused person, the helper will need to use their judgement because a person's decline in cognitive ability may be very mild or more severe. Please keep this in mind when rating the following items.

Please also keep the **definitions** in mind when rating the statements.

Repetitive behaviour

Repetitive behaviour *includes the repetition of questions, phrases, movements or actions, e.g. pacing.*

* 140. If the person asks the same question over and over, the helper should:

	Essential	Important	Don't know/Depends	Unimportant	Should not be included
politely answer the first few times and then use distraction to change the topic. (Rerate)	◯	◯	◯	◯	◯
try to guess what the person's underlying concern is and reassure them about this, e.g. a repeated question about the time might be because they are worried they will miss an event. (Rerate)	◯	◯	◯	◯	◯
answer them and ask the person to repeat the answer back in order to help them remember it. (New)	◯	◯	◯	◯	◯

Helping the confused older person - Round 2

Challenging Behaviours (continued)

This section asks you what information the helper should know in order to assist a person whose behaviour they are finding challenging.

Challenging behaviours *refers to behaviours which are difficult, inappropriate, undesirable or unacceptable. For example, this may include agitated, repetitive, aggressive or disinhibited behaviours.*

Please rate how important (from ‘essential’ to ‘should not be included’) you think it is that each statement be included in the guidelines.

When providing first aid to the confused person, the helper will need to use their judgement because a person's decline in cognitive ability may be very mild or more severe. Please keep this in mind when rating the following items.

Please also keep the **definitions** in mind when rating the statements.

When the person is agitated, angry or upset

* 141. If the person becomes agitated, the helper should patiently wait for the behaviour to pass, provided the person or others are not at risk of harm. If the person is asking repetitive questions, the helper should answer them and ask the person to repeat the answer back in order to help them remember it. (New)

Essential	Important	Don't know/Depends	Unimportant	Should not be included
◯	◯	◯	◯	◯

* 142. If the person becomes agitated during conversation, the helper should change to another activity or topic of conversation thereby removing the situation that is causing the agitation. (Rerate)

Essential	Important	Don't know/Depends	Unimportant	Should not be included
○	○	○	○	○

* 143. If the person is frightened or agitated, the helper should be careful about touching the person because the person might interpret the touch as a form of restraint and become angry. (Rerate)

Essential	Important	Don't know/Depends	Unimportant	Should not be included
○	○	○	○	○

* 144. The helper should not physically restrain the person because they may feel fenced in and **become aggressive**. (Rerate)

Essential	Important	Don't know/Depends	Unimportant	Should not be included
○	○	○	○	○

* 145. The helper should not physically restrain the person because they may feel fenced in and **become more agitated**. (New)

Essential	Important	Don't know/Depends	Unimportant	Should not be included
○	○	○	○	○

* 146. The helper should only physically restrain the person if there is risk of harm to self or others and nothing else works. (Rerate)

Essential	Important	Don't know/Depends	Unimportant	Should not be included
○	○	○	○	○

* 147. If the person appears anxious about something, the helper should offer comfort and reassurance rather than responding to the specifics of what the person is trying to say. (Rerate)

Essential	Important	Don't know/Depends	Unimportant	Should not be included
○	○	○	○	○

* 148. If the person is pacing and it is safe to do so, the helper should let them pace. (New)

Essential	Important	Don't know/Depends	Unimportant	Should not be included
○	○	○	○	○

* 149. If the person becomes aggressive, the helper should leave the room, if it is safe to do so, and give the person the time and space to calm down. (New)

Essential	Important	Don't know/Depends	Unimportant	Should not be included
○	○	○	○	○

Helping the confused older person - Round 2

Challenging Behaviours (continued)

This section asks you what information the helper should know in order to assist a person whose behaviour they are finding challenging.

Challenging behaviours *refers to behaviours which are difficult, inappropriate, undesirable or unacceptable. For example, this may include agitated, repetitive, aggressive or disinhibited behaviours.*

Please rate how important (from 'essential' to 'should not be included') you think it is that each statement be included in the guidelines.

When providing first aid to the confused person, the helper will need to use their judgement because a person's decline in cognitive ability may be very mild or more severe. Please keep this in mind when rating the following items.

Please also keep the **definitions** in mind when rating the statements.

Disinhibited behaviours

Disinhibited behaviours *are actions which seem tactless, rude or offensive. They occur when people don't follow the usual social rules about what or where to say or do something.*

* 150. If the person is behaving in a disinhibited way, the helper should:

	Essential	Important	Don't know/Depends	Unimportant	Should not be included
not show shock or disapproval. (New)	○	○	○	○	○
gently correct the person, for example, say 'I don't feel comfortable when you do that. Please stop.' (New)	○	○	○	○	○
guide them to a private place. (New)	○	○	○	○	○

* 151. If the person engages in inappropriate sexual behaviour (e.g. public masturbation), the helper should:

	Essential	Important	Don't know/Depends	Unimportant	Should not be included
gently correct the person, for example, say 'I don't feel comfortable when you do that. Please stop.' (New)	◯	◯	◯	◯	◯
try to identify their needs and find other more appropriate ways to meet them, e.g. offering tactile experiences, nicer/more interesting smells, tastes, sounds etc. (New)	◯	◯	◯	◯	◯
remind the person that the behaviour is inappropriate and guide them to a private place. (Rerate)	◯	◯	◯	◯	◯

Helping the confused older person - Round 2

Challenging Behaviours (continued)

This section asks you what information the helper should know in order to assist a person whose behaviour they are finding challenging.

Challenging behaviours *refers to behaviours which are difficult, inappropriate, undesirable or unacceptable. For example, this may include agitated, repetitive, aggressive or disinhibited behaviours.*

Please rate how important (from ‘essential’ to ‘should not be included’) you think it is that each statement be included in the guidelines.

When providing first aid to the confused person, the helper will need to use their judgement because a person's decline in cognitive ability may be very mild or more severe. Please keep this in mind when rating the following items.

Please also keep the **definitions** in mind when rating the statements.

Different realities, hallucinations and delusions

Hallucinations *are false perceptions. A person who is experiencing an hallucination might hear, see, feel or taste things that are not actually there.*

Delusions *are fixed false beliefs. Although the delusions might seem bizarre, they are very real to the person experiencing them.*

Confabulation *is the filling in of gaps in a person's memory with fabrications that they believes to be true.*

* 152. If the person is deluded that someone is stealing from them, the helper should remain neutral, e.g. "You may be right. I will ask them about it tomorrow." (New)

Essential	Important	Don't know/Depends	Unimportant	Should not be included
◯	◯	◯	◯	◯

* 153. If the person is making unfounded accusations, the helper should deny their involvement in the accusation, e.g. "You know that I have never stolen from you." (New)

Essential	Important	Don't know/Depends	Unimportant	Should not be included
◯	◯	◯	◯	◯

* 154. If the person is hallucinating, the helper should:

	Essential	Important	Don't know/Depends	Unimportant	Should not be included
ask about the feelings behind the hallucinations and empathise with the person. (Rerate)	◯	◯	◯	◯	◯
check if the person has had enough to eat and drink and had enough sleep. (Rerate)	◯	◯	◯	◯	◯

* 155. If the person has hallucinations or delusions, and the person is very distressed and cannot be calmed down, the helper should contact the person's doctor. (Rerate)

Essential	Important	Don't know/Depends	Unimportant	Should not be included
◯	◯	◯	◯	◯

Helping the confused older person - Round 2

Delirium

This section asks you what information the helper should know in order to assist and interact with a person who has delirium. Please rate how important (from ‘essential’ to ‘should not be included’) you think it is that each statement be included in the guidelines.

Delirium *is a condition where a sudden and obvious worsening of a person’s usual level of functioning appears over hours or days. Delirium can involve problems with attention, awareness, orientation to environment and other areas of cognitive functioning. Delirium is due to illness or environmental factors such as medication.*

When providing first aid to the confused person, the helper will need to use their judgement because a person's decline in cognitive ability may be very mild or more severe. Please keep this in mind when rating the following items.

Please also keep the **definitions** in mind when rating the statements.

How to assist the person with suspected delirium

* 156. The helper should be aware that delirium is a medical emergency requiring immediate medical help. (New)

Essential	Important	Don't know/Depends	Unimportant	Should not be included
○	○	○	○	○

* 157. The helper should be aware that a person with dementia is more likely to develop delirium. (New)

Essential	Important	Don't know/Depends	Unimportant	Should not be included
○	○	○	○	○

* 158. The helper should be aware that any sudden cognitive deterioration in the person is likely to be a result of a reversible illness that requires immediate attention. (New)

Essential	Important	Don't know/Depends	Unimportant	Should not be included
○	○	○	○	○

* 159. If the helper suspects the person is experiencing delirium and their behaviour is a serious risk to their safety and the safety of others, the helper should take the person to hospital or call an ambulance. (Rerate)

Essential	Important	Don't know/Depends	Unimportant	Should not be included
○	○	○	○	○

* 160. If the helper suspects the person is experiencing delirium, the helper should contact a doctor immediately to inform them of the sudden changes in the person and arrange an appointment. (Rerate)

Essential	Important	Don't know/Depends	Unimportant	Should not be included
○	○	○	○	○

* 161. If the helper is caring for a person with delirium, they should inform any visitors of what to expect. (Rerate)

Essential	Important	Don't know/Depends	Unimportant	Should not be included
○	○	○	○	○

* 162. To reduce the risk of falls, the helper should assist the person experiencing delirium with walking and moving from a sitting or lying position. (Rerate)

Essential	Important	Don't know/Depends	Unimportant	Should not be included
○	○	○	○	○

Helping the confused older person - Round 2

Thank you

If something in these surveys caused you to feel distressed or upset then we advise you to talk to a supportive person about these feelings. You might wish to talk to someone using the relevant helpline listed below.

Australia
National Dementia Helpline
1800 100 500

New Zealand
Local Alzheimers New Zealand organisations
0800 004 001

Canada
Alzheimer Society of B.C. Dementia Helpline
Province-wide: 1-800-936-6033
Lower Mainland: 604-681-8651

Ireland
Alzheimer National Helpline
1800 341 341

United Kingdom
National Dementia Helpline
0300 222 1122

United States
Alzheimer's Association 24/7 Helpline
1.800.272.3900

That is the end of the Round 2 survey!

Thank you very much for your contribution.

By pressing the "next" button your final responses will be registered with our survey software. Once all panel members have lodged their responses, we will collate the data and send you a report on the findings and the third and final survey.

We are extremely grateful for your contribution.

Best Wishes,
The Centre for Mental Health, University of Melbourne and Mental Health First Aid Australia Research Team

Helping the confused older person - Round 3

INTRODUCTION

Instructions

The statements in this questionnaire were derived from the results of the Round 2 survey.

Please complete the questionnaire by rating each statement **according to how important you believe it is for inclusion in the guidelines** for helping a confused older person. Please keep in mind that the guidelines will be used by the general public and as such, the statements need to be rated according to how important each one is for someone, who does not necessarily have a counselling or clinical background, to help an older person with confusion.

This questionnaire should take approximately 20 minutes to complete. You can complete the survey in two or more sittings. Your answers are saved when you click *'Next'* at the bottom of a page. This marks your page and you can begin again at a later date on the next page. **Please be aware that once you have logged on and started responding you must complete the questionnaire on the same computer.**

* 1. Please provide your name.

____

* 2.

Area of expertise

- ○ Carer advocate
- ○ Professional

Helping the confused older person - Round 3

Overview of Questionnaire

The questionnaire is divided into the following sections:

1. Memory and other cognitive problems

2. Seeking help

3. Encountering someone wandering

4. Communication

5. Challenges you may encounter during communication

6. Discussing sensitive issues and making decisions with the person

7. Challenging behaviours

Helping the confused older person - Round 3

Definitions

These terms may have different meanings for participants. The definitions below are how these terms are used in this survey. When completing the survey, please ensure you use these definitions only.

Person in this survey refers to an **older person who is experiencing confusion** which may be due to dementia, delirium or other conditions. Older person in this survey refers to those aged 65 or older, however it is expected that the resulting guidelines may also be relevant to assisting adults with confusion who are younger.

Mental health first aid for the confused older person is the help offered to a person who may be developing dementia or delirium, is experiencing a worsening of existing dementia symptoms or is in a crisis due to their confusion.

Helper in this survey refers to the individual who provides mental health first aid as defined above. **The helper may be a family member, friend, neighbour, concerned community member or a paid carer without specialist qualifications.**

Confusion in this survey is a broad term that refers to a decline in normal cognitive ability, which may vary from mild to severe. The cognitive changes may be associated with dementia or delirium. It may include a number of the following signs and symptoms: lack of alertness, poor attention span, disorientation to time and place, trouble following a conversation, unclear or illogical speech, impaired short-term memory, difficulty in planning and carrying out tasks, inappropriate behaviour, disconnection from reality or delusional beliefs.

Dementia is a condition involving progressive decline of cognitive abilities such as short-term memory, language and the ability to plan and carry out tasks. Dementia is an umbrella term for a large group of illnesses that cause this progressive decline. The symptoms appear over months and years. The initial signs of confusion may be mild, or may be only apparent at certain times or in certain situations, but the condition tends to get worse over time.

Delirium is a condition where a sudden and obvious worsening of a person’s usual level of functioning appears over hours or days. Delirium can involve problems with attention, awareness, orientation to environment and other areas of cognitive functioning. Delirium is caused by an underlying disease or environmental factors, such as medication.

Helping the confused older person - Round 3

Memory and other cognitive problems

This section asks you what information the helper should know about memory and other cognitive problems. It also asks how the helper can assist the person with these limitations. Please rate how important (from ‘essential’ to ‘should not be included’) you think it is that each statement be included in the guidelines.

When providing first aid to the confused person, the helper will need to use their judgement because a person's decline in cognitive ability may be very mild or more severe. Please keep this in mind when rating the following items.

Please also keep the **definitions** in mind when rating the statements.

* 3. If the person is going to be in an unfamiliar place, the helper should ensure that the person has some familiar people with them to reduce the chance of confusion. (Rerate)

Essential	Important	Don't know/Depends	Unimportant	Should not be included
○	○	○	○	○

* 4. If the person does not recognise what an object is for, the helper should consider using step-by-step prompts. (Rerate)

Essential	Important	Don't know/Depends	Unimportant	Should not be included
○	○	○	○	○

* 5. If the person is struggling with a task (e.g. dressing), the helper should consider using the following strategies:

	Essential	Important	Don't know/Depends	Unimportant	Should not be included
acknowledging the person's frustration. (Rerate)	○	○	○	○	○
using step-by-step prompts. (Rerate)	○	○	○	○	○
demonstrating how to do it. (Rerate)	○	○	○	○	○

* 6. If the person talks about deceased people as though they are alive (e.g. a parent who has died long ago) and they are distressed, the helper should consider engaging in a conversation about the deceased person. (Rerate)

Essential	Important	Don't know/Depends	Unimportant	Should not be included
○	○	○	○	○

* 7. If the person talks about deceased people as though they are alive (e.g. a parent who has died long ago) and they are **not** distressed, the helper should consider engaging in a conversation about the deceased person. (Rerate)

Essential	Important	Don't know/Depends	Unimportant	Should not be included
○	○	○	○	○

Helping the confused older person - Round 3

Seeking help

This section asks you what information the helper should know about when and how the helper can assist the person to get professional help. Please rate how important (from ‘essential’ to ‘should not be included’) you think it is that each statement be included in the guidelines.

When providing first aid to the confused person, the helper will need to use their judgement because a person's decline in cognitive ability may be very mild or more severe. Please keep this in mind when rating the following items.

Please also keep the **definitions** in mind when rating the statements.

* 8. If the person is reluctant to get help, the helper should identify another individual who has a good relationship with the person to support them to seek help. (Rerate)

Essential	Important	Don't know/Depends	Unimportant	Should not be included
◯	◯	◯	◯	◯

Helping the confused older person - Round 3

Encountering someone who is wandering

This section asks you what information the helper should know in order to assist a person who is wandering.

Wandering *is a dementia-related behaviour that sees a disoriented individual move about, sometimes with repetitive pacing or lapping in one area, and other times leaving their usual environment. A wandering person may become lost, leave a safe environment or intrude in inappropriate places.*

Please rate how important (from 'essential' to 'should not be included') you think it is that each statement be included in the guidelines.

When providing first aid to the confused person, the helper will need to use their judgement because a person's decline in cognitive ability may be very mild or more severe. Please keep this in mind when rating the following items.

Please also keep the **definitions** in mind when rating the statements.

* 9. The helper should not leave the person who is wandering alone, even if the person declines their help. (Rerate)

Essential	Important	Don't know/Depends	Unimportant	Should not be included
◯	◯	◯	◯	◯

Helping the confused older person - Round 3

Communication

This section asks you what information the helper should know in order to communicate effectively with a confused person. Please rate how important (from 'essential' to 'should not be included') you think it is that each statement be included in the guidelines.

When providing first aid to the confused person, the helper will need to use their judgement because a person's decline in cognitive ability may be very mild or more severe. Please keep this in mind when rating the following items.

Please also keep the **definitions** in mind when rating the statements.

* 10. If the helper cannot get the person's attention and the conversation is not important, they should leave it for another time. (Rerate)

Essential	Important	Don't know/Depends	Unimportant	Should not be included
◯	◯	◯	◯	◯

* 11. When talking to a person the helper should tailor their use of language and vocabulary to be similar to the person's own. (Rerate)

Essential	Important	Don't know/Depends	Unimportant	Should not be included
○	○	○	○	○

Helping the confused older person - Round 3

Challenges you may encounter during communication

This section asks you what information the helper should know in order to respond to challenges that may occur whilst communicating with a confused person. Please rate how important (from ‘essential’ to ‘should not be included’) you think it is that each statement be included in the guidelines.

When providing first aid to the confused person, the helper will need to use their judgement because a person's decline in cognitive ability may be very mild or more severe. Please keep this in mind when rating the following items.

Please also keep the **definitions** in mind when rating the statements.

* 12. If the person uses the wrong word, the helper should not point out the error but repeat back the person's message using the correct word. (Rerate)

Essential	Important	Don't know/Depends	Unimportant	Should not be included
○	○	○	○	○

* 13. If an argument develops **over a minor matter**, the helper should:

	Essential	Important	Don't know/Depends	Unimportant	Should not be included
apologise and then let the matter be. (Rerate)	○	○	○	○	○
change the topic of conversation or begin a new activity. (Rerate)	○	○	○	○	○

* 14. If an argument develops, the helper should change the topic of conversation or begin a new activity. (Rerate)

Essential	Important	Don't know/Depends	Unimportant	Should not be included
○	○	○	○	○

Helping the confused older person - Round 3

Discussing sensitive issues and making decisions with the person

This section concerns conversations about sensitive issues which might include when to see a doctor about symptoms of dementia, stopping driving or the need for a change in level of care or living arrangements. In this section, 'a conversation' refers to a conversation about a sensitive issue. Please rate how important (from 'essential' to 'should not be included') you think it is that each statement be included in the guidelines.

When providing first aid to the confused person, the helper will need to use their judgement because a person's decline in cognitive ability may be very mild or more severe. Please keep this in mind when rating the following items.

Please also keep the **definitions** in mind when rating the statements.

* 15. When discussing a sensitive topic, the helper should consider enlisting the help of another person who can remain calm and objective. (Rerate)

Essential	Important	Don't know/Depends	Unimportant	Should not be included
○	○	○	○	○

* 16. If the person shows negative emotions (e.g. tearful, angry), the helper should take them somewhere private where the person can express this without feeling embarrassed. (Rerate)

Essential	Important	Don't know/Depends	Unimportant	Should not be included
○	○	○	○	○

* 17. If the person does not understand all that their diagnosis means or they deny it, the helper should accept their reaction. (Rerate)

Essential	Important	Don't know/Depends	Unimportant	Should not be included
○	○	○	○	○

Helping the confused older person - Round 3

Discussing driving and making decisions with the person

This section concerns conversations and decisions about stopping or restricting driving. In this section, the helper will generally be a family caregiver. Please rate how important (from 'essential' to 'should not be included') you think it is that each statement be included in the guidelines.

When providing first aid to the confused person, the helper will need to use their judgement because a person's decline in cognitive ability may be very mild or more severe. Please keep this in mind when rating the following items.

Please also keep the **definitions** in mind when rating the statements.

* 18. Shortly after diagnosis, the helper should begin discussing and planning**with the person** about when they should stop driving. (Rerate)

Essential	Important	Don't know/Depends	Unimportant	Should not be included
○	○	○	○	○

* 19. If the person decides to stop driving, the helper should arrange for the person to get an alternate form of identification to their driver's licence. (Rerate)

Essential	Important	Don't know/Depends	Unimportant	Should not be included
◯	◯	◯	◯	◯

Helping the confused older person - Round 3

Care discussions and decisions

This section concerns conversations and decisions about level of care and living arrangements. In this section, the helper will generally be a family caregiver. Please rate how important (from 'essential' to 'should not be included') you think it is that each statement be included in the guidelines.

When providing first aid to the confused person, the helper will need to use their judgement because a person's decline in cognitive ability may be very mild or more severe. Please keep this in mind when rating the following items.

Please also keep the **definitions** in mind when rating the statements.

How to handle difficulties on the decision for residential care

* 20. The helper should be aware that moving the person to residential care may not necessarily mean less time devoted to caring for the person, as they may still need to be involved in their care, e.g. checking medications are correct, that daily needs are being met. (Rerate)

Essential	Important	Don't know/Depends	Unimportant	Should not be included
◯	◯	◯	◯	◯

* 21. If the person attempts to get out of newly made arrangements, the helper should validate any distress the person is feeling about the new arrangement, but reinforce the non-negotiable nature of the new arrangement, e.g. "I realise it is upsetting that you can't live at home, but it is no longer safe for you to do so". (Rerate)

Essential	Important	Don't know/Depends	Unimportant	Should not be included
◯	◯	◯	◯	◯

Helping the confused older person - Round 3

Challenging behaviours

This section asks you what information the helper should know in order to assist a person whose behaviour they are finding challenging.

Challenging behaviours *refers to behaviours which are difficult, inappropriate, undesirable or unacceptable. For example, this may include agitated, repetitive, aggressive or disinhibited behaviours.*

Please rate how important (from 'essential' to 'should not be included') you think it is that each statement be included in the guidelines.

When providing first aid to the confused person, the helper will need to use their judgement because a person's decline in cognitive ability may be very mild or more severe. Please keep this in mind when rating the following items.

Please also keep the **definitions** in mind when rating the statements.

When the person is agitated, angry or upset

* 22. If the person becomes aggressive, the helper should leave the room, if it is safe to do so, and give the person the time and space to calm down. (Rerate)

Essential	Important	Don't know/Depends	Unimportant	Should not be included
○	○	○	○	○

Disinhibited behaviours

Disinhibited behaviours *are actions which seem tactless, rude or offensive. They occur when people don't follow the usual social rules about what or where to say or do something.*

* 23. If the person is behaving in a disinhibited way, the helper should:

	Essential	Important	Don't know/Depends	Unimportant	Should not be included
not show shock or disapproval. (Rerate)	○	○	○	○	○
guide them to a private place. (Rerate)	○	○	○	○	○

* 24. If the person engages in inappropriate sexual behaviour (e.g. public masturbation), the helper should consider ways to modify the environment to reduce the triggers for this behaviour, e.g., not going out in public at times when the problem behaviour is more likely to occur, having a different person help with showering. (*New item - this item came from a book that was published since our initial literature search. We thought that it was important to include it given only a few items about sexual behaviour have been endorsed. This item will only be rated once.*)

Essential	Important	Don't know/Depends	Unimportant	Should not be included
○	○	○	○	○

Helping the confused older person - Round 3

Thank you

If something in these surveys caused you to feel distressed or upset then we advise you to talk to a supportive person about these feelings. You might wish to talk to someone using the relevant helpline listed below.

Australia
National Dementia Helpline
1800 100 500

New Zealand
Local Alzheimers New Zealand organisations
0800 004 001

Canada
Alzheimer Society of B.C. Dementia Helpline
Province-wide: 1-800-936-6033
Lower Mainland: 604-681-8651

Ireland
Alzheimer National Helpline
1800 341 341

United Kingdom
National Dementia Helpline
0300 222 1122

United States
Alzheimer's Association 24/7 Helpline
1.800.272.3900

That is the end of the Round 3 survey!

Thank you very much for your contribution.

By pressing the "next" button your final responses will be registered with our survey software. Once all panel members have lodged their responses, we will collate the data and send you a report on the findings of this final survey.

We are extremely grateful for your contribution.

Best Wishes,
The Centre for Mental Health, University of Melbourne and Mental Health First Aid Australia Research Team
